# Phosphate-regulated expression of the SARS-CoV-2 receptor-binding domain in the diatom *Phaeodactylum tricornutum* for pandemic diagnostics

**DOI:** 10.1038/s41598-022-11053-7

**Published:** 2022-04-29

**Authors:** Samuel S. Slattery, Daniel J. Giguere, Emily E. Stuckless, Arina Shrestha, Lee-Ann K. Briere, Alexa Galbraith, Stephen Reaume, Xenia Boyko, Henry H. Say, Tyler S. Browne, Mallory I. Frederick, Jeremy T. Lant, Ilka U. Heinemann, Patrick O’Donoghue, Liann Dsouza, Steven Martin, Peter Howard, Christopher Jedeszko, Kinza Ali, Garth Styba, Martin Flatley, Bogumil J. Karas, Gregory B. Gloor, David R. Edgell

**Affiliations:** 1 Department of Biochemistry, Schlich School of Medicine and Dentistry, Western University, London, ON N6A 5C1 Canada; 2 Department of Chemistry, Western University, London, ON N6A 3K7 Canada; 3 Lambton College, 1457 London Rd, Sarnia, ON N7S 6K4 Canada; 4 Pond Technologies Inc., Markham, ON L3R 9W7 Canada; 5 International Point of Care Inc., 135 The West Mall Unit 9, Toronto, ON M9C 1C2 Canada; 6 Suncor Energy Inc., Sarnia Refinery, 1900 River Road, Sarnia, ON N7T 7J3 Canada

**Keywords:** Biotechnology, Expression systems, Plant sciences, Plant biotechnology, Molecular engineering in plants, Biological techniques, Genetic engineering

## Abstract

The worldwide COVID-19 pandemic caused by the SARS-CoV-2 betacoronavirus has highlighted the need for a synthetic biology approach to create reliable and scalable sources of viral antigen for uses in diagnostics, therapeutics and basic biomedical research. Here, we adapt plasmid-based systems in the eukaryotic microalgae *Phaeodactylum tricornutum* to develop an inducible overexpression system for SARS-CoV-2 proteins. Limiting phosphate and iron in growth media induced expression of the receptor-binding domain (RBD) of the SARS-CoV-2 spike protein from the *P. tricornutum* *HASP1* promoter in the wild-type strain and in a histidine auxotrophic strain that alleviates the requirement for antibiotic selection of expression plasmids. The RBD was purified from whole cell extracts (algae-RBD) with yield compromised by the finding that 90–95% of expressed RBD lacked the genetically encoded C-terminal 6X-histidine tag. Constructs that lacked the TEV protease site between the RBD and C-terminal 6X-histidine tag retained the tag, increasing yield. Purified algae-RBD was found to be *N*-linked glycosylated by treatment with endoglycosidases, was cross-reactive with anti-RBD polyclonal antibodies, and inhibited binding of recombinant RBD purified from mammalian cell lines to the human ACE2 receptor. We also show that the algae-RBD can be used in a lateral flow assay device to detect SARS-CoV-2 specific IgG antibodies from donor serum at sensitivity equivalent to assays performed with RBD made in mammalian cell lines. Our study shows that *P. tricornutum* is a scalable system with minimal biocontainment requirements for the inducible production of SARS-CoV-2 or other coronavirus antigens for pandemic diagnostics.

## Introduction

The COVID-19 pandemic caused by the SARS-CoV-2 betacoronavirus^1,2^ has exposed weaknesses in the ability of the biomedical community to respond to this and future pandemics. In particular, a major barrier to “flattening the curve” has been the availability of cheap, reliable and serologically-reactive sources of SARS-CoV-2 proteins for use in diagnostic testing. Four major structural proteins are encoded by the SARS-CoV-2 genome; the spike glycoprotein (S), membrane, envelope, and nucleocapsid (N)^3^. Current serological tests, including lateral-flow assays (LFAs), detect antibodies that recognize epitopes on the S and N proteins^4^. The receptor-binding domain (RBD) of the S1 subunit of the S glycoprotein binds to the angiotension-converting-enzyme 2 (ACE2) receptor on cells to promote viral entry^5,6^. The RBD is thus an important target for diagnostics and vaccine development because a subset of antibodies that bind to the RBD prevent viral entry and neutralize infectivity^7^. Evidence from the SARS-CoV-2 virus and other SARS-family viruses suggests that *N*-linked glycosylation of the viral spike protein is critical for the human immune system to protect against the virus. The RBD is glycosylated at two positions, N331 and N343, and consists of a mixture of complex glycans including predominantly fucoslyated glycans at N343^8^. Any recombinantly-made protein used in diagnostic tests must thus contain the appropriate *N*-linked glycosylations.

Typical protein overproduction systems include *Escherichia coli*, *Saccharomyces cerevisiae*, *Pichia pistoris*, mammalian cell lines and some plant species, and many of these systems have been used to produce SARS-CoV-2 proteins^9–16^. *E. coli* and *S. cerevisiae* lack suitable glycosylation machinery and thus are not appropriate for production of serologically reactive SARS-CoV-2 proteins, while mammalian cell lines require resource-intensive infrastructure and biocontainment. As an emerging orthogonal protein production system^17,18^, the marine pennate diatom *P. tricornutum* has a number of advantages over other systems. *P. tricornutum* is a genetically tractable organism with a small genome, a defined liquid growth media with requirements for light and carbon dioxide, and scalable bioreactor culturing. Recent advancements in genetic tool development, such as efficient DNA delivery methods^19^, replicating plasmids^19–21^, antibiotic selection markers^22–25^, inducible promoters^26–28^, natural or synthetic whole chromosome cloning^29,30^, and gene-editing systems^31–33^, have enhanced the utility of *P. tricornutum* as an orthogonal production system. The predicted *P. tricornutum* glycosylation pathway includes orthologs of mammalian enzymes^34–36^, notably the FucT fucosyltransferase enzyme necessary for core fucosylation of asparagine residues. Exogenous proteins produced in mammalian systems that are post-translationally modified with *N*-linked glycosylation have also been produced with similar modifications in *P. tricornutum*, suggesting that the algal-produced proteins are immunogenic and functional^37–39^.

Here, we adapt *P. tricornutum* plasmid-based systems for the overexpression and purification of the SARS-CoV-2 RBD. We describe phosphate and iron regulation of the *HASP1* promoter for regulated synthesis of the RBD and other proteins from replicating plasmids. Overexpressed RBD is retained intracellularly and is *N*-linked glycosylated as indicated by treatment with the endoglycosidases PNGase F and Endo H. The algae-RBD can competitively inhibit binding of recombinant RBD produced in mammalian cell culture to the human ACE2 extracellular signalling domain. Importantly, the algae-RBD can also be used in a lateral flow device to detect IgG antibodies against SARS-CoV-2 in serum at sensitivities equivalent to tests performed with RBD sourced from mammalian expression systems. Our results provide proof-of-principle that *P. tricornutum* expression plasmids based on the *HASP1* promoter represent an orthogonal, simple, scalable and regulated system for the production of SARS-CoV-2 antigens for pandemic diagnostics.

## Results

### Stable maintenance of RBD-expressing plasmids in *P. tricornutum*

**Figure 1:**
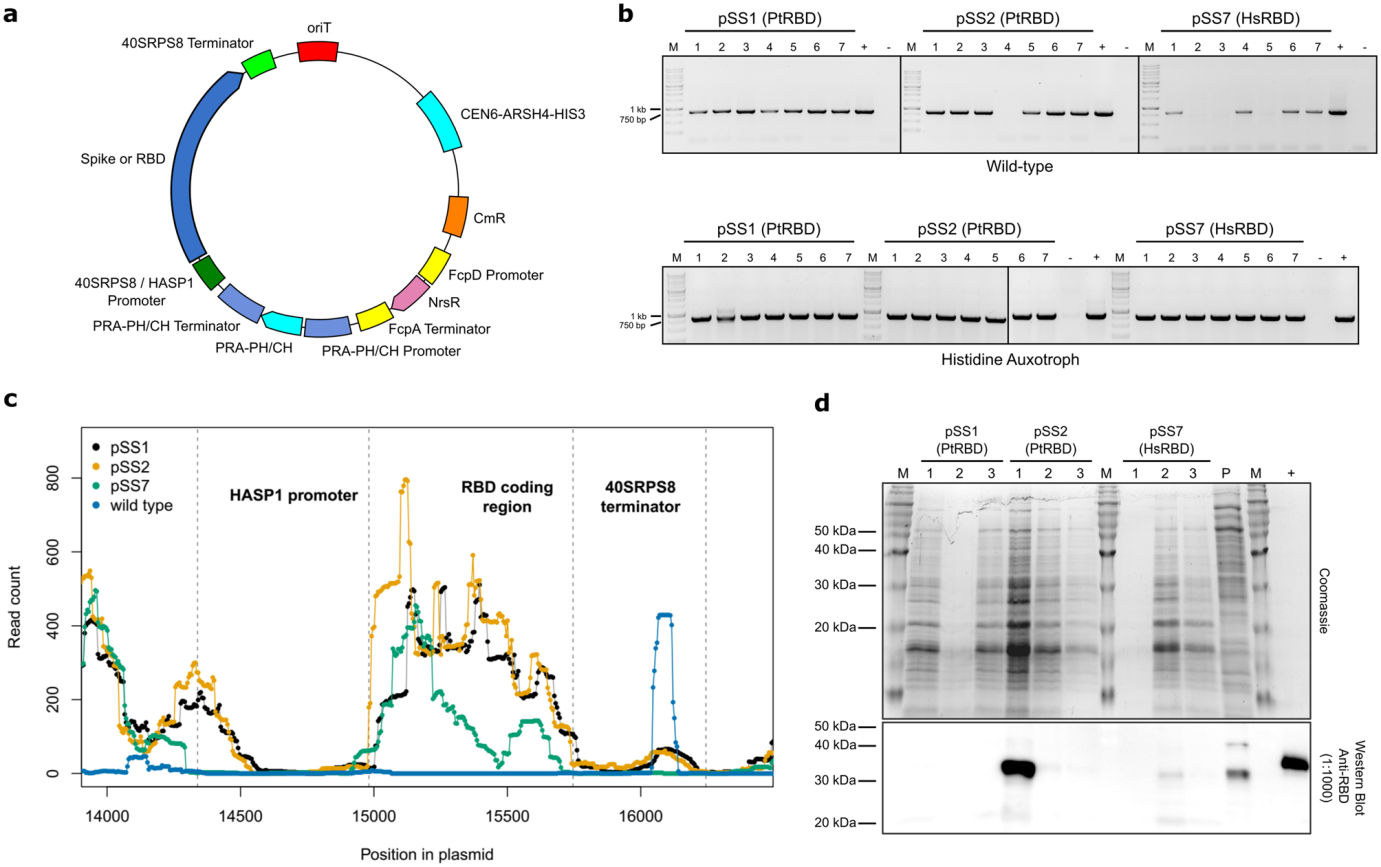
Expression of SARS-CoV-2 RBD from stably maintained plasmids in *P. tricornutum*. (**a**) Schematic of expression plasmids used in this study. oriT, conjugative origin of transfer; CEN6-ARSH4-HIS3, yeast replication origin and selective marker; CmR, chloramphenicol resistance gene; FcpD, promoter and terminator elements derived from the FcpD gene^33^; NrsR, nourseothricin resistance gene; PRA-PH/CH, promoter, coding region and terminator from the PRA-PH/CH gene (histidine biosynthesis); 40SRPS8/HASP1 promoter, promoter elements from the 40SRPS8/HASP1 promoter; 40SRPS8 terminator, terminator derived from the 40SRPS8 gene. (**b**) Stability of the RBD coding region assessed using PCR with RBD-specific primers in plasmids from wild-type (top) or histidine auxotroph (bottom) *P. tricornutum* clones. Numbers indicate individual plasmids isolated from *P. tricornutum* clones. The expected product is 833 bp. PtRBD, codon-optimized for *P. tricornutum*; HsRBD, codon-optimized for HEK293 cells; M, 1-kb DNA ladder. (**c**) Plot of RNAseq reads mapped to the pSS1 plasmid sequence for wild-type *P. tricornutum* (WT) and *P. tricornutum* harbouring pSS1, pSS2, or pSS7. (**d**) Coomassie-stained gel (top) and western blot (bottom) of whole cell lysates from 3 histidine auxotroph clones harbouring pSS1, pSS2 or pSS7, and from a pool of clones harbouring pSS1 (P). M, PiNK Plus Prestained Protein Ladder. +, 5 ng of commercially available RBD purified from HEK293 cells. Uncropped gel images for panels (**c**) and (**d**) are shown in Supplementary Figures S9–S13.

The coding region for the RBD of the SARS-CoV-2 spike protein with an added C-terminal 6X-histidine tag and TEV protease site was cloned into the *E. coli-S. cerevisiae-P. tricornutum* plasmid vector pPtGE31 (Fig. 1a, Supplementary Tables S1, S2). In the first set of plasmids (pSS1 and pSS2), the RBD coding region was codon optimized for *P. tricornutum* (PtRBD) and targeted for secretion using the promoter and secretory signal from the *P. tricornutum* *HASP1* gene (highly abundant secreted protein 1)^40^. pSS1 and pSS2 differ in nucleotide polymorphisms in the promoter that are present in different alleles of *HASP1*^41^ (Supplementary Table S1). Another plasmid (pSS7) used a human codon optimized RBD coding region (HsRBD) with the SARS-CoV-2 spike protein secretory signal (Supplementary Table 2) driven by the 40SRPS8 promoter^33^. Plasmids were introduced into wild-type *P. tricornutum* or a histidine auxotroph strain^42^ from *E. coli* by conjugation^19^. After isolation of single *P. tricornutum* clones, retention of the RBD coding region was assessed after 28 days growth in liquid culture. As shown in Fig. 1b, diagnostic PCR on individual clones for the PtRBD coding region was positive for 7/7 clones of pSS1, 6/7 clones of pSS2, and 4/7 clones of pSS7 in the wild-type strain. In contrast, the RBD coding region was detected in all clones examined in the histidine auxotroph strain of *P. tricornutum*. Transcription of the RBD was confirmed by RNAseq for the wild-type strain harbouring pSS1, pSS2 and pSS7, but no reads for the RBD coding region were evident from RNAseq of the wild-type strain without plasmid (Fig. 1c, Supplementary Figure S1). Reads for the *HASP1* promoter and *40SRPS8* terminator elements were evident in all strains; in the wild-type strain, these reads are solely derived from the chromosomal copies of the regulatory elements and mapped to the corresponding elements on the plasmids.

To identify strains that exhibited growth as well as expression of the RBD, three clones each of a histidine auxotroph strain harbouring pSS1 (PtRBD), pSS2 (PtRBD) or pSS7 (HsRBD) plasmids were expanded in liquid culture and RBD expression was examined by western blots using a polyclonal anti-RBD antibody (Fig. 1d). The strains exhibited different growths, and only one clone harbouring pSS2 revealed robust RBD expression (pSS2-1) (Fig. 1d). We also performed a preliminary metal-affinity purification of the 6x-histidine tagged RBD using a whole-cell extract from a pooled culture of pSS1 clones (Fig. 1d, lane P). Western blotting of this pooled whole-cell extract revealed a reactive band of the same size as observed in the pSS2 clone (pSS2-1), both of which were slightly smaller than the positive control RBD purified from HEK293 cells. One expressing clone, pSS2-1, was chosen for long-term growth and expression experiments by serial passaging of pSS2-1 cultures into fresh L1 media as described in the methods. At 7 months, larger-scale photobioreactors (20 L) were seeded with the pSS2-1 strain, and samples were taken to first assess RBD expression by western blotting. Two photobioreactors (B2 and C6) showed RBD expression, whereas weaker levels of RBD expression were observed in the other 12 photobioreactors (Supplementary Figure S2). We then performed diagnostic PCR for the RBD coding region on samples taken from the remaining 12 photobioreactors, and found evidence of the RBD in 10 of these, indicating that low RBD expression was not due to loss of the RBD coding region (Supplementary Figure S2).

We also constructed *HASP1*-regulated RBD plasmids with glutathione S-transferase (GST), 10X-histidine, and IgG1-Fc purification tags as N- or C-terminal fusions that were transformed into wild-type *P. tricornutum* to determine stability and expression levels in small-scale laboratory cultures. In each case, western blotting with a polyclonal anti-RBD antibody revealed expression of the RBD fusions, although at varying levels (Supplementary Figure S3). Together, these data show that plasmids with RBD coding regions optimized for expression in *P. tricornutum* can be maintained in laboratory or larger scale cultures for at least 7 months and that RBD expression can be detected both by RNAseq and by a polyclonal anti-RBD antibody. Moreover, 4 different purification tags are compatible with RBD expression in *P. tricornutum*, providing different strategies for purification.

**Figure 2:**
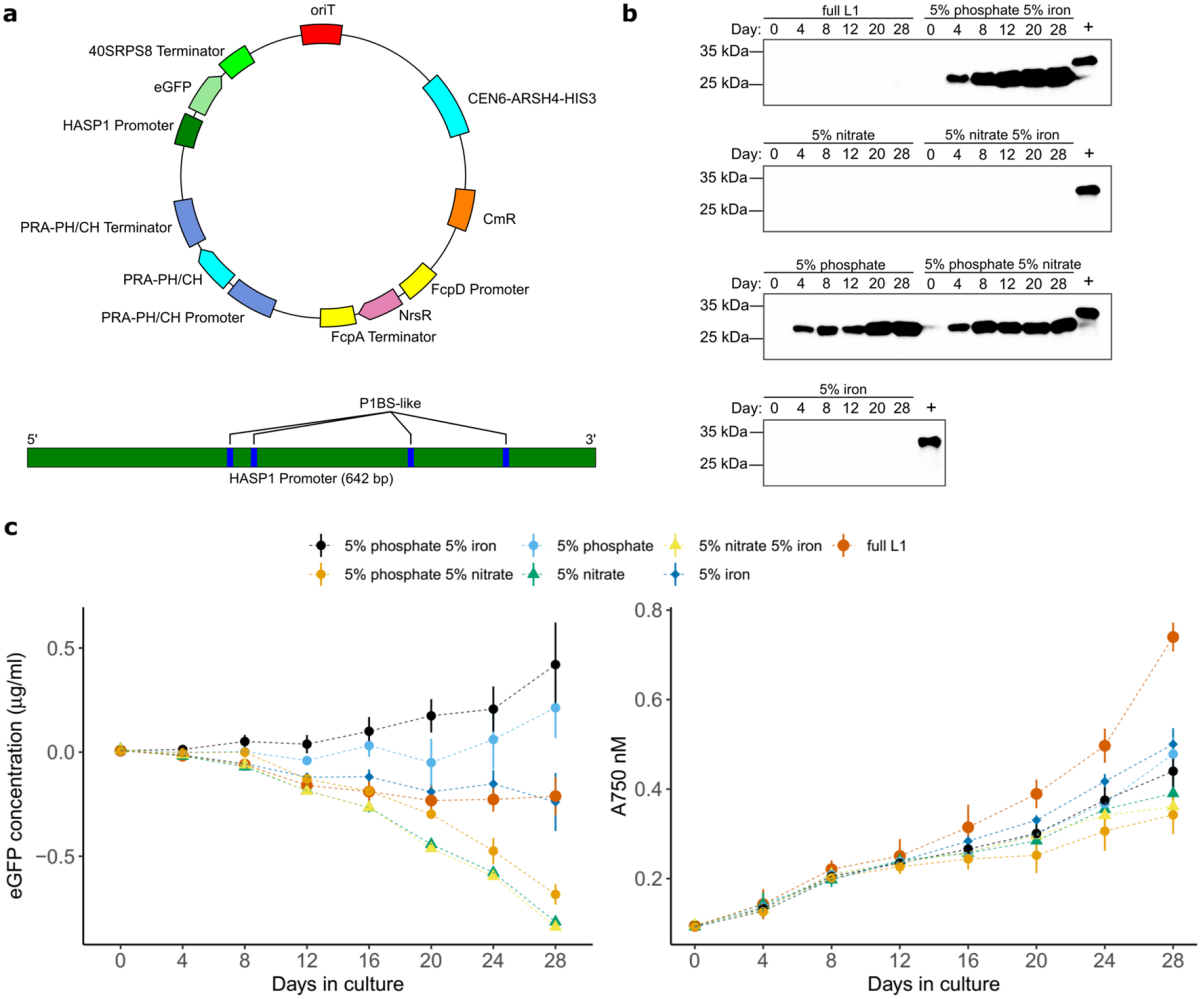
Phosphate-regulated expression of eGFP from the *HASP1* promoter. (**a**) Schematic of the plasmid construct (top) and putative phosphate-regulatory motifs (P1BS-like) in the *HASP1* promoter region (bottom). (**b**) Western blot with an anti-GFP antibody of whole cell extracts from pSS10 clone 3 grown in the different media conditions and sampled on the indicated days. The (+) control lane is a purchased 6X-histidine tagged GFP. eGFP expressed in our experiments is not 6X-histidine tagged. (**c**) Left panel, plot of eGFP concentration in culture supernatants measured over time under different media conditions for one clone of pSS10 (clone 3). Supernatant fluorescence values were subtracted from values taken from supernatants of wild-type *P. tricornutum* grown in parallel to correct for autofluorescence. Right panel, plot of absorbance at 750 nM over time for pSS10 grown in the indicated media. For both plots, data points are the mean of two replicate experiments each with three technical replicates. The whiskers represent the standard deviation. Uncropped gel images for panel (**b**) are shown in Supplementary Figures S14 and S15.

**Figure 3:**
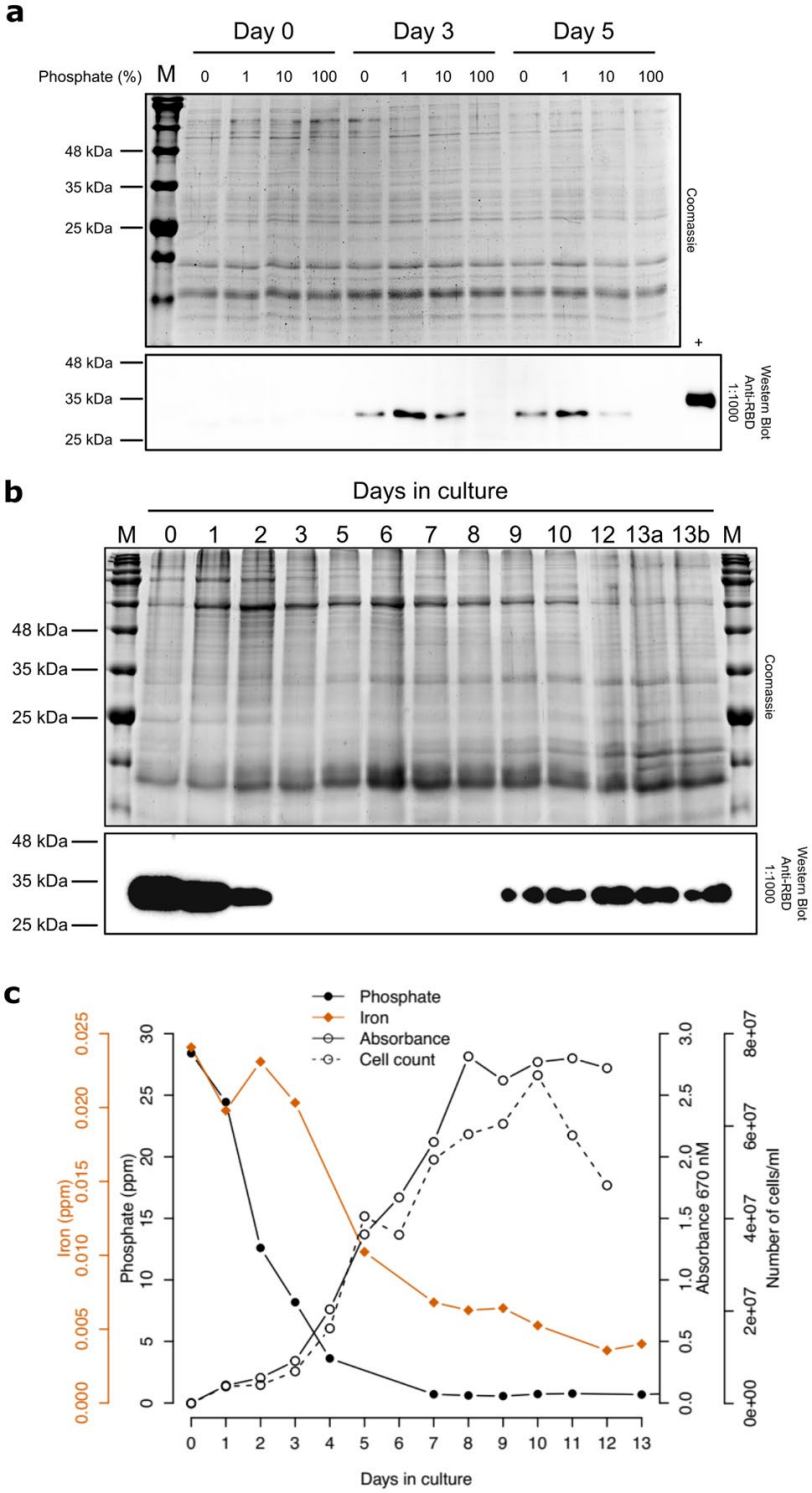
Phosphate-regulated expression of algae codon-optimized RBD from the *HASP1* promoter. (**a**) RBD expression in response to varying phosphate concentrations measured over time. Shown is a coomassie-stained gel (top) of whole cell extracts from cultures harbouring pSS2 at day 0, 3 and 5 post inoculation into media with 0%, 1%, 10% or 100% of phosphate (Supplementary Table 3). RBD expression was assessed by western blotting (bottom) using a polyclonal anti-RBD antibody. The positive control (+) is 5 ng of commercially available RBD purified from HEK293 cells. M, BLUelf Prestained Protein Ladder. (**b**) RBD expression in a 5 L bioreactor. The top image is a coomassie-stained gel of whole cell lysates sampled at the indicated day, while the bottom image is a western blot using a polyclonal anti-RBD antibody. M, BLUelf Prestained Protein Ladder. (**c**) Plot of phosphate (filled circles) and iron (filled diamonds) as a function of growth over (open circles, solid line) and cell number (open circles, dashed line) over time in a photobioreactor. Phosphate and iron are plotted as parts per million (ppm), growth is plotted as absorbance readings at A670 nM, and cell number as cell count per mL of culture. Uncropped gel images for panels (**a**) and (**b**) are shown in Supplementary Figures S16–S19.

### Limiting phosphate induces expression from the *HASP1* promoter

In an effort to maximize production of the RBD, we noted that the HASP1 protein contained a predicted phytase domain with putative phosphatase activity and that putative phosphate-regulatory motifs were identified in the *HASP1* promoter sequence (Fig. 2a)^40^. These observations, along with previous phosphate-regulation studies^43^, suggested that the *HASP1* promoter may be responsive to low phosphate media levels. We mapped RNAseq reads from a previous transcriptomic study that examined global gene expression changes in response to reduced media phosphate^44^ to our recently completed telomere-to-telomere genome assembly of the *P. tricornutum* strain used in this study^41^. The *HASP1* gene was significantly up-regulated in media depleted of phosphate, and then down-regulated when phosphate was replenished (Supplementary Figure S4). We routinely culture *P. tricornutum* in an L1 media formulation that contains ten times the phosphate than used in other media formulations ($3.62 \times 10^{-4}$ M versus $3.62 \times 10^{-5}$ M, Supplementary Table S3). This media condition yields much higher cell densities, but would likely suppress transcription from the *HASP1* promoter. Collectively, these observations suggested that the *HASP1* promoter in a plasmid-based context could be regulated by limiting phosphate levels in the *P. tricornutum* growth media, thus providing a regulated, inducible system for expression of the RBD.

We tested this hypothesis by first constructing a plasmid (pSS10) where the coding region for enhanced green fluorescent protein (eGFP) was cloned downstream of a 642-bp sequence containing the *HASP1* promoter region (Fig. 2a). Wild-type *P. tricornutum* clones harbouring pSS10 were selected, grown in media containing high concentrations of phosphate (100%, 362 $\upmu$M, or $\sim$ 34 ppm) and then diluted into test tubes containing media with phosphate at 5% of normal media levels (18 $\upmu$M, $\sim$ 1.5ppm), or with reduced amounts of other media constituents (a comparison of media formulations is found in Supplementary Table S3). We specifically focused on phosphate, nitrate, and iron as these are critical for growth of algae in natural environments and previous transcriptomic studies of *P. tricornutum* revealed significant global gene expression changes in response to reduction of these components in media^45–47^. We examined eGFP expression by western blots of whole cell lysates of cultures sampled at different days post inoculation and observed robust expression of eGFP in phosphate-depleted media as early as day 4 in the time course (Fig. 2b). No eGFP expression was visible in strains grown in media depleted of nitrate or iron (Fig. 2b). The *HASP1-eGFP* induction by low phosphate media was replicated with an independent isolate of pSS10 in *P. tricornutum* (Supplementary Figure S5). Secreted eGFP was evident after 8–12 days in supernatants of media with 5% phosphate and 5% iron, and continued to increase over the time course of the experiment (Fig. 2c, left panel). In contrast, no secreted eGFP was observed in supernatants of L1 media (Full L1). Limiting phosphate in combination with nitrate, nitrate alone, iron alone, or nitrate in combination with iron, did not stimulate eGFP secretion to the supernatant. Differences in eGFP secretion did not appear linked to effects of media on growth of *P. tricornutum* (Fig. 2c, right panel). eGFP secretion to the culture supernatant was observed with 5 other pSS10 clones in 5% phosphate 5% iron media, but not in modified L1 media (Supplementary Figure S5). Whole plasmid sequencing of the six pSS10 clones revealed differences relative to the predicted sequence that are consistent with mutations occurring during conjugation from *E. coli* to *P. tricornutum* (Supplementary Table S4)^19^. Collectively, these data show that eGFP secretion was robust in media with reduced phosphate and iron, whereas limiting phosphate alone was sufficient to stimulate eGFP expression from the *HASP1* promoter.

### Phosphate-regulated expression of the RBD

Encouraged by this result, we next tested whether expression of the RBD from the *HASP1* promoter was also regulated by limiting phosphate and/or iron. We first tested this by diluting a stationary phase culture ($\sim 7\times 10^{7}$ cells $\hbox {mL}^{-1}$) of the pSS2-1 strain into small-scale cultures (10 mL) with fresh L1 media containing different concentrations of added phosphate (0%, 1%, 10% and 100% relative to our modified L1 media, Supplementary Table 3). By day 3, RBD expression as monitored by western blotting of whole cell extracts was observed in all cultures except those containing 100% phosphate (Fig. 3a), indicating that the *HASP1* promoter was responsive to reduced levels of phosphate with maximal induction between 1 and 10% phosphate ($\sim$ 0.34 to $\sim$ 3.4 ppm).

We next scaled expression to 5 L to facilitate monitoring of growth rate, RBD expression, and phosphate and iron concentrations in a photobioreactor containing our modified L1 media ($\sim$ 34 ppm phosphate). For this experiment, we seeded the photobioreactor with 500 mL of a culture that was grown to $\sim 2\times 10^{7}$ cells $\hbox {mL}^{-1}$ that showed high levels of RBD expression (Fig. 3b, lane 0). RBD levels as indicated by western blots of whole cell lysates were high at early time points (days 1 and 2), repressed at days 3–8, and then visibly induced by day 9. Reduction of phosphate in the media to $\sim$ 0.5 ppm correlated with maximal RBD expression (Fig. 3c). A similar reduction in iron was also observed (Fig. 3c). Taken together, these data show that the *HASP1* promoter can be induced or repressed based on phosphate levels in the culture media, with < 0.5 ppm phosphate inducing expression. This result also explains why we found that in our modified L1 media, which contains $\sim$ 34 ppm phosphate or 10 times more than other studies^40^ (Supplementary Table S3), *HASP1*-driven expression of the RBD was highest in late stationary phase when phosphate depletion to levels necessary for induction occurred, and why our western blot data shows full RBD repression.

Our initial protein purification strategy was based on secretion of the expressed SARS-CoV-2 proteins into algae culture supernatant using the *HASP1* secretory signal and with the endoplasmic retention signal (RRAR) of the full-length Spike glycoprotein intact or mutated to alanine residues. However, we found no evidence of secretion of the expressed RBD in culture supernatants, either in small scale cultures or larger bioreactors. Lack of RBD secretion was not due to the *HASP1* promoter and secretory peptide, as we observed robust secretion of the *HASP1-eGFP* construct (Fig. 2b). We also found no evidence of RBD secretion in culture supernatants of strains harbouring pSS7 that expresses a human codon-optimized RBD driven by the *P. tricornutum* 40SRPS8 constitutive promoter and the native SARS-CoV-2 spike protein secretion signal. Moreover, plasmids expressing algae codon-optimized versions of the full-length spike protein (pSS3–pSS6) also had poor expression levels and we found significant proteolysis of the spike protein as evidenced by mass spectrometry analyses of concentrated cell-free media (Supplementary Figure S6). It is not clear, however, if the observed peptides were due to proteolysis after secretion or from intracellular proteolysis and subsequent release to the supernatant from lysed cells.

**Figure 4:**
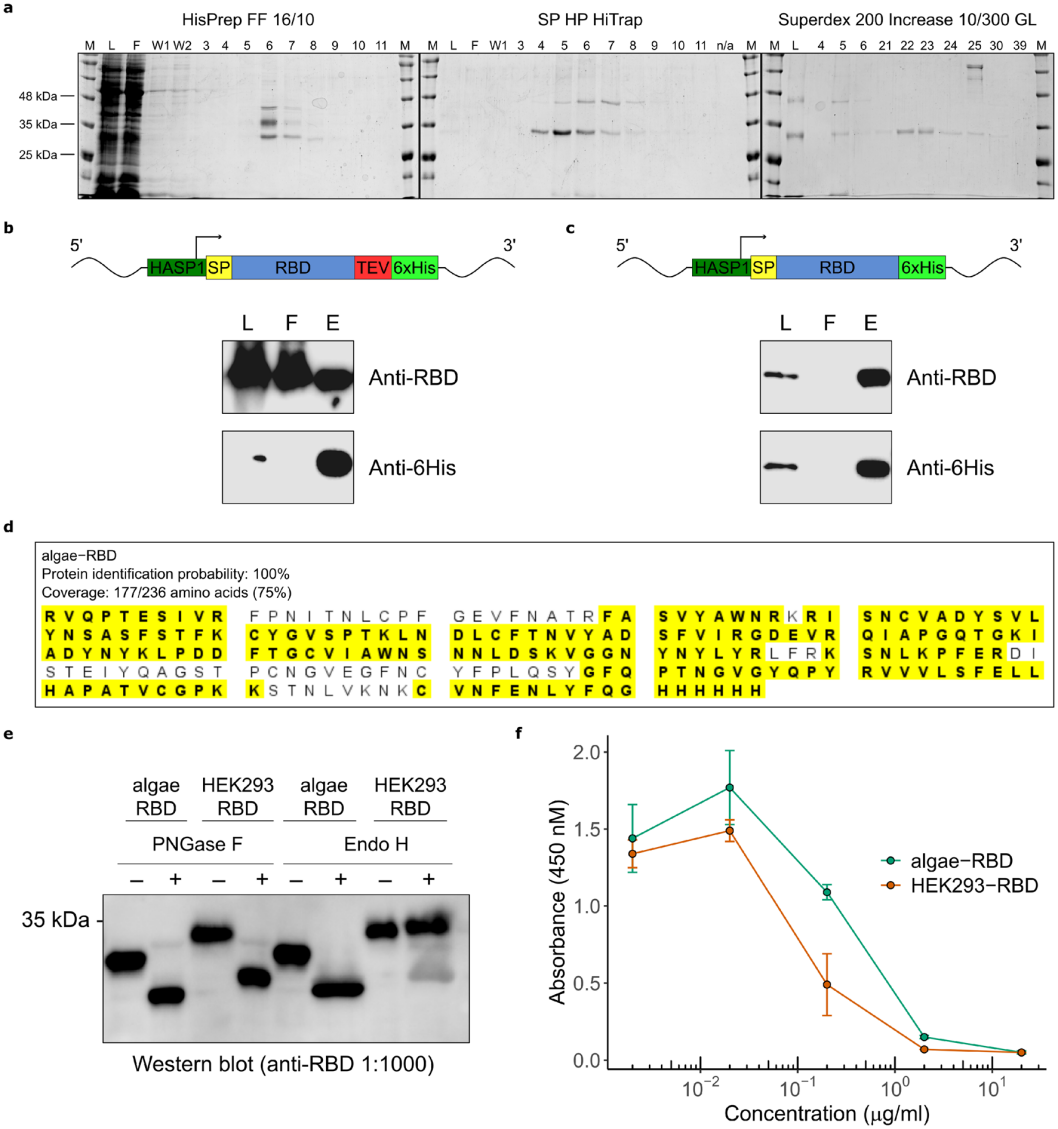
Purification and activity of the algae-RBD. (**a**) Representative gel images of individual chromatographic steps in the purification, starting with the HisPrep FF column on the left. For each step, numbers above the gel indicate elution fractions. M, BLUelf Prestained Protein Ladder. (**b**) Western blots with an anti-RBD or anti-6XHis antibody of the load (L), flow through (FT) and elution fraction 6 (E) from the HisPrep FF column purification of the algae-RBD construct with a TEV protease site. (**c**) Western blots as in panel (**b**) of a purification of an algae-RBD construct lacking the TEV protease sie. (**d**) Protein identification of the algae-RBD by MALDI MS. The amino acid sequence of the algae-RBD is shown and peptides identified are highlighted in yellow. (**e**) Endoglycosidase digestion assays to detect *N*-linked glycosylation. Purified algae-RBD or HEK293-RBD were incubated with (+) or without (−) the indicated glycosylase and visualized by western blotting with a polyclonal anti-RBD antibody. (**f**) ACE2 inhibition binding assay. Shown is a plot of absorbance readings of ACE2-bound Fc-tagged RBD at various concentrations of algae-RBD or commercially available RBD made in HEK293 cells (HEK293-RBD). Data are three independent experimental replicates with error bars representing standard deviation of the mean. Uncropped gel images for panels (**a**), (**b**), (**c**), and (**e**) are shown in Supplementary Figures S20–S27.

### A genetically encoded TEV protease site is associated with loss of C-terminal histidine tag from expressed algae-RBD

We focused on purifying the 6X-histidine tagged RBD from whole-cell extracts of *P. tricornutum* using a combination of metal affinity, ion exchange and gel filtration chromatography (Fig. 4a). From 5 L photobioreactor runs, we achieved RBD yields of 28–34 $\upmu$g of RBD. During repeated purification runs, we noted that a significant portion of the expressed RBD was present in the column flow through ($\sim$ 90 to 95%) (Fig. 4b). Purification under denaturing conditions (6 M urea) did not improve binding to the metal affinity column (Supplementary Figure S7). Interestingly, western blotting of the column load, flow through and eluate with anti-6XHis and anti-RBD antibodies showed that the majority of the RBD present in the load and flow through lacked a 6X-histidine tag, explaining why most of the expressed RBD did not bind the metal affinity column. We considered the possibility that the TEV protease site in the algae-RBD construct was a potential substrate for a *P. tricornutum* protease, thus causing loss of the C-terminal 6X-histidine tag from expressed algae-RBD. When we deleted the TEV protease site from the construct (pSS72), but retained the C-terminal 6X-histidine tag, the majority of the protein was present in the eluate and not in the flow-through (Fig. 4C), suggesting that the presence of the TEV protease site caused loss of the 6X-histidine tag. The identity of the purified algae-RBD was confirmed by mass spectrometry protein identification (Fig. 4d) and by western blotting with a polyclonal anti-RBD (Fig. 4e). We noted again that the apparent molecular weight of the algae-RBD was lower than that of RBD produced in mammalian cell lines (HEK293) (Fig. 4e).

### Algae-RBD is biologically active

We tested purified algae-RBD for the presence of *N*-linked glycosylated residues by treatment with the endoglycosidases PNGase F and Endo H. Endo H has specificity for mannose *N*-linked modifications whereas PNGase F will cleave mannose, hybrid and complex *N*-linked modifications. As shown in Fig. 4e, treatment with PNGase F and Endo H reduced the apparent size of the algae-RBD. A similar treatment of commercially available RBD purified from mammalian cell lines (HEK293-RBD) also reduced the apparent size of the RBD (Fig. 4e). The algae-RBD and HEK293-RBD do not differ in primary sequence of the recombinant constructs and thus the slight difference in the apparent size of the pre- and post-treated algae-RBD versus HEK293-RBD could be due to post-translational modifications other than *N*-linked glycosylation, including recently reported *O*-linked glycosylation of the SARS-CoV-2 spike protein^48^. These data, however, are consistent with both RBD preparations containing mannose-rich *N*-linked glycosylated residues.

The biological activity of the algae-RBD was confirmed by performing an in vitro assay where addition of RBD was used to competitively inhibit binding of an Fc-tagged mammalian purified RBD to an immobilized ACE2 extracellular domain (Fig. 4f). For this experiment, we used both the algae-RBD and a commercially available HEK293-RBD as inhibitors. We found very similar inhibition profiles for both the algae-RBD and HEK293-RBD, with 50% binding inhibition at 0.656 $\upmu$g $\hbox {mL}^{-1}$ and 0.153 $\upmu$g $\hbox {mL}^{-1}$, respectively. Together, these data show that biologically active algae-RBD that contains *N*-linked glycosylations can be purified from whole cell extracts of *P. tricornutum*.

### Algae-RBD is serologically active in a LFA device

**Figure 5:**
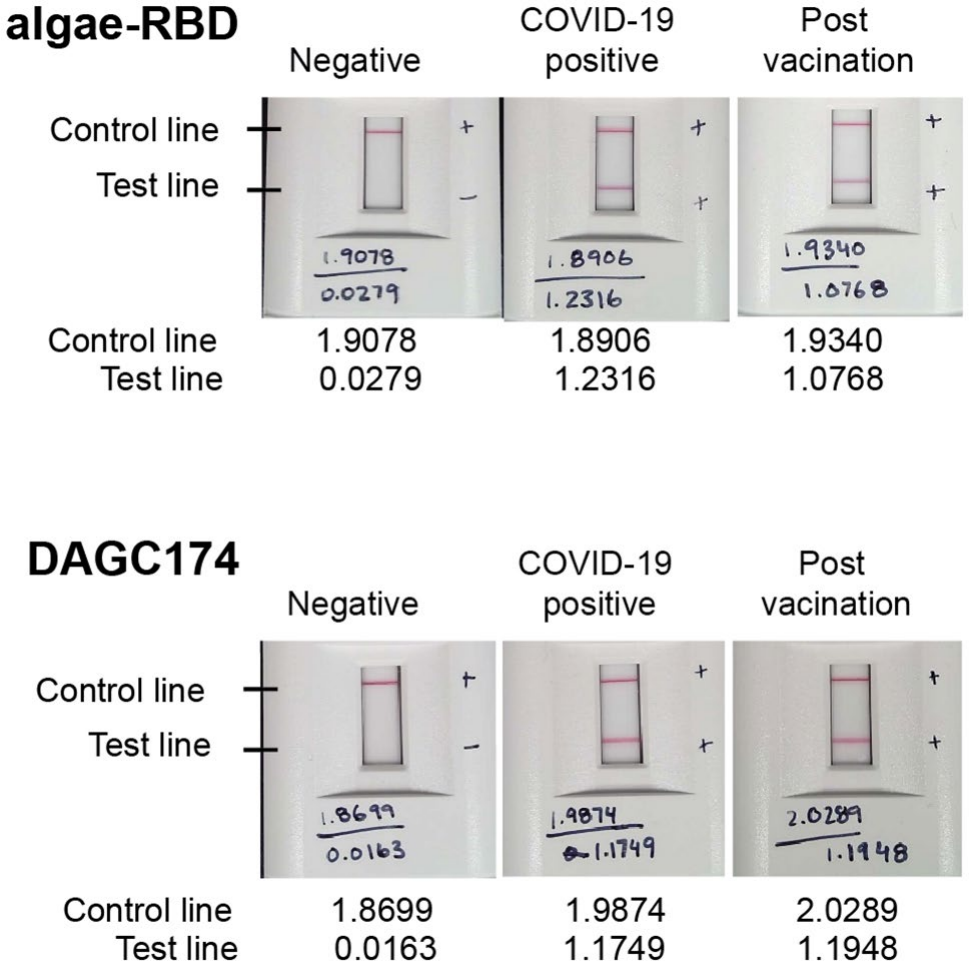
Algae-RBD is serologically active in an LFA device. Representative images of LFA devices with the algae-RBD (top) or DAGC174 RBD (bottom) as the capture antigen. Each test strip has two reaction lines, the control line that detects IgG antibodies, and the test line that specifically captures anti-RBD IgG antibodies. Values reported on each device are optical density readings of the control or test strip. Negative, serum confirmed SARS-CoV-2 negative by PCR; COVID-19 positive, serum confirmed SARS-CoV-2 positive by PCR; Post vaccination, serum post-vaccination with two doses of Pfizer-BioNTech vaccine. Additional data is presented in Supplementary Table 4.

To demonstrate the practical application of the algae-RBD in a serological test that would commonly be used to determine immune response to SARS-CoV-2 infection, or immune response post-vaccination, we conjugated the algae-RBD to gold beads that were applied to a lateral flow assay (LFA) device. As shown in Fig. 5 and Supplementary Table S5, the algae-RBD LFA was able to detect the presence of anti-RBD IgG antibodies in serum from two sources; from patients previously infected with SARS-CoV-2 as determined by PCR, and from patients confirmed COVID-19 negative by PCR and subsequently immunized with two doses of the Pfizer-BioNTech COVID-19 vaccine. Importantly, the sensitivity of the LFA with the algae-RBD was equivalent to the LFA with the commercially available DAGC174 RBD antigen. No reactivity was observed with either antigen when tested against serum negative for COVID-19 by PCR testing. Taken together, these data shows that the algae-RBD is bioequivalent to RBD made in mammalian cells when used in a LFA device.

## Discussion

Available data and modelling indicate that the current COVID-19 pandemic will remain a public health issue beyond the current waves of SARS-CoV-2 infection^49^. Moreover, SARS-CoV-2 variants that have enhanced infectivity and/or pathogenicity relative to the parental SARS-CoV-2 strain, such as the current Omicron B.1.1.529 and BA.2 variants, will likely continue to arise^50^, and it is possible these and other variants will become endemic. Rapid LFAs that utilize recombinantly expressed SARS-CoV-2 antigens are one type of serological test useful for viral exposure monitoring or for determining immune response post-vaccination^51^. Widespread use of LFAs will require a scalable source of immunologically reactive viral antigen. Here, we show that the marine diatom *P. tricornutum* is a viable orthogonal and scalable system for overexpression and purification of the SARS-CoV-2 RBD, and possibly other viral antigens, for use in pandemic diagnostics. In particular, the minimal biocontainment measures, defined growth media, paucity of immunologically cross-reactive proteins, and lack of infectivity by mammalian viruses make *P. tricornutum* an attractive orthologonal system.

Our proof-of-principle study focused on expressing the RBD of the SARS-CoV-2 spike protein in algae using plasmid-based expression systems that allowed us to test a number promoter-RBD combinations, one of which was the *HASP1* promoter. We found that the *HASP1* promoter in a plasmid-based context was responsive to limiting phosphate levels in the culture media, with expression induced at phosphate levels below 0.5 ppm. While our study measured media phosphate levels rather than total phosphate (cells plus media)^44^, our observations agree well with past transcriptomic studies that measured global *P. tricornutum* gene expression changes in response to limiting phosphate conditions^44,52^. Interestingly, we found that low iron (5% of normal) in addition to low phosphate promoted maximal secretion of eGFP into culture media with the *HASP1-eGFP* constructs. Previous studies on *HASP1* regulation or phosphate responsiveness did not focus on iron and it is tempting to speculate that low iron levels, in combination with low phosphate, stimulate a general increase in expression of secretory pathway components as a mechanism for scavenging iron. Indeed, our data suggest that addition of excess phosphate to media is a viable strategy to stringently repress expression from the *HASP1* promoter that would be applicable for toxic proteins or for timing expression to particular growth stages. Conversely, growth in media depleted of phosphate and iron would induce expression and possible secretion, thus adding to the genetic toolbox of inducible promoters in *P. tricornutum* that is currently based on the nitrate reductase promoter^26,27,53^ and alkaline phosphatase promoter^28^.

As a protein expression system, *P. tricornutum* has many advantages, including an *N*-linked glycosylation pathway that is very similar to that found in mammals. Indeed, this similarity may be one reason why the algae-RBD is bioequivalent to the RBD made in mammalian systems in the LFA serological test device. However, the molecular tools available for protein expression in *P. tricornutum* are still under development. For instance, we found that the HASP1 secretory peptide functioned when fused to eGFP, but not when fused to the RBD. This finding parallels that found in another algae, *Chlamydomonas*, where expressed RBD was retained in the ER/golgi complex and total yields of partially pure RBD were similar to ours^12^. The RBD that we purified from whole cell extracts was essentially homogeneous as evidenced by a single discernible band on an SDS-PAGE gel and possessed *N*-linked glycosylation. This finding suggests that a portion of expressed RBD is transited through the ER/golgi apparatus and is either secreted at levels too low to be detected in culture supernatants, or is not secreted at all. We do not know why the RBD is recalcitrant to secretion when overexpressed. It is possible that other secretory peptides are better optimized for RBD expression and secretion, and a screen for promoter/signal peptides that robustly secrete the RBD is underway.

An important consideration for any orthogonal protein overexpression systems is yield. We chose to pursue a 6X-histidine tag purification strategy based on its widespread use in the community, availability of resources, and cost relative to other purification strategies. One interesting observation from our purification attempts was that 90–95% of expressed RBD did not bind the metal-affinity column in the first chromatography step. Surprisingly, we found that the majority of expressed RBD was lacking all, or a portion of, the C-terminal 6X-histidine tag. Deleting the TEV protease site from the construct resulted in a substantial increase in yield with no protein visible by western blotting in the metal-affinity column flow-through. The simplest interpretation of this result is that *P. tricornutum* encodes a protease that recognizes the TEV protease site and subsequent cleavage releases the C-terminal 6X-histidine tag. The C-terminal 6X-histidine tag could also be lost by pre-mature transcription termination, post-transcriptional processing or pre-mature translation termination. The fact that we can recover some expressed algae-RBD-6X-His containing the TEV protease site suggests that, regardless of the mechanism that removes the C-terminal 6X-histidine tag, it is inefficient or restricted to a particular sub-cellular compartment (the ER or golgi). It is not clear from other studies with *P. tricornutum* if similar issues were observed during purification of histidine-tagged proteins^54^, although proteolytic removal of C-terminal tags from overexpressed proteins has been observed in *E. coli*^55^. This data also suggest that different tags may be better suited for expression and purification of the algae-RBD (Supplementary Figure S3).

Crucially, the algae-RBD was bioequivalent to RBD made in mammalian expression systems in an ACE2 binding assay and a LFA device used for serological testing. This result highlights the potential of *P. tricornutum* as a viable orthogonal protein expression system for SARS-CoV-2 antigens. *P. tricornutum*, and other algae, have a number of advantages over other expression systems, foremost being minimal biocontainment requirements, a defined growth media that lacks potentially cross-reactive antigens present in serum needed for mammalian cell culturing, insensitivity to infection by mammalian viruses, and scalability (> 10,000 L). Moreover, the *P. tricornutum* histidine auxotroph^42^ that we used in our experiments alleviates the need for antibiotic selection of plasmids, further reducing the complexity and cost of growth media. An additional advantage of plasmid-based expression systems is the ability to screen through large numbers of potential expression constructs in response to emerging pandemic viral threats. Indeed, we are currently pursuing strategies to express RBDs with variant mutations and other proteins for basic science and diagnostic applications.

## Methods

### Microbial strains and growth conditions

*Saccharomyces cerevisiae* VL6-48 (ATCC MYA-3666: *MAT*$\alpha$ *his3*-$\Delta$200 *trp1*-$\Delta$1 *ura3-52* *lys2 ade2-1 met14* *cir* $^{0}$) was grown in YPD medium or complete minimal medium lacking histidine (Teknova) supplemented with 60 mg $\hbox {L}^{-1}$ adenine sulfate. Complete minimal media used for spheroplast transformation contained 1 M sorbitol. *E. coli* (Epi300, Epicentre) was grown in Luria Broth (LB) supplemented with appropriate antibiotics (chloramphenicol (25 mg $\hbox {L}^{-1}$) or ampicillin (50 mg $\hbox {L}^{-1}$) or gentamicin (20 mg $\hbox {l}^{-1}$)). *P. tricornutum* (Culture Collection of Algae and Protozoa CCAP 1055/1) or the *P. tricornutum* histidine auxotroph^42^ were grown in L1 medium without silica, with or without histidine (200 $\hbox {mg\,L}^{-1}$), supplemented with appropriate antibiotics (Zeocin (50 mg $\hbox {L}^{-1}$) or nourseothricin (150 mg $\hbox {L}^{-1}$)), at 18$^{\circ }$C under cool white fluorescent lights (75 $\upmu$E $\hbox {m}^{-2} \hbox {s}^{-1}$) and a photoperiod of 16 h light:8 h dark. L1 media supplemented with nourseothricin contained half the normal amount of aquil salts. Long-term cultures of *P. tricornutum* with RBD expression plasmids were grown in L1 media supplemented with nourseothricin (for the wild-type strain) or with no added antibiotic (for the histidine auxotroph strain) to a density of 20 million cells $\hbox {mL}^{-1}$ and passaged by diluting 1:10 into fresh L1 media. Cultures were examined for contamination by visual light microscopy (Supplementary Figure S8).

### Plasmid design and construction

All plasmids (Supplementary Table 1) were constructed using a modified yeast assembly protocol^56,57^. We cloned versions of the SARS-CoV-2 spike protein gene into the *E. coli*/*P. tricornutum* shuttle plasmid pPtGE31^33^. Primers are listed in Supplementary Table 5. We obtained SARS-CoV-2 expression plasmids (Krammer Lab, Icahn School of Medicine, Mount Sinai) that served as templates for PCR amplification of the human codon-optimized spike and receptor-binding domain coding regions for cloning into *P. tricornutum* expression plasmids. We also ordered synthetic constructs corresponding to the full-length spike protein gene and RBD from IDT-DNA that were codon-optimized for *P. tricornutum*. The nine initial constructs are listed in Supplementary Table 1 and a representative schematic can be seen in Fig. 1a. The constructs differed in the following ways: codon-optimization for human or *P. tricornutum*, full-length or RBD of the spike protein, version 1 or version 2 of the *P. tricornutum* *HASP1* promoter (originating from two homologous *P. tricornutum* chromosomes, NBCI accession number XM_002181840.1, UNIPROT accession number PHATRDRAFT_47612). We also cloned a version of the full-length construct with two proline stabilizing mutations and a mutation of the furin cleavage site (RRAR) to an alanine, in addition to a stabilizing trimerization motif. The constructs were made with a promoter from the *P. tricornutum* *40SRPS8* (40S ribosomal protein S8) gene or from the *HASP1* gene. All constructs contained the *40SRPS8* terminator downstream of the spike or RBD coding sequence. All constructs also included a histidine marker (PRA-PH/CH) expression cassette from the pPtPRAPHCH plasmid^42^ for selection and maintenance in a *P. tricornutum* histidine auxotroph strain. Plasmids encoding the *P. tricornutum* codon-optimized versions had the *HASP1* promoter and the HASP1 secretory signal peptide, while plasmids encoding the human codon-optimized versions used the *40SRPS8* promoter and the native spike protein secretory signal. The plasmid constructs were assembled in yeast by co-transforming linear DNA fragments of the pPtGE31 plasmid backbone, the PRA-PH/CH expression cassette, the *HASP1* or *40SRPS8* promoter, and the spike or RBD protein gene. Resultant yeast colonies were pooled, DNA extracted, and transformed into *E. coli*. Single *E. coli* colonies were grown and plasmid DNA isolated using standard plasmid mini-prep kits. Correct assembly was confirmed by restriction enzyme digests, by whole-plasmid sequencing using a MinION sequencer from Oxford Nanopore Technologies, and by Sanger sequencing of the spike and RBD expression cassettes at the London Regional Genomics Centre.

### Plasmid validation

Plasmid DNA was extracted using the NEB miniprep kit (T1010L) and 400 ng of DNA was used as input for the rapid barcoding kit library prep (SQK-RBK004). Plasmids were then sequenced using R9.4.1 Flongle flow cells (FLO-FLG001) or R9.4.1 minION flow cells until approximately 200$\times$ coverage was obtained for each barcode based on an expected plasmid size of 20 kilobases. Basecalling was perfomed using Guppy v4.2.2 in high-accuracy mode (Oxford Nanopore Technologies). Reads were filtered by retaining only those near the expected plasmid length. Reads were then assembled using miniasm^58^. The assembly was then polished using minipolish^59^ and medaka (Oxford Nanopore Technologies). Polished assemblies were then compared to the expected sequence to determine if any mutations were present.

### RNAseq analysis

Total RNA was extracted from 15 mL cultures with an $\hbox {A}_{670}$ of $\sim$ 0.6 to 0.7 by first crushing the algal cells in liquid nitrogen as follows. Cultures were centrifuged at 3000$\times$*g* for 15 mins at 4 $^{\circ }$C. The pellet was resuspended in $\sim$ 100 to 500 $\upmu$L TE pH 8.0 and added dropwise to a mortar (pre-cooled at − 80 $^{\circ }$C) filled with liquid nitrogen. The frozen droplets were ground into a fine powder with a mortar and pestle, being careful to keep the cells from thawing by adding more liquid nitrogen when necessary. The frozen ground powder was transferred to a new clean 1.5 mL microfuge tube and stored at − 80 $^{\circ }$C. RNA was extracted from 50 to 100 mg of frozen ground powder with the Monarch Total RNA Miniprep Kit (T2010S) following the plant protocol. The RNA was stored in TE pH 8.0 at $-80\, ^{\circ }$C until use. Quantity and purity were measured by spectrophotometer, and RNA integrity was evaluated using a 1% pre-stained agarose gel run at 100 V for 30 min. RNA integrity was further evaluated using an Agilent Bioanalyzer. rRNA was depleted using the Vazyme Ribo-off plant rRNA depletion kit (N409). Sequencing libraries were then prepared for two different RNAseq experiments; the first for cells harbouring pSS1 and wild-type cells, and the second for clones of pSS1, pSS2 and pSS7. Libraries were sequenced at the London Regional Genomics Center using an Illumina NextSeq high output single end 75 run for the first experiment, and a high output single end 150 run for the second experiment. Reads were trimmed to 75 base pairs and aligned against the ASM15095v2 reference assembly and expected plasmid sequence using hisat2^60^. Coverage was determined using Mosdepth^61^.

### Diagnostic PCR assays

For direct PCR assays, colonies of *P. tricornutum* transformants were screened for the presence of the RBD gene using a Thermo Scientific Phire Plant Direct PCR Master Mix according to manufacturers instructions. PCR screens were performed using a forward primer located in the *HASP1* promoter (DE5241) for *P. tricornutum* transformed with pSS1 and pSS2, or in the *40SRPS8* promoter (DE4130) for *P. tricornutum* transformed with pSS7. Reverse primers were positioned inside the RBD domain coding regions (DE5323, DE5326). Screening primers are listed in Supplementary Table 5.

### Transfer of DNA to *P. tricornutumvia* conjugation from *E. coli*

Conjugations were performed as previously described^19,33^. Briefly, liquid cultures (250 $\upmu$L) of *P. tricornutum*, adjusted to a density of 1.0 $\times 10^{8}$ cells $\hbox {mL}^{-1}$ using counts from a hemocytometer, were plated on $\frac{1}{2} \times$ L1 1% agar plates with or without histidine (200 mg $\hbox {L}^{-1}$), and grown for four days. L1 media (1.5 mL) was added to the plate and cells were scraped and the concentration was adjusted to 5.0 $\times 10^{8}$ cells $\hbox {mL}^{-1}$. *E. coli* cultures (50 mL) were grown at 37 $^{\circ }$C to $\hbox {A}_{600}$ of 0.8–1.0, centrifuged for 10 mins at 3000$\times$*g* and resuspended in 500 $\upmu$L of SOC media. Conjugation was initiated by mixing 200 $\upmu$L of *P. tricornutum* and 200 $\upmu$L of *E. coli* cells. The cell mixture was plated on $\frac{1}{2} \times$ L1 5% LB 1% agar plates, incubated for 90 mins at 30 $^{\circ }$C in the dark, and then moved to 18 $^{\circ }$C in the light and grown for 2 days. After 2 days, L1 media (1.5 mL) was added to the plates, the cells scraped, and 300 $\upmu$L (20%) plated on $\frac{1}{2} \times$ L1 1% agar plates supplemented with Zeocin 50 mg $\hbox {L}^{-1}$ or nourseothricin 100 mg $\hbox {L}^{-1}$. Colonies appeared after 7–14 days incubation at 18 $^{\circ }$C with light.

### Measuring growth and eGFP production of *P. tricornutum* cultures

*Phaeodactylum tricornutum* cultures were adjusted to an $\hbox {A}_{670}$ of 0.05 in L1 media made without phosphate, nitrate, or iron. Cultures were then washed by centrifugation for 10 mins at 3000$\times$*g* followed by resuspension in fresh L1 media without phosphate, nitrate, or iron. Phosphate, nitrate, and iron stock solutions were then used to adjust cultures to the follow conditions: full L1 or L1 with 5% phosphate, 5% nitrate, 5% iron, 5% phosphate and 5% iron, 5% phosphate and 5% nitrate, or 5% nitrate and 5% iron. Cultures were grown at 18 $^{\circ }$C under cool white fluorescent lights (75 $\upmu$E $\hbox {m}^{-2} \hbox {s}^{-1}$) and a photoperiod of 16 h light:8 h dark for 28 days, and absorbance at 670 nm ($\hbox {A}_{670}$) or 750 nm ($\hbox {A}_{750}$) was measured every 48 h using an Ultrospec 2100 pro UV/vis spectrophotometer. Samples for fluorescence readings and western blots were taken every 4 days by centrifuging 700 $\upmu$L of culture at 16,000$\times$*g* for 15 min. Three 200 $\upmu$L aliquots of the supernatant were pipetted into a clear bottom 96 well plate for fluorescence readings. Another 44 $\upmu$L of supernatant was mixed with 22 $\upmu$L of 3$\times$ SDS sample loading buffer (187.5 mM Tris-HCl (pH 6.8), 6% (w/v) SDS, 30% [v/v] glycerol, 150 mM DTT, 0.03% (w/v) bromophenol blue, 2% [v/v] $\beta$-mercaptoethanol) and boiled at 95 $^{\circ }$C for 10 mins, after which 15 $\upmu$L of boiled sample was analyzed by western blot. The pellet was resuspended in 50 $\upmu$L of 3$\times$ SDS sample loading buffer and boiled at 95 $^{\circ }$C for 10 mins, after which 10 $\upmu$L of boiled sample was analyzed by western blot. Fluorescence readings were taken in a Biotek Synergy H1 platereader at an excitation wavelength of 475 nm and emission wavelength of 515 nm. Fluorescence values obtained were subtracted from wildtype autofluorescence in the supernatant. Fluorescence values were converted to eGFP in $\upmu$g $\hbox {mL}^{-1}$ using a standard curve generated using commercially available purified eGFP.

### Photobioreactor conditions for growth of *P. tricornutum*

A 5 L photobioreactor system (Eppendorf Canada) was used for the growth of *P. tricornutum* at bench scale. Temperature was controlled in the photobioreactor at 18 $^{\circ }$C. Mixing was achieved with a single pitched-blade impeller at 100 rpm. A constant gas flow of 0.75 VVM was sparged into the reactor with a mix of 0.5% carbon dioxide and 99.5% air. The pH of the culture was controlled at 8.1 using a cascade with carbon dioxide from 0.5 to 5% v/v mix. Light was provided by continuous (24 h/day) full spectrum LED grow lights with 5 bulbs at a light intensity of approximately 50 $\upmu$mol $\hbox {m}^{-2} \hbox {s}^{-1}$. Samples were collected daily for optical density, cell count and compositional analysis. A 10% inoculum was used to attain a minimum cell density of 2 million cells $\hbox {mL}^{-1}$. The inoculum was cultured in an incubator (Innova S44i, Eppendorf, Hamburg, Germany) with a photosynthetic light bank containing LED lighting. Lighting for the inoculum was at an intensity of approximately 65 $\upmu$mol $\hbox {m}^{-2} \hbox {s}^{-1}$ with a cycling of 16 h on and 8 h off. Temperature was controlled at 18 $^{\circ }$C with orbital agitation at 100 rpm. pH was not controlled and no gas was sparged into the inoculum.

### Compositional analysis of media

The concentration of dissolved phosphorus and iron was measured by Inductively Coupled Plasma-Optical Emission Spectrometry (ICP-OES). An Agilent 5110 (Agilent, USA) spectrometer ICP-OES equipped with a Seaspray concentric glass nebulizer (Agilent, USA) and an SPS 4 auto sampler was used. Argon (purity higher than 99.995%) supplied by Linde Canada was used to sustain plasma and, as carrier gas. The operating conditions employed for ICP-OES determination of iron were 1200 W RF power, 12 L $\hbox {min}^{-1}$ plasma flow, 1.0 L $\hbox {min}^{-1}$ auxiliary flow, 0.7 L $\hbox {min}^{-1}$ nebulizer flow, with radial view used for determination. The operating conditions employed for ICP-OES determination of phosphorus were 950 W RF power, 12 L $\hbox {min}^{-1}$ plasma flow, 1.0 L $\hbox {min}^{-1}$ auxiliary flow, 0.95 L $\hbox {min}^{-1}$ nebulizer flow, with axial view used for determination. The most sensitive lines free of spectral interference were used to determine emission intensities. The calibration standards were prepared by diluting a phosphorus and iron standards (Agilent, USA) in synthetic seawater and 1% (v/v) nitric acid. The calibration curve for phosphorus was in the range of 0.1–10 ppm and for iron was in the range of 0.05–5 ppm.

### Protein extraction and purification

*Phaeodactylum tricornutum* cultures (5 L) were harvested during stationary growth phase and pelleted at 3000$\times$*g* for 10 mins at 4 $^{\circ }$C. Cell pellets were resuspended in lysis buffer (50 mM Tris-HCl pH 7.4, 0.5 M NaCl, 10 mM imidazole, 0.1% Tween-20, 1 mM DTT, 1 $\times$ Protease inhibitor cocktail (Sigma)) and homogenized on an Emulsiflex C3 Homogenizer with 5 passes at 20,000 psi to lyse. Lysates were then sonicated with two 15 s pulses, resting on ice for 30 s between pulses. Sonicated lysates were centrifuged at 20,000$\times$*g* for 30 mins at 4 $^{\circ }$C to pellet cell debris, and supernatants were collected in a new tube and stored on ice before purification using a 20 mL GE Healthcare HisPrep FF 16/10 Ni-sepharose column as follows.

All samples were run on an AKTA Pure FPLC system at 4 $^{\circ }$C. Ni-sepharose columns were first washed with 10 column volumes of $\hbox {ddH}_{2}\mathrm{O}$, then equilibrated with 10 column volumes of lysis buffer. Supernatants from lysed cultures were run over equilibrated columns at a flow rate of 5 mL $\hbox {min}^{-1}$ and flowthrough was collected. Columns were washed with 10 column volumes lysis buffer at 5 mL $\hbox {min}^{-1}$, followed by 10 column volumes wash buffer (50 mM Tris-HCl pH 7.4, 0.5 M NaCl, 50 mM imidazole, 0.1% Tween-20, 1 mM DTT) at 5 mL $\hbox {min}^{-1}$. His-tagged proteins were eluted with 4 column volumes elution buffer (50 mM Tris-HCl pH 7.4, 0.5 M NaCl, 250 mM imidazole, 0.1% Tween-20, 1 mM DTT) at 1 mL $\hbox {min}^{-1}$, collecting 5 mL fractions. Samples (20 $\upmu$L) of lysis supernatant, flowthrough, washes, and elution fractions were mixed with 10 $\upmu$L of 3$\times$ SDS sample loading buffer (187.5 mM Tris-HCl (pH 6.8), 6% (w/v) SDS, 30% [v/v] glycerol, 150 mM DTT, 0.03% (w/v) bromophenol blue, 2% [v/v] $\beta$-mercaptoethanol) and boiled at 95 $^{\circ }$C for 5 mins. Boiled samples (15 $\upmu$L) were resolved on standard SDS-polyacrylamide gels (15%). Bands were visualized with Coommassie Brilliant Blue and destained with a solution of 40% methanol, 10% acetic acid.

Ni-sepharose elution fractions containing RBD protein were pooled and dialyzed at 4 $^{\circ }$C for 5 h in 1 L of IEX loading buffer (50 mM HEPES pH 8.0, 10 mM NaCl, 1 mM DTT), then overnight in 2 L of fresh IEX loading buffer. The dialyzed sample was then loaded onto a 5 mL SP HP HiTrap column at 0.5 mL $\hbox {min}^{-1}$ and flowthrough was collected. Columns were washed with 10 column volumes IEX wash buffer (50 mM HEPES pH 8.0, 25 mM NaCl, 1 mM DTT) at 2 mL $\hbox {min}^{-1}$. Bound proteins were eluted with IEX elution buffer (50 mM HEPES pH 8.0, 500 mM NaCl, 1 mM DTT) at 1 mL $\hbox {min}^{-1}$, collecting 1 mL fractions. Samples were analyzed on SDS-polyacrylamide gels as above.

IEX elution fractions containing RBD protein were pooled, concentrated using Pierce centrifugal protein concentrators (10 kDa cutoff), and loaded onto a Superdex 200 Increase 10/300 GL column (24 mL bed volume) followed by IEX loading buffer at 0.5 mL $\hbox {min}^{-1}$. Flowthrough was collected in 0.5 mL fractions. Samples were analyzed on SDS-polyacrylamide gels as above. Elution fractions containing RBD protein were pooled, then concentrated and buffer exchanged with PBS using Pierce centrifugal protein concentrators (10 kDa cutoff).

### Western blots

Samples were resolved on standard SDS-polyacrylamide gels (15%) and electroblotted to a polyvinylidene difluoride (PVDF) membrane using a Trans-Blot Turbo Transfer System (BioRad, Hercules, CA, USA). Mammalian expressed RBD (HEK293-RBD) was purchased from Sino Biological, 40592-V08H. Membranes were incubated for 1 hour in blocking solution (3% bovine serum albumin (BSA), 0.1% Tween-20, 1$\times$ TBS) before adding anti-RBD primary antibody (Sino Biological, 40592-T62) at a 1:1000 final dilution, or anti-GFP primary antibody (Invitrogen, A-6455) at a 1:2500 final dilution, or anti-6His primary antibody (Invitrogen, MA1-21315) at a 1:1000 final dilution. Membranes were incubated overnight at 4 $^{\circ }$C, washed for 3 $\times$ 10 mins in washing solution (1% BSA, 0.1% Tween-20, 1$\times$ TBS), then incubated with anti-rabbit (Sigma, GENA9340) or anti-mouse (Amersham, NA931) horseradish peroxidase-linked secondary antibody for 2 h at 1:5000 final dilution in washing solution. Membranes were then washed in 1$\times$ TBS with 0.1% Tween-20 for 3 $\times$ 10 mins, followed by one wash for 10 mins in 1$\times$ TBS. Blots were developed using Clarity ECL western blotting Substrate (BioRad) following the manufacturer’s instructions and imaged with a ChemiDoc XRS+ System (Bio-Rad).

### Mass spectrometry

Protein samples were resolved on 8% (full length spike) or 15% (RBD) SDS-PAGE gels. Bands were visualized with Coommassie Brilliant Blue and destained with a solution of 40% methanol, 10% acetic acid. Bands were excised and placed in 1.5 mL microfuge tubes with 500 $\upmu$L of 1% acetic acid. Mass spectrometry analysis and peptide identification by ms/ms was performed at the SPARC BioCentre, Sick Kids Hospital, University of Toronto. Peptide data was downloaded from the SPARC BioCentre server and analyzed by the Scaffold software package using a FASTA file of the *P. tricornutum* and SARS-CoV-2 proteomes. The protein threshold was set at 90% and the peptide threshold at a 1% false discovery rate.

### Endoglycosidase assays

Purified protein samples (20 ng algae-RBD or HEK293-RBD) were treated with PNGase F or Endo H (NEB) according to manufacturers instructions following the denaturing reaction conditions protocol in a total reaction volume of 20 $\upmu$L. 10 $\upmu$L of 3$\times$ SDS sample loading buffer was then added to each 20 $\upmu$L reaction and boiled at 95 $^{\circ }$C for 5 mins. Boiled samples (15 $\upmu$L) were resolved on standard SDS-polyacrylamide gels (15%) and analyzed by western blot.

### ACE2-binding assay

ACE2-binding assays were performed on the algae-RBD and HEK293-RBD using a COVID-19 Spike-ACE2 Binding Assay Kit (RayBio, CoV-ACE2S2-2) according to manufacturers instructions.

### Gold conjugation of algae-RBD

For the preparation of gold labelled algae-RBD, 40 nm colloidal gold particles (International Point of Care Inc) were adjusted to a pH of 9.45 (± 0.15) using 1.0 M potassium carbonate buffer. The pH-adjusted gold was then diluted in ultrafiltrated $\hbox {H}_{2}\mathrm{O}$ to an optical density (OD) of 1.0 (peak absorbance observed between wavelength 350–540 nm). Purified algae-RBD (0.213 mg/mL) was conjugated by passive adsorption to gold at a final concentration of 6 $\upmu$g/mL algae-RBD per mL of OD 1.0 gold. After incubation at ambient temperature (22–25 $^{\circ }$C) for 45 min, a solution of 10% bovine serum albumin and 1% polyethylene glycol comprising 5% of the total conjugate volume was added and incubated at ambient temperature for 30 mins under gentle mixing. After incubation the gold conjugate solution was centrifuged at 12,000$\times g$ for 30 mins. Without disturbing the pellet, the resulting supernatant was removed and discarded. The pellet was then resuspended in 20 mM Tris-HCl (pH 9.25) representing 80% of the initial conjugate volume. This process was repeated in two consecutive centrifugation and resuspension steps. A final centrifugation step was performed and the supernatant discarded. The pellet was resuspended in the remaining supernatant. The RBD labelled gold was then stored overnight at 2–8 $^{\circ }$C to observe aggregation. Following overnight incubation, algae-RBD gold conjugate was briefly sonicated in a water bath to disperse any gold aggregates. A sample of the gold conjugate was then diluted 1/50 in Tris-HCl pH 9.25 and the absorbance peak was measured. The final OD of the gold conjugate was determined to be 12.8.

### Preparation and testing of LFA devices

A commercially available qualitative lateral flow COVID-19 serology test (Lumivi) was adapted to evaluate the algae-RBD that was lyophilized onto polyester pads. Nitrocellulose membrane (Millipore) was striped at a test line location with an anti-human IgG antibody (BiosPacific Inc.) and at a control line location with a Goat anti-Rabbit polyclonal antibody (Cedarlane Inc.). Following lamination onto adhesive lined polyester backing cards, the resulting cards were cut into cut into 5.5 mm strips and placed into the Lumivi commercial housing, packaged in foil pouches with desiccant and stored at ambient temperature for subsequent testing. For comparison purposes a SARS-CoV-2 Spike protein RBD domain expressed in a human cell line (DAGC174 Creative Diagnostics) was similarly conjugated to colloidal gold as described previously and evaluated in the adapted lateral flow assay. An 15 $\upmu$L aliquot each test sample (plasma) was applied to the device sample well followed by three drops of 0.1M PBS/Tween running buffer (approximately 150 $\upmu$L). After a 15-min incubation at ambient temperature the test was visually interpreted for the presence of purple/red lines at both the Test and Control marker areas within the devices read window. The devices were scanned with an optical reader (i-Lynx) and values of 0.055 reflectance units were considered to be visually detectable by untrained operators and are positive. Values below 0.055 reflectance units are scored as negative. A positive control line (indicating proper sample flow within the prototype device) was required before device interpretation could be made.

## Supplementary Information

Supplementary Information.
