# Supplementary material for: Phosphate-regulated expression of the SARS-CoV-2 receptor-binding domain in the diatom Phaeodactylum tricornutum for pandemic diagnostics
Source: Sci Rep. 2022 Apr 29;12:7010. doi: 10.1038/s41598-022-11053-7 (PMC9051505; doi:10.1038/s41598-022-11053-7)
Supplement: Supplementary file 1 — Supplementary Information. [file 41598_2022_11053_MOESM1_ESM.pdf]

Phosphate-regulated expression of the SARS-CoV-2 receptor-binding domain in the diatom *Phaeodactylum tricornutum* for pandemic diagnostics

Samuel S. Slattery[1], Daniel J. Giguere[1], Emily E. Stuckless[1], Arina Shrestha[1], Lee Ann Briere[1], Alexa Galbraith[2], Stephen Reaume[2], Xenia Boyko[1], Henry Say[1], Tyler Browne[1], Mallory Frederick[1], Jeremy Lant[1], Ilka Heinemann[1], Patrick O'Donoghue[1], Liann Dsouza[3], Steven Martin[3], Peter Howard[3], Garth Styba[4], Martin Flatley[5], Bogumil J. Karas[1], Gregory B. Gloor[1*], and David R. Edgell[1*]

[1]Department of Biochemistry, Schlich School of Medicine & Dentistry, Western University, London, Ontario, Canada N6A 5C1
[2]Lambton College, 457 London Rd, Sarnia, Ontario, Canada N7S 6K4
[3]Pond Technologies Inc., Markham, Ontario, Canada L3R 9W7
[4]International Point of Care Inc.,135 The West Mall Unit 9, Toronto, Ontario, Canada M9C 1C2
[5]Suncor Energy Inc., Sarnia Refinery, 1900 River Road, Sarnia, Ontario, Canada, N7T 7J3
[*]correspondence to: dedgell@uwo.ca, ggloor@uwo.ca

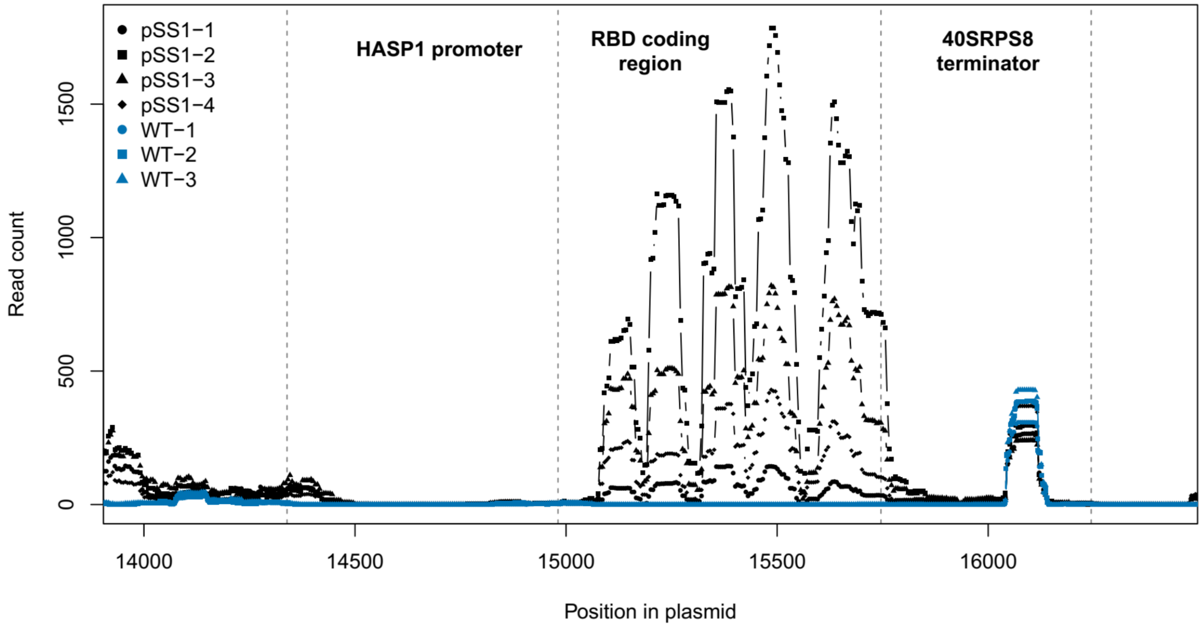

Figure S 1. Plot of RNAseq reads mapped to the pSS1 plasmid sequence for wild-type *P. tricornutum* (WT) and 4 clones harbouring pSS1.

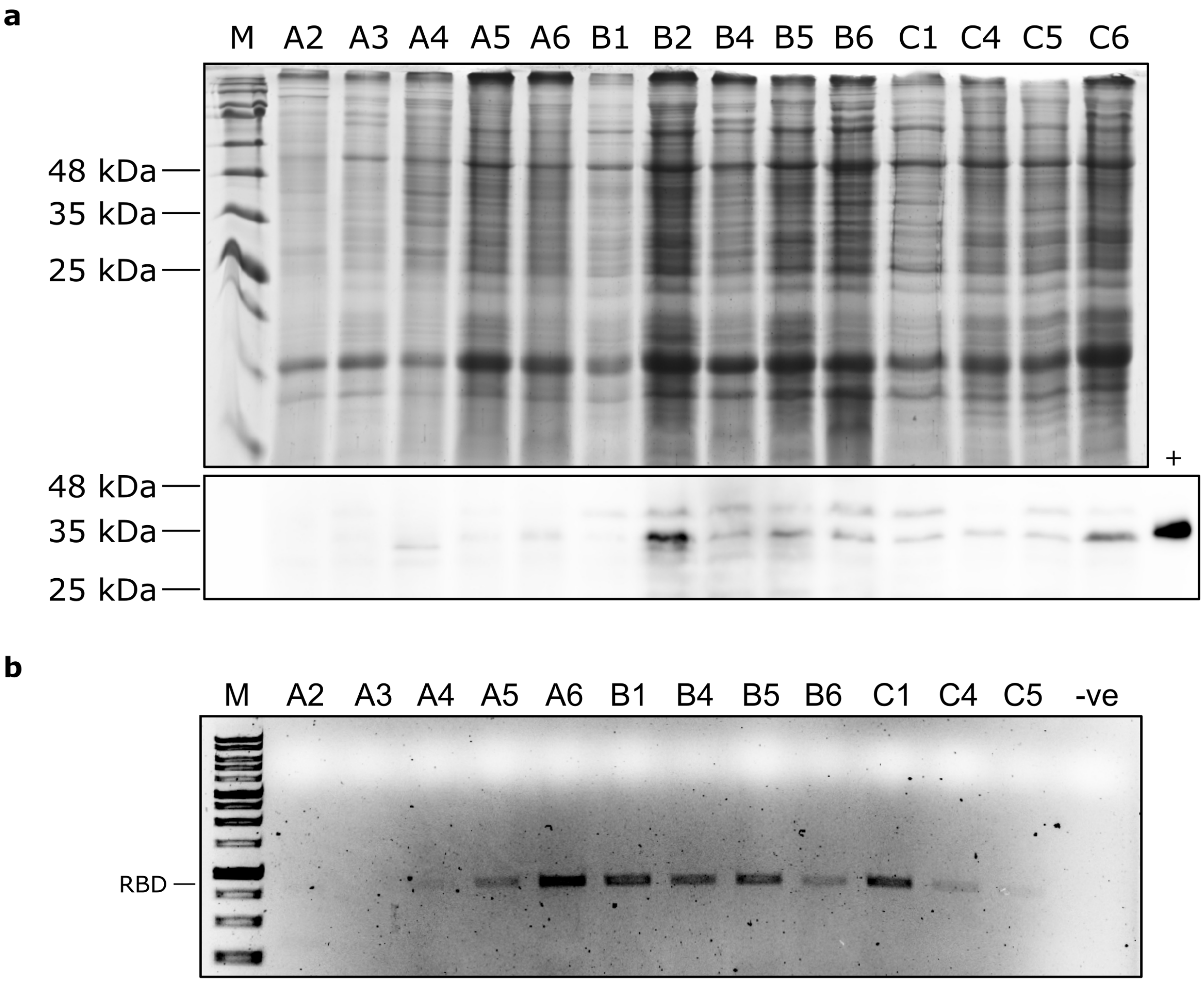

Figure S 2. Long-term stability of the RBD coding region. (A) Coomassie-stained gel of crude lysates from the indicated bioreactors (top) and Western blot using an anti-RBD polyclonal antibody (bottom). +, 5 ng of commercially available RBD purified from HEK293 cells. (B) PCR screen for RBD coding region from individual large-scale bioreactors as indicated. M, 1-kb ladder. The expected product is 833-bp. Uncropped gel images for panels (A) and (B) are shown in Supplementary Figures S28-S30.

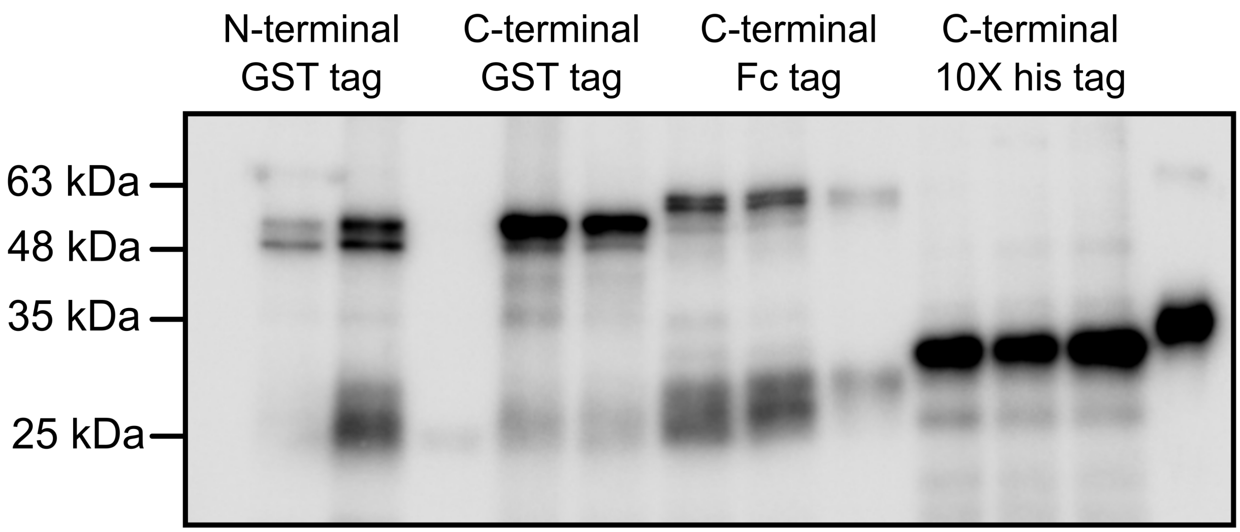

Figure S 3. Fusion of different purification tags to the RBD coding region. Shown is a Western blot using an anti-RBD antibody of three clones of each different fusion. The expected molecular weights are: RBD-Fc 51 kDa, the RBD-GST 51.5 kDa, and the RDB-10XHis 27.3 kDa. GST, glutathione S-transferase; Fc, IgG1-Fc tag; 10X his, C-terminal 10X-histidine tag. The (+) control is commercially purchased RBD made in HEK293 cells. An uncropped gel image is shown in Supplementary Figures S31.

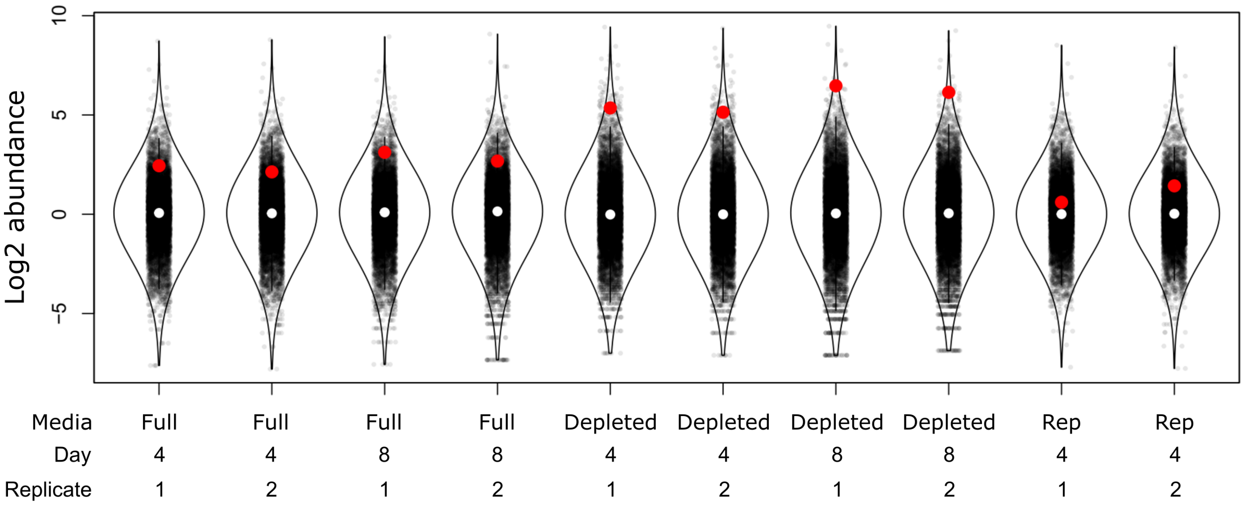

Figure S 4. *HASP1* expression in response to phosphate starvation. Shown is a violin plot of log2 abundance of transcripts mapped to the genome assembly of the *P. tricornutum* strain used in this study [1] from replicate experiments performed by Cruz de Carvalho et al. [2]. Black dots indicate mapped transcripts, the white dot is the mean abundance of all mapped transcripts per experiment, and the red dot is the abundance value for *HASP1*. Full, full media; Depleted, phosphate depleted media; Rep, phosphate replenishment in media.

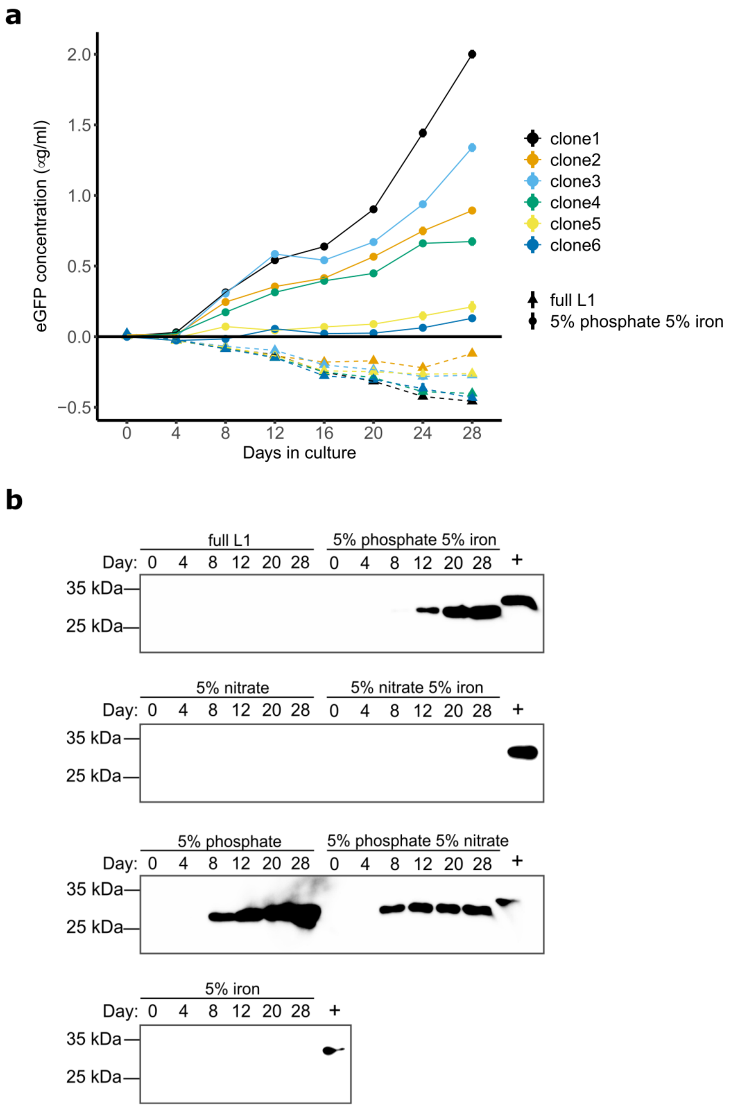

Figure S 5. eGFP secretion in phosphate and iron limitation conditions. A. Plot of time (days in culture) versus eGFP concentration in the culture supernatant for 6 independent transformants of pSS10 into *P. tricornutum*. Strains were grown in either full L1 media (filled triangles) or L1 media with 5% phosphate and 5% iron (filled circles). B. Western blot with anti-GFP antibody of whole cell lysates from pSS10 clone 6 grown in the indicated conditions and sampled at the indicated days. The (+) control is commercially available recombinant GFP. Uncropped gel images for panel (B) are shown in Supplementary Figures S32 and S33.

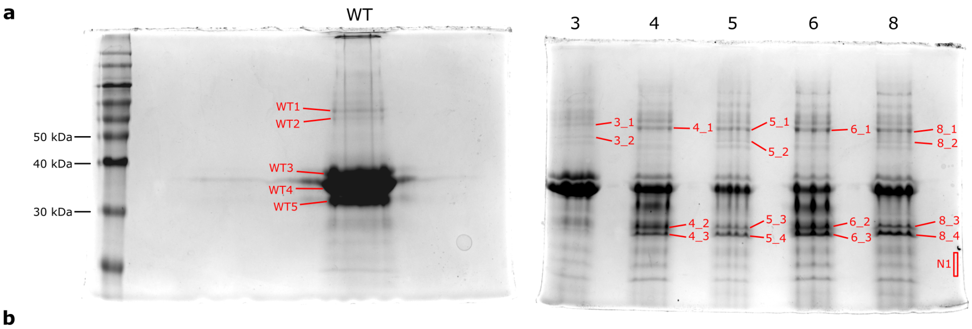

b

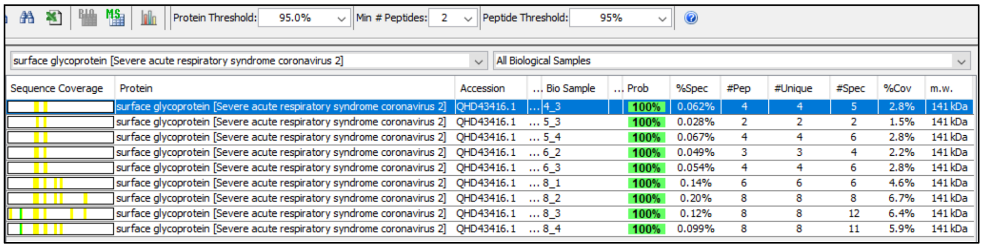
Protein Threshold: 95.0% | Min # Peptides: 2 | Peptide Threshold: 95%

surface glycoprotein [Severe acute respiratory syndrome coronavirus 2] | All Biological Samples

Sequence Coverage	Protein	Accession	... Bio Sample	... Prob	%Spec	#Pep	#Unique	#Spec	%Cov	m.w.
	surface glycoprotein [Severe acute respiratory syndrome coronavirus 2]	QHD43416.1	... 4_3	100%	0.062%	4	4	5	2.8%	141 kDa
	surface glycoprotein [Severe acute respiratory syndrome coronavirus 2]	QHD43416.1	... 5_3	100%	0.028%	2	2	2	1.5%	141 kDa
	surface glycoprotein [Severe acute respiratory syndrome coronavirus 2]	QHD43416.1	... 5_4	100%	0.067%	4	4	6	2.8%	141 kDa
	surface glycoprotein [Severe acute respiratory syndrome coronavirus 2]	QHD43416.1	... 6_2	100%	0.049%	3	3	4	2.2%	141 kDa
	surface glycoprotein [Severe acute respiratory syndrome coronavirus 2]	QHD43416.1	... 6_3	100%	0.054%	4	4	6	2.8%	141 kDa
	surface glycoprotein [Severe acute respiratory syndrome coronavirus 2]	QHD43416.1	... 8_1	100%	0.14%	6	6	6	4.6%	141 kDa
	surface glycoprotein [Severe acute respiratory syndrome coronavirus 2]	QHD43416.1	... 8_2	100%	0.20%	8	8	8	6.7%	141 kDa
	surface glycoprotein [Severe acute respiratory syndrome coronavirus 2]	QHD43416.1	... 8_3	100%	0.12%	8	8	12	6.4%	141 kDa
	surface glycoprotein [Severe acute respiratory syndrome coronavirus 2]	QHD43416.1	... 8_4	100%	0.099%	8	8	11	5.9%	141 kDa

Figure S 6. Mass spectrometry analysis of proteins from cell-free media of full length spike protein expression strains. (A) Coomassie-stained gel of concentrated supernatants from wild type *P. tricornutum* and wild type *P. tricornutum* harbouring pSS3 (3), pSS4 (4), pSS5 (5), pSS6 (6), or pSS8 (8). Bands excised for mass spectrometry analysis are indicated. (B) Mass spectrometry analysis of excised bands from panel A. Bio Sample, band identity from panel A; Prob, protein identification probability; %Spec, protein percentage of all spectra; #Pep, exclusive unique peptide count; #Unique, unique spectrum count; #Spec, total spectrum count; %Cov, percentage of amino acids identified or sequence coverage.

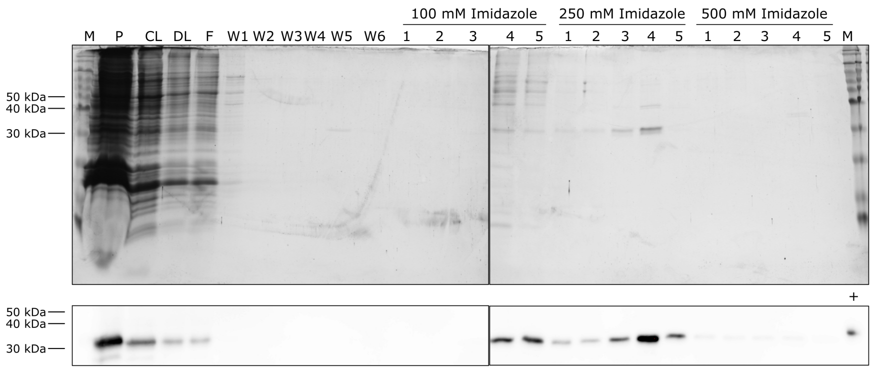

Figure S 7. Representative gel of purification of the algae-RBD under denaturing conditions. Top, coomassie-stained 15% SDS-PAGE gel of different fractions; bottom, Western blot using an anti-RBD antibody. M, marker; P, cell pellet; CL, clarified lysate; DL, denatured load; F, flow through; W1-W6, column wash steps. Uncropped gel images are shown in Supplementary Figures S34-S37.

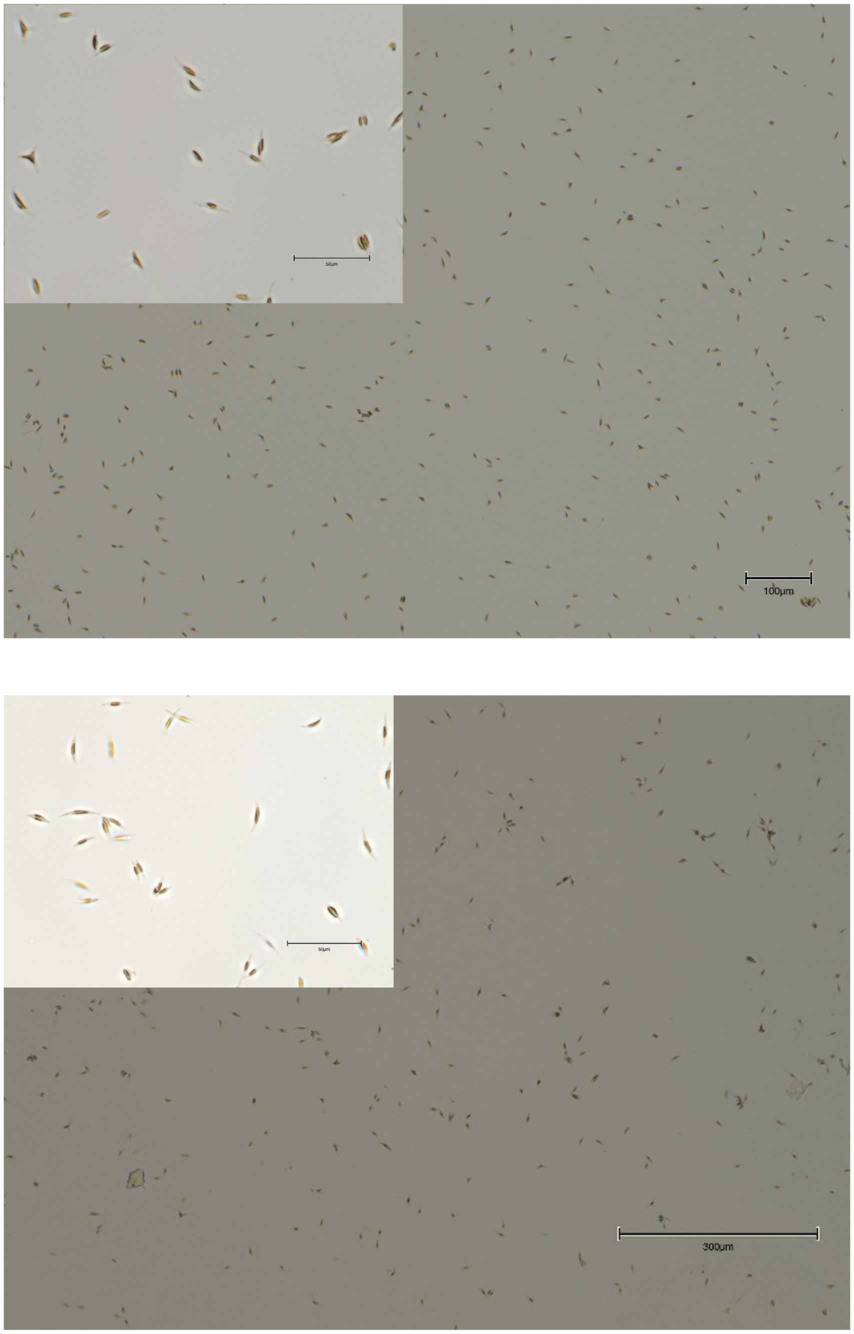

Figure S 8. Representative light microscopy images of *P. tricornutum* cultures used to screen for contamination.

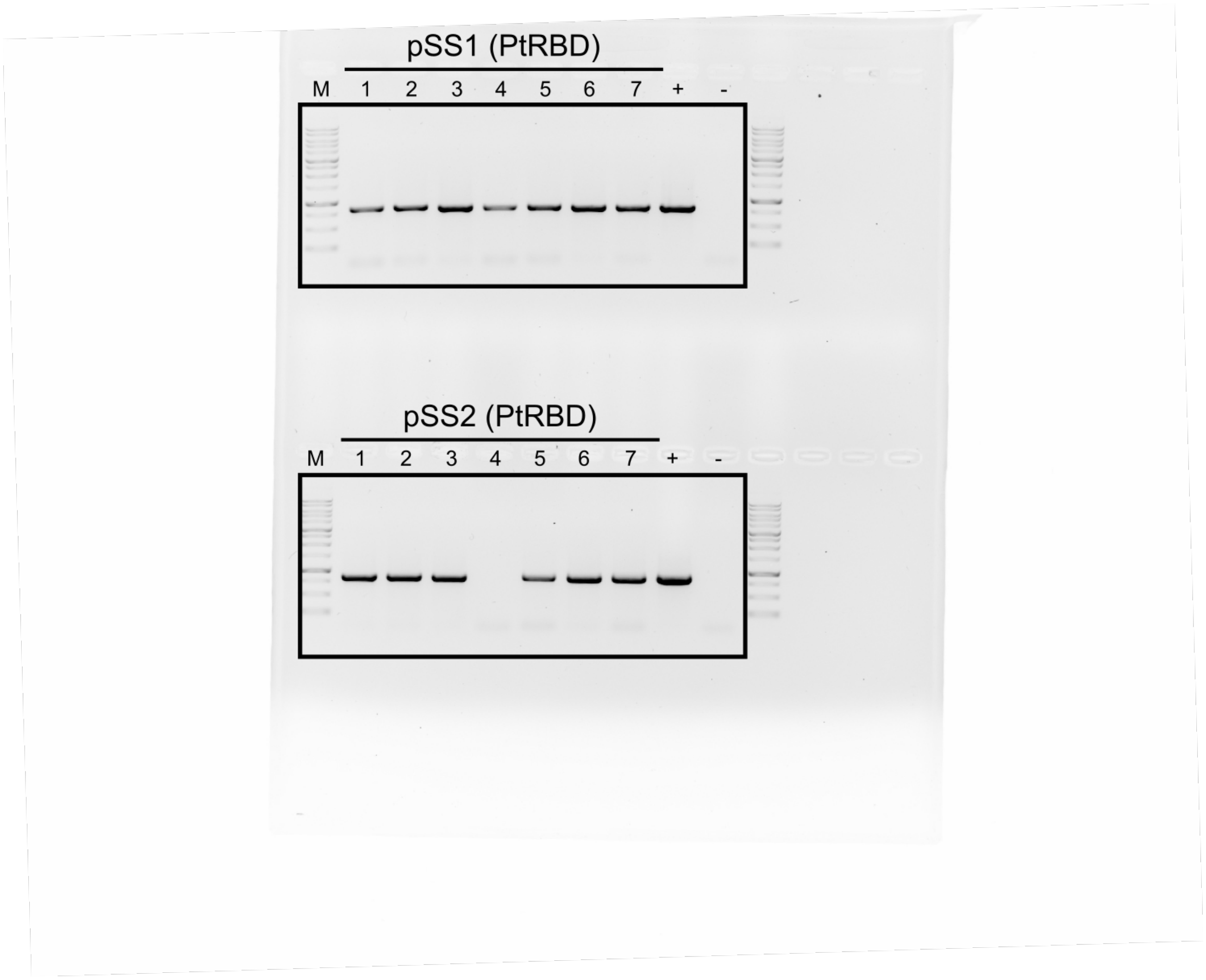

Figure S 9. The area cropped and shown in Fig 1B is indicated by a rectangle. Lanes are labeled as in Fig 1B.

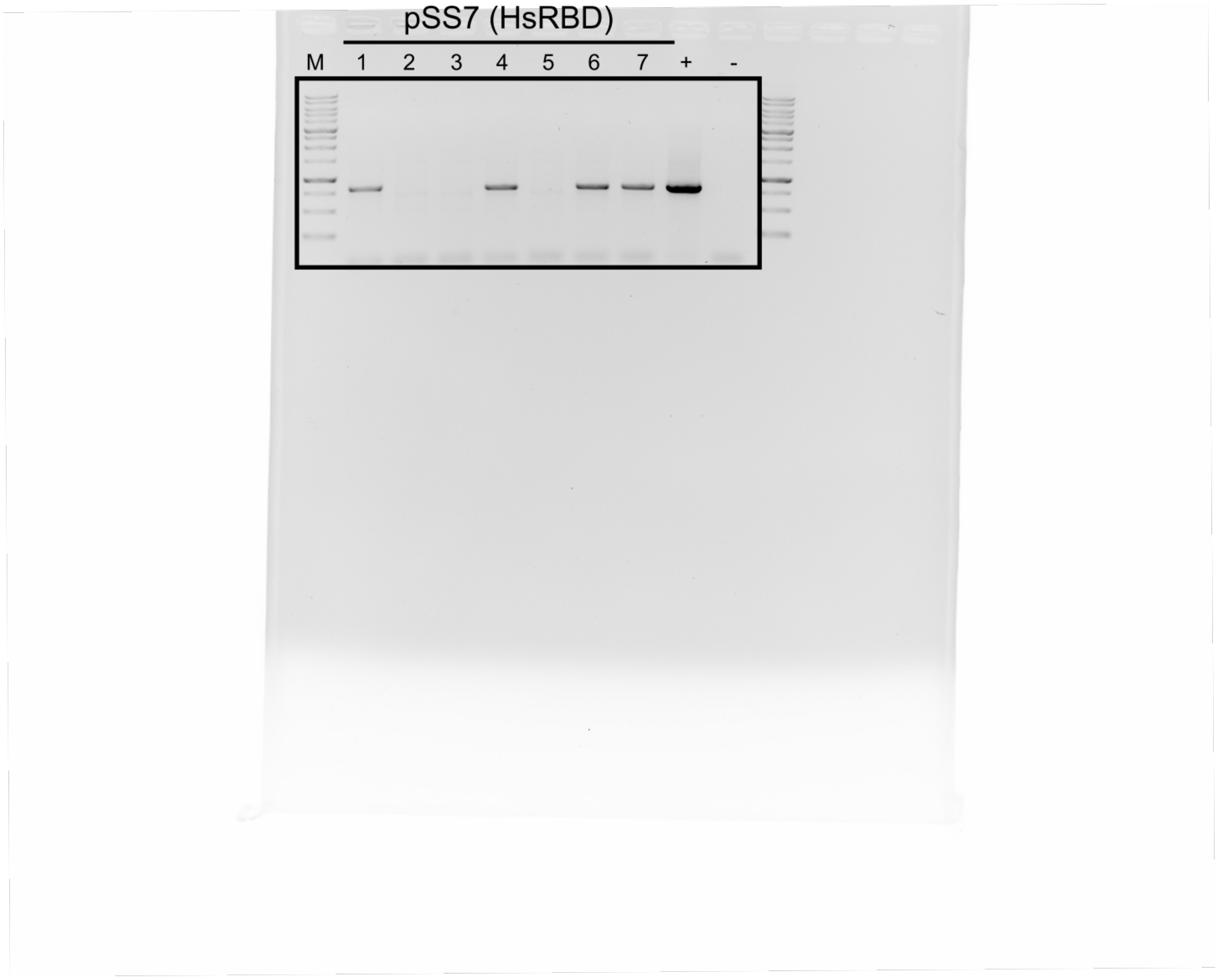

Figure S 10. The area cropped and shown in Fig 1B is indicated by a rectangle. Lanes are labeled as in Fig 1B.

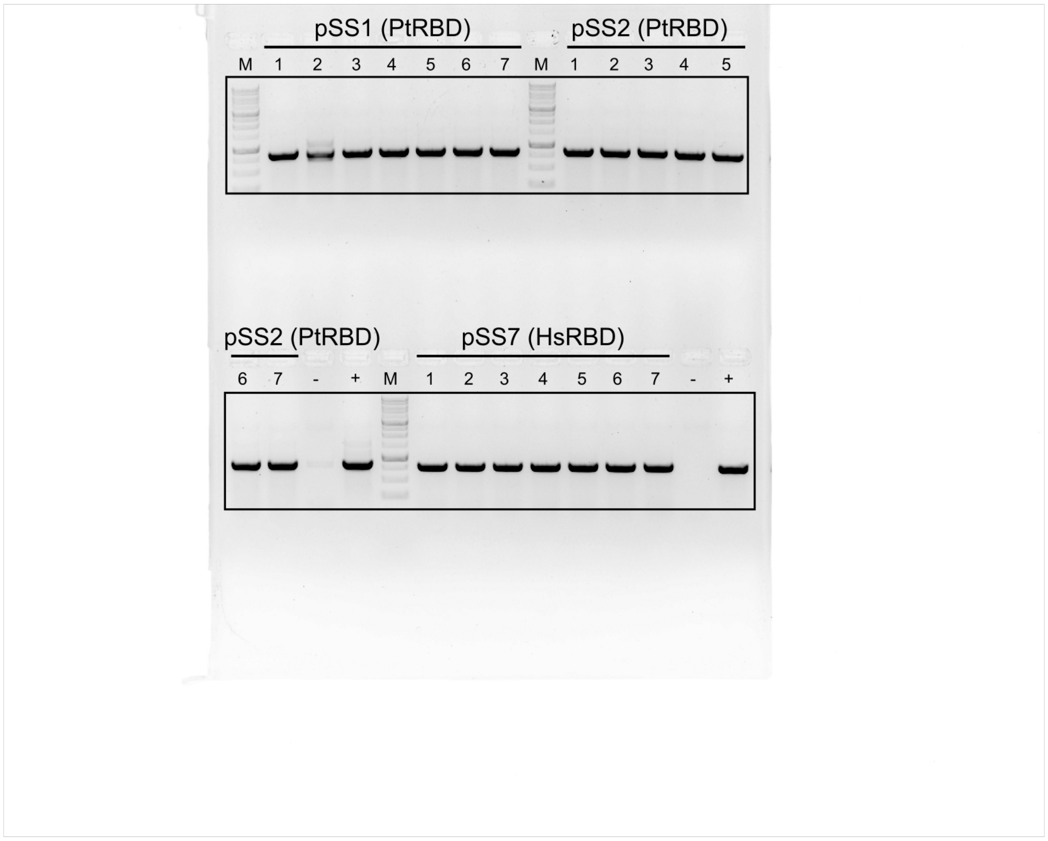

Figure S 11. The area cropped and shown in Fig 1B is indicated by a rectangle. Lanes are labeled as in Fig 1B.

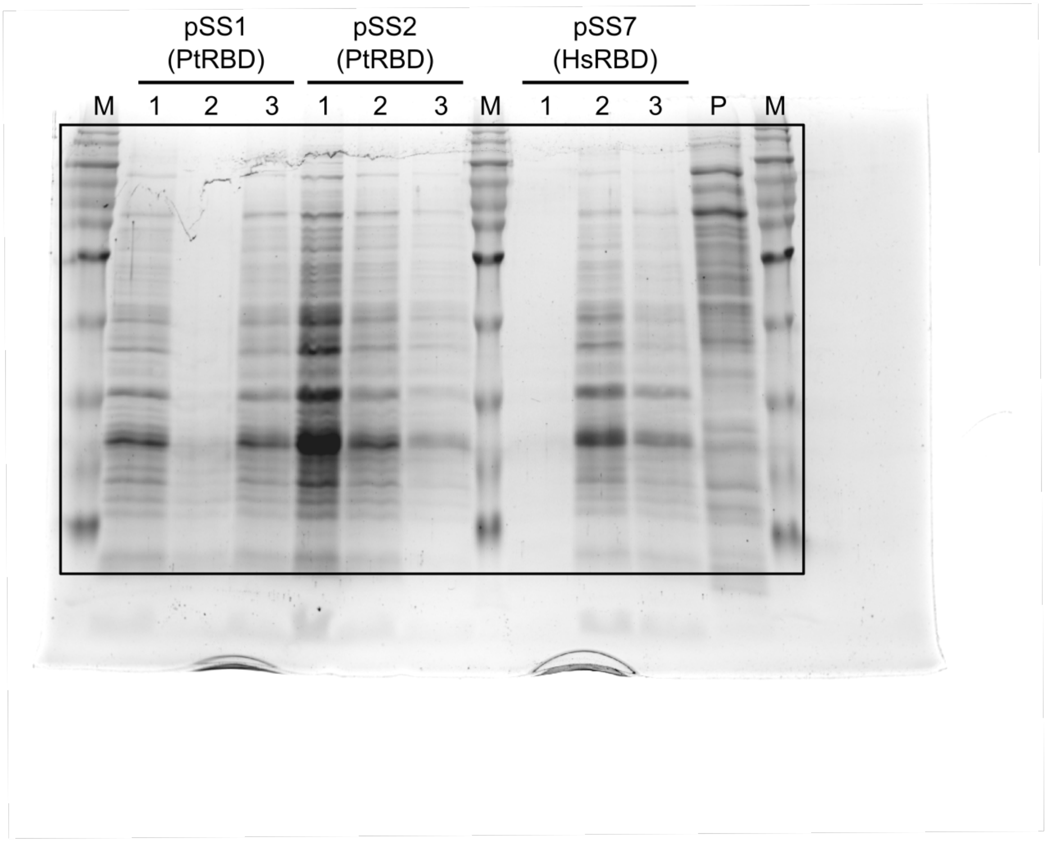

Figure S 12. The area cropped and shown in Fig 1D is indicated by a rectangle. Lanes are labeled as in Fig 1D.

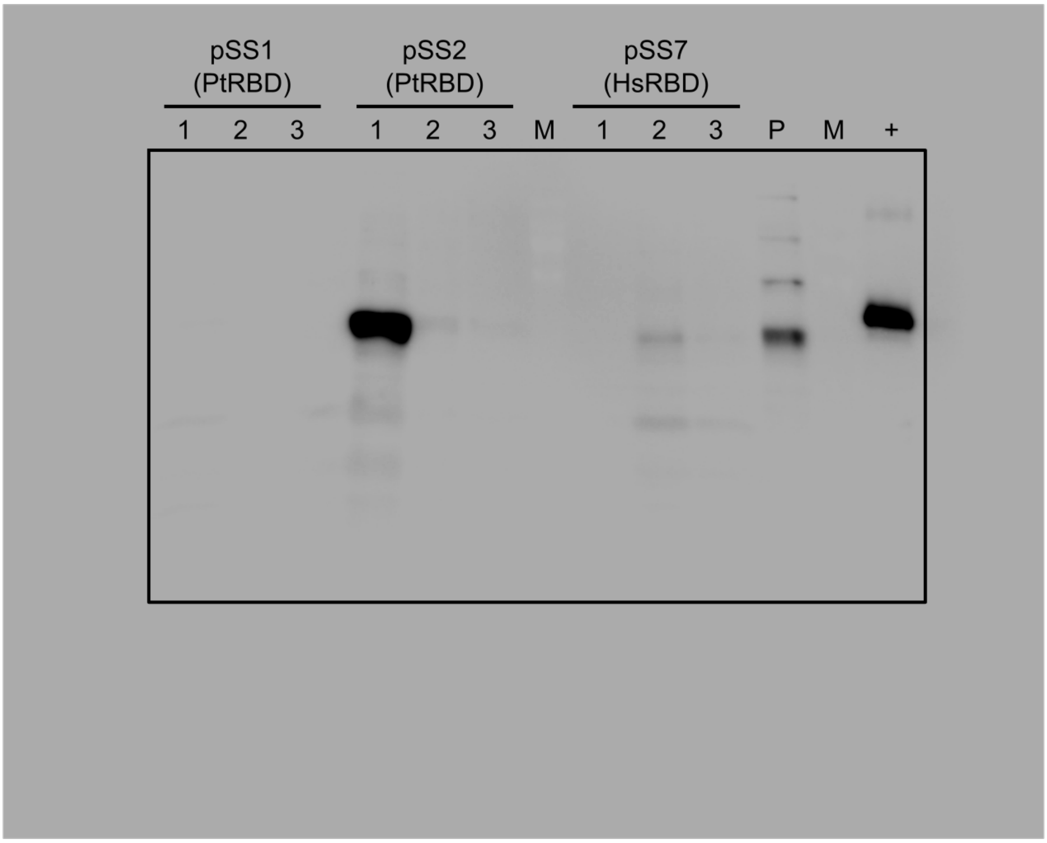

Figure S 13. The area cropped and shown in Fig 1D is indicated by a rectangle. Lanes are labeled as in Fig 1D.

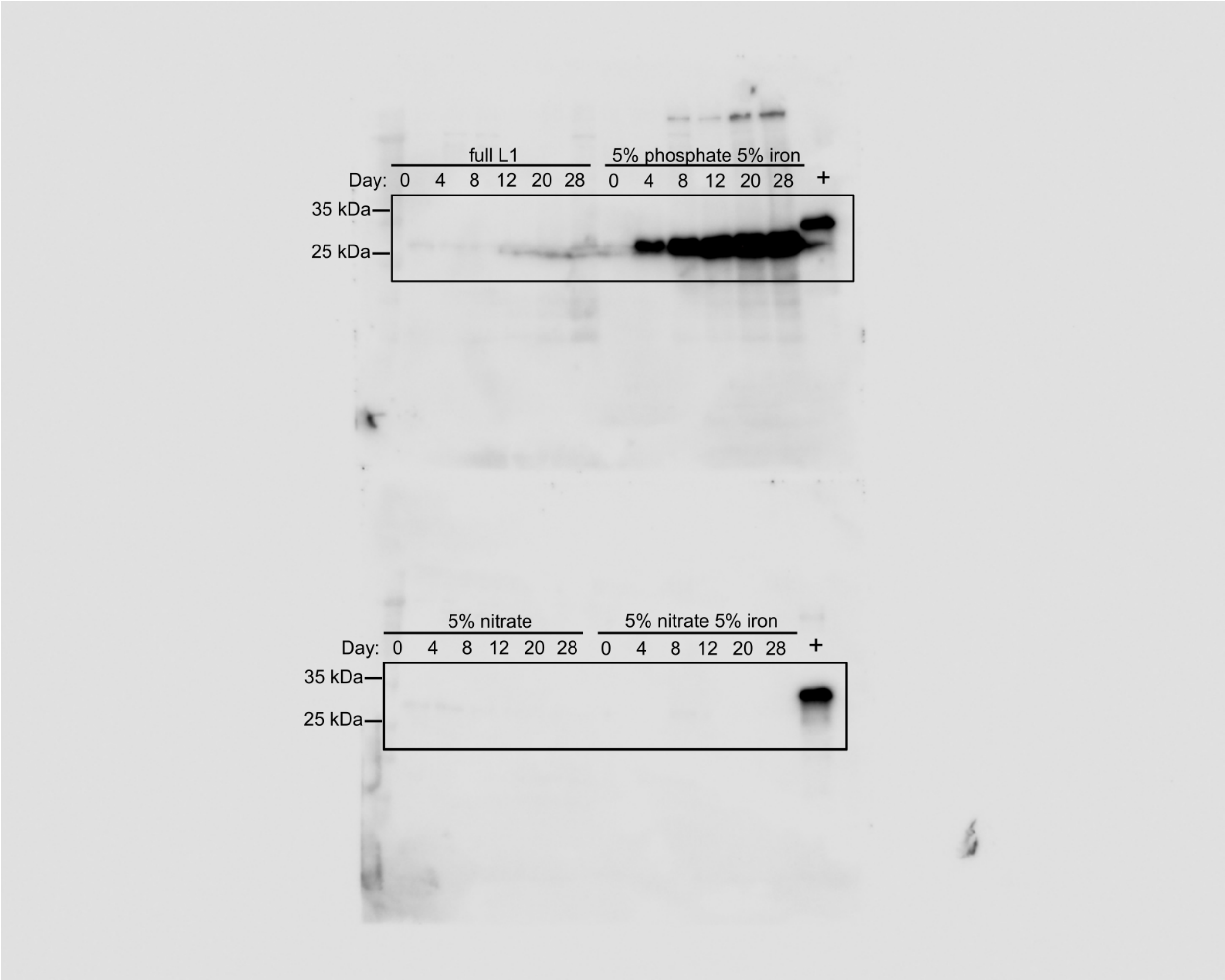

Figure S 14. The area cropped and shown in Fig 2B is indicated by a rectangle. Lanes are labeled as in Fig 2B.

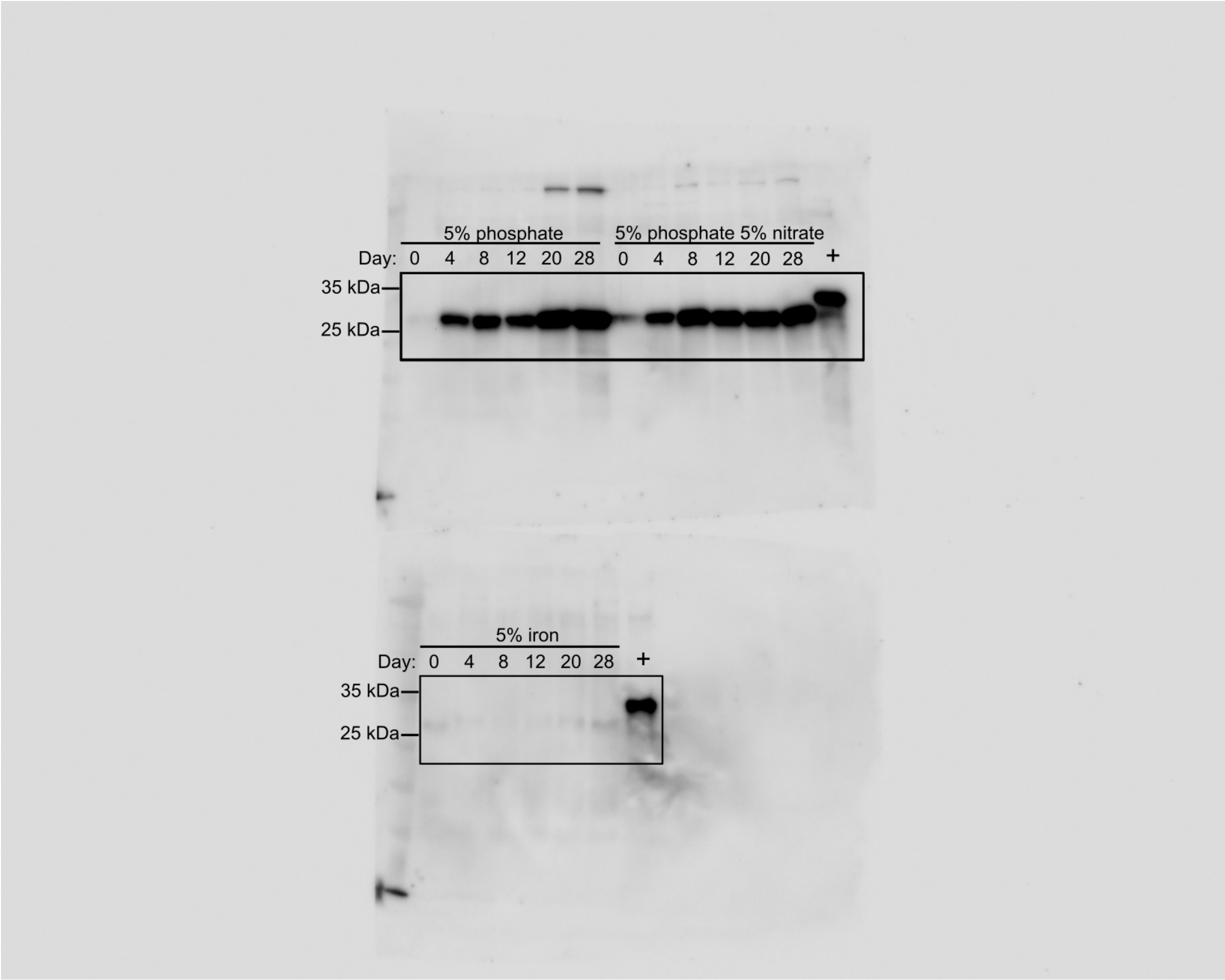

Figure S 15. The area cropped and shown in Fig 2B is indicated by a rectangle. Lanes are labeled as in Fig 2B.

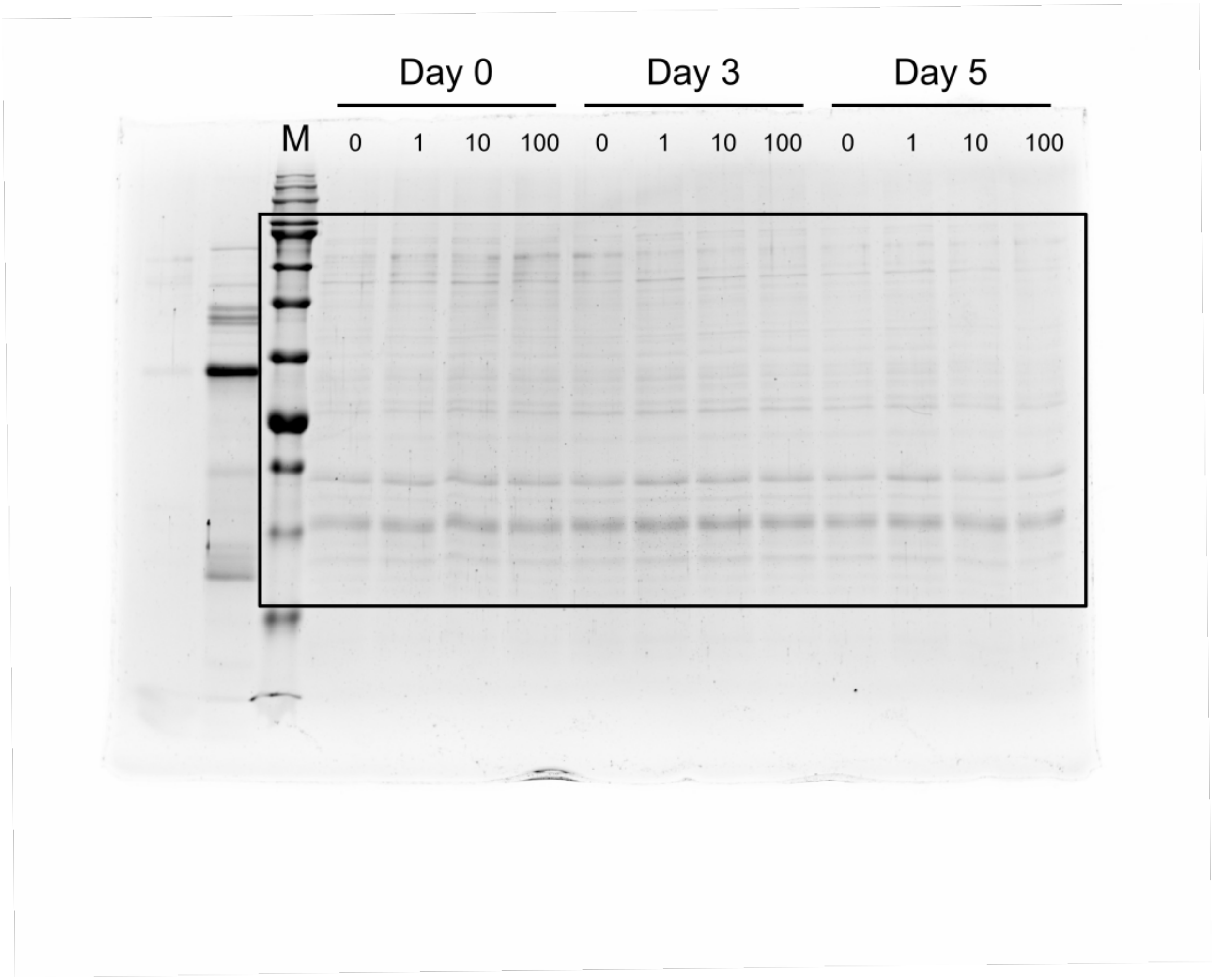

Figure S 16. The area cropped and shown in Fig 3A is indicated by a rectangle. Lanes are labeled as in Fig 3A.

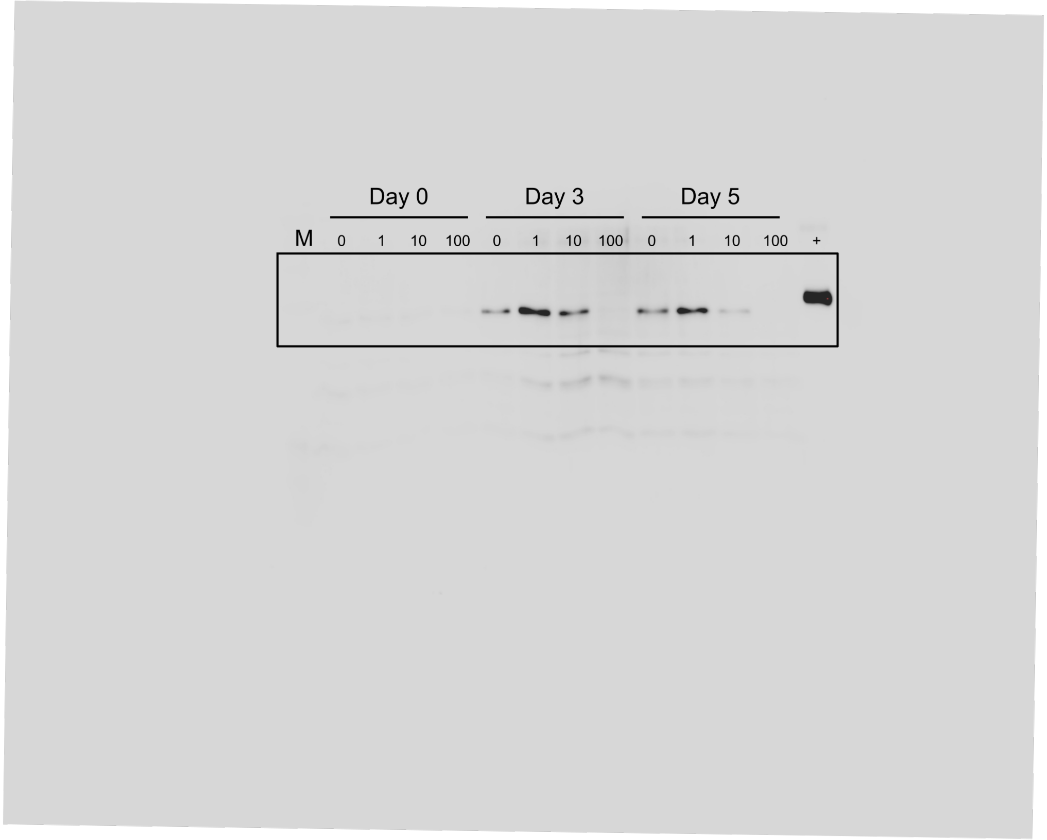

Figure S 17. The area cropped and shown in Fig 3A is indicated by a rectangle. Lanes are labeled as in Fig 3A.

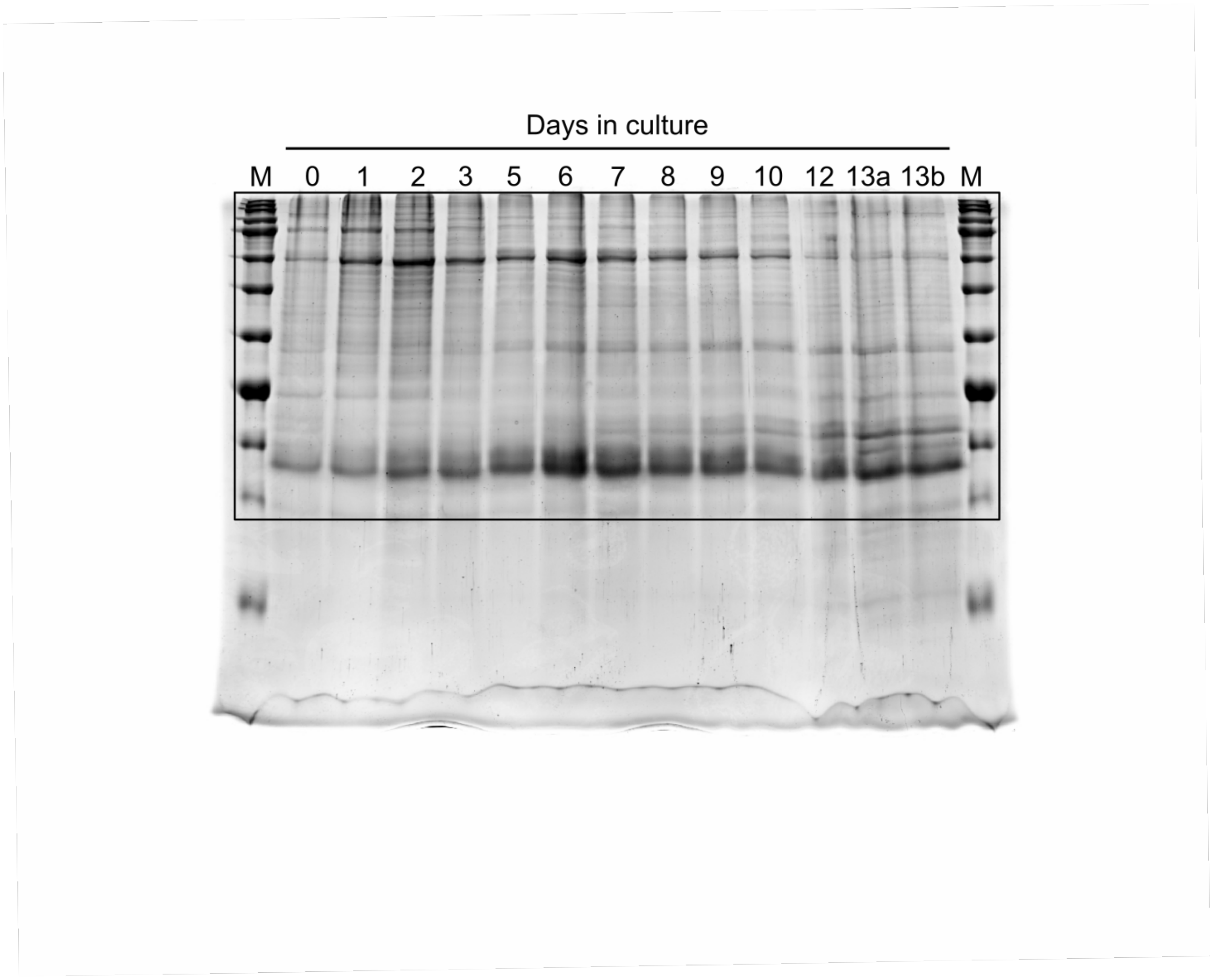

Figure S 18. The area cropped and shown in Fig 3B is indicated by a rectangle. Lanes are labeled as in Fig 3B.

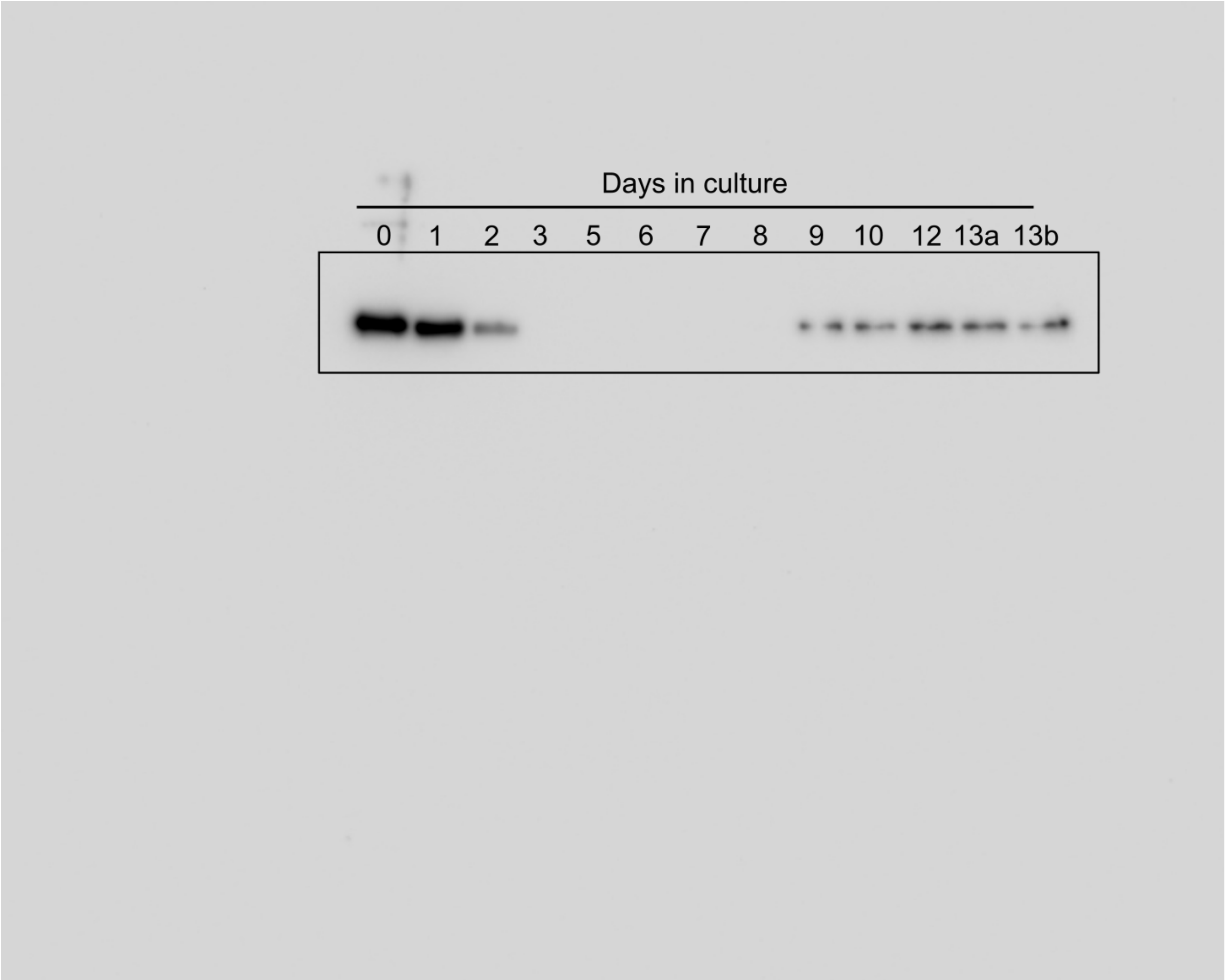

Figure S 19. The area cropped and shown in Fig 3B is indicated by a rectangle. Lanes are labeled as in Fig 3B.

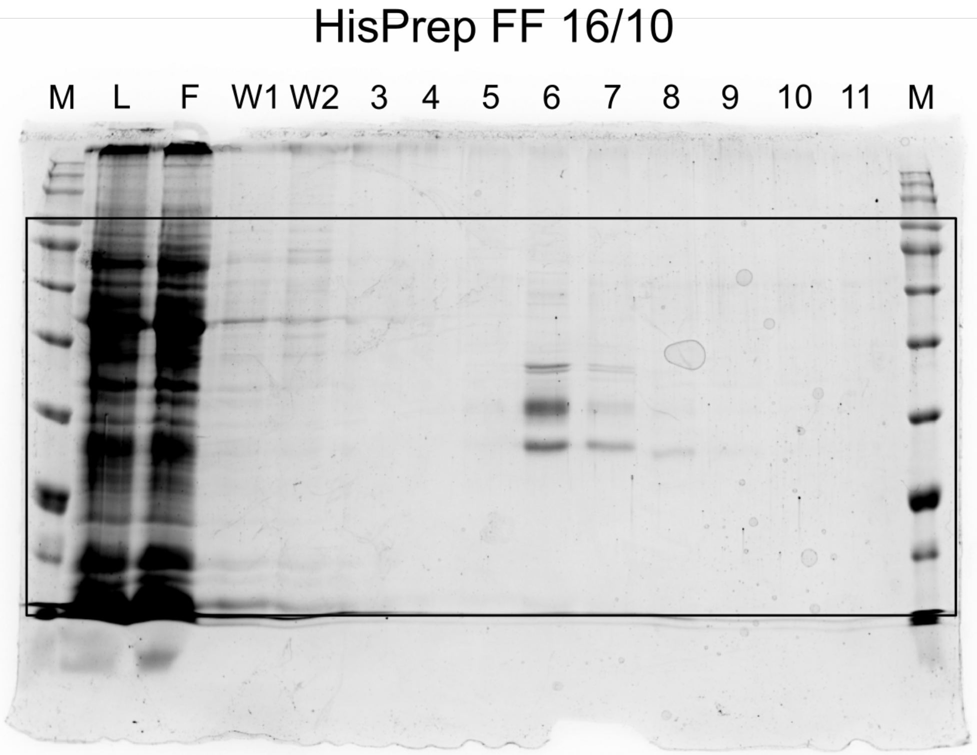

Figure S 20. The area cropped and shown in Fig 4A is indicated by a rectangle. Lanes are labeled as in Fig 4A.

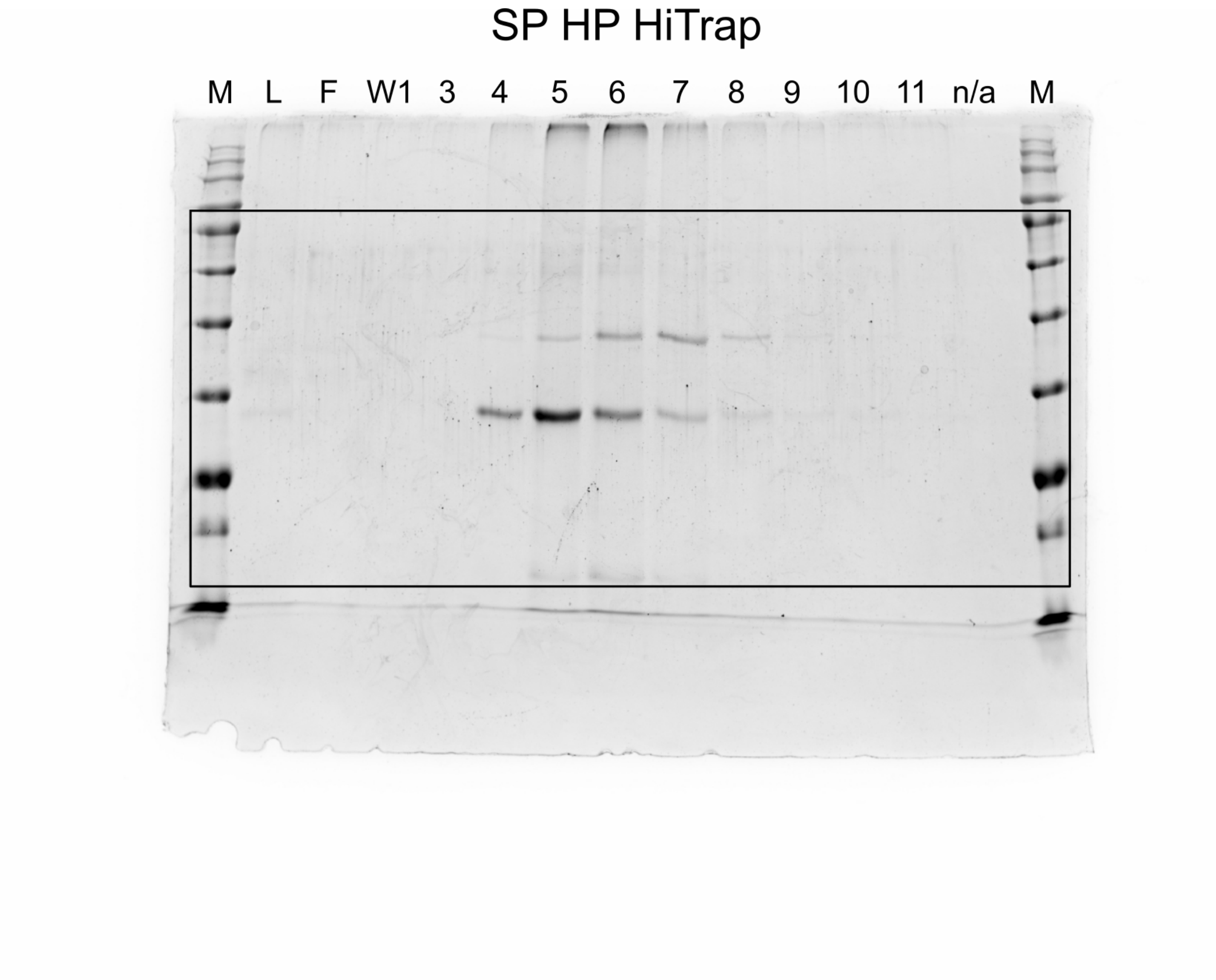

Figure S 21. The area cropped and shown in Fig 4A is indicated by a rectangle. Lanes are labeled as in Fig 4A.

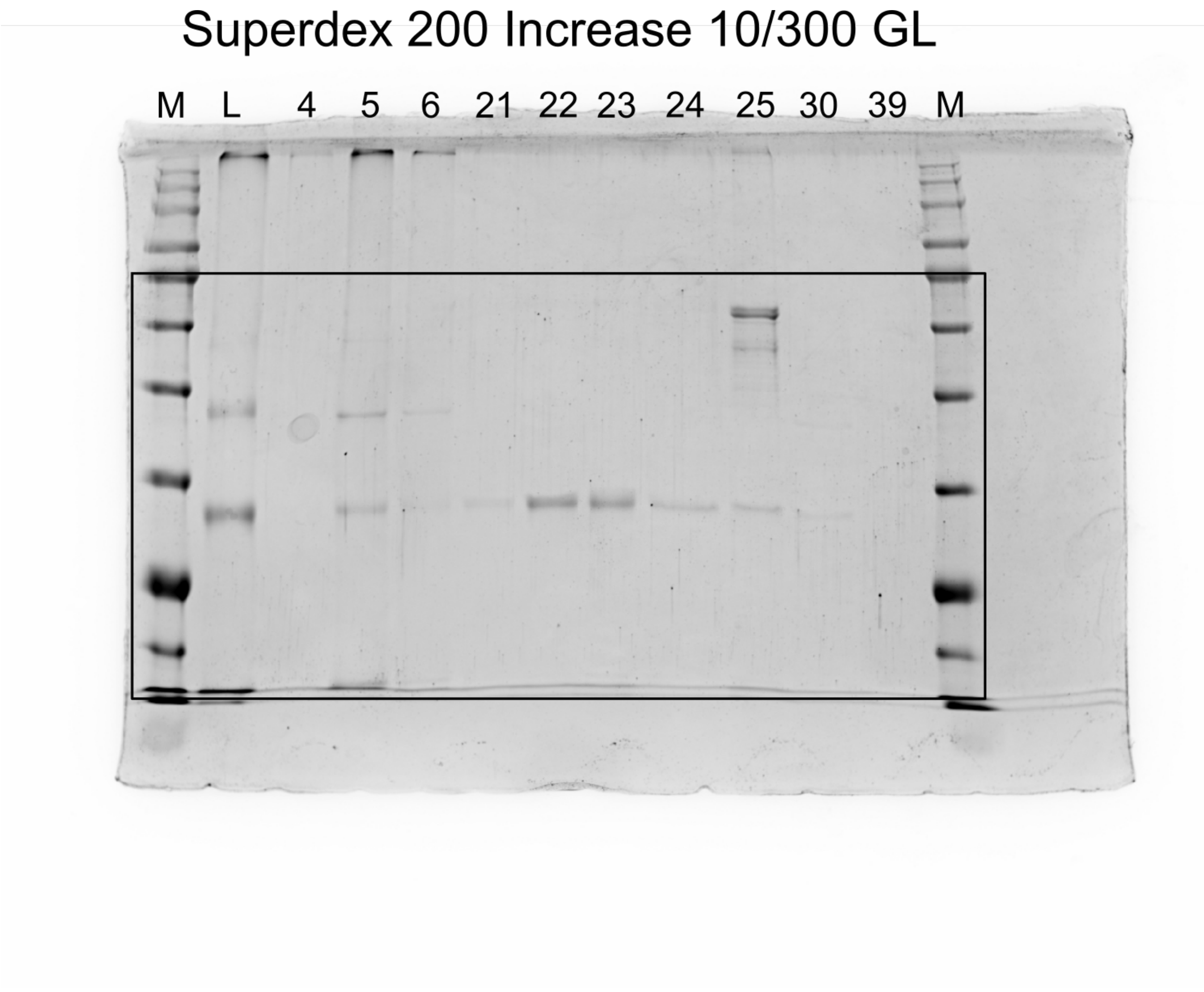

Figure S 22. The area cropped and shown in Fig 4A is indicated by a rectangle. Lanes are labeled as in Fig 4A.

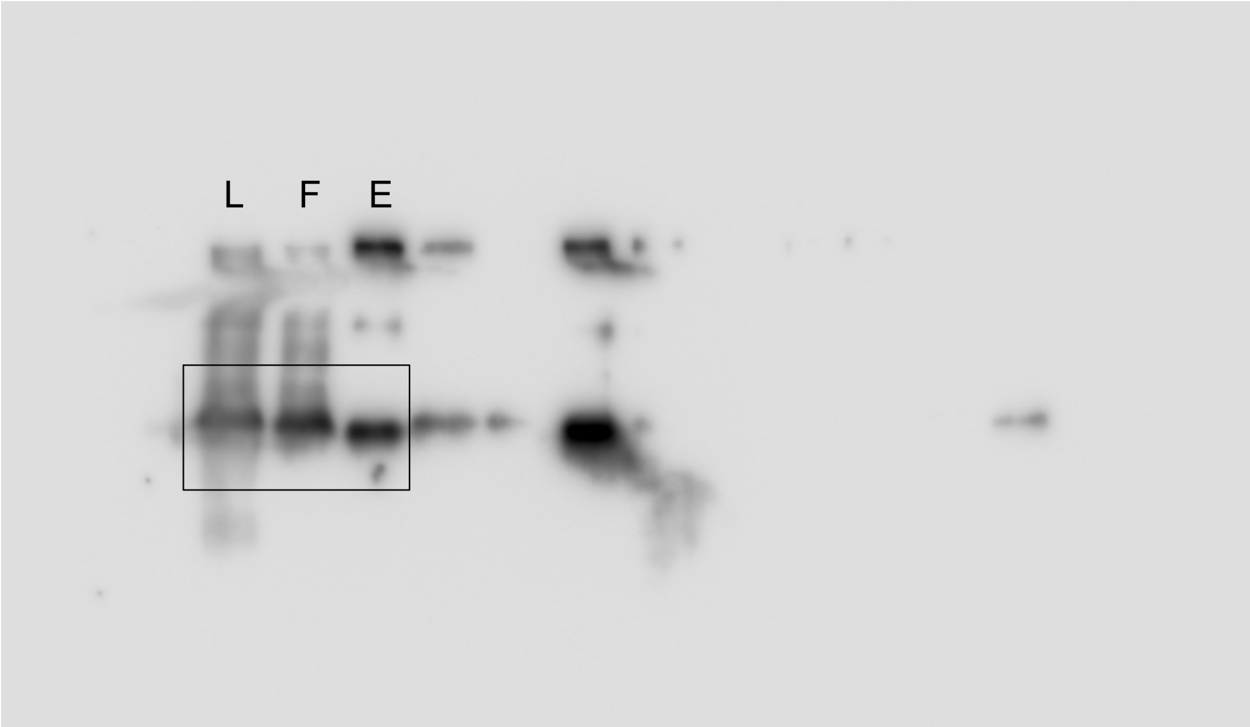

Figure S 23. The area cropped and shown in Fig 4B is indicated by a rectangle. Lanes are labeled as in Fig 4B.

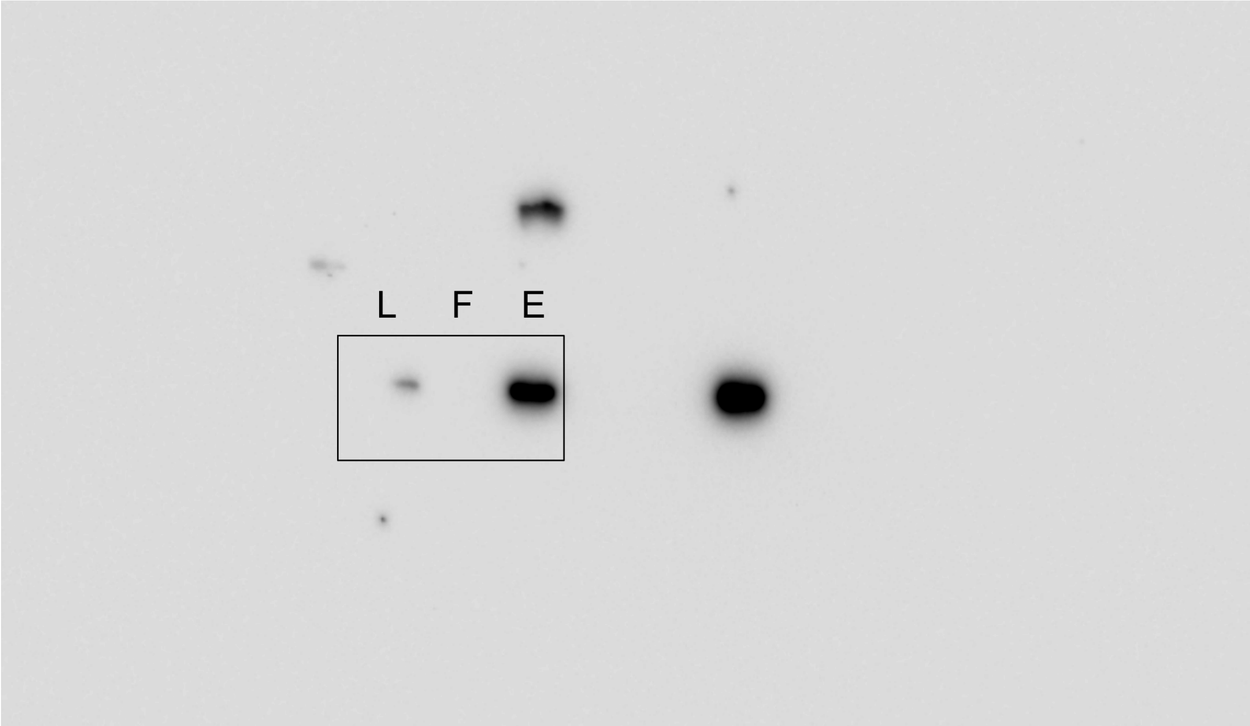

Figure S 24. The area cropped and shown in Fig 4B is indicated by a rectangle. Lanes are labeled as in Fig 4B.

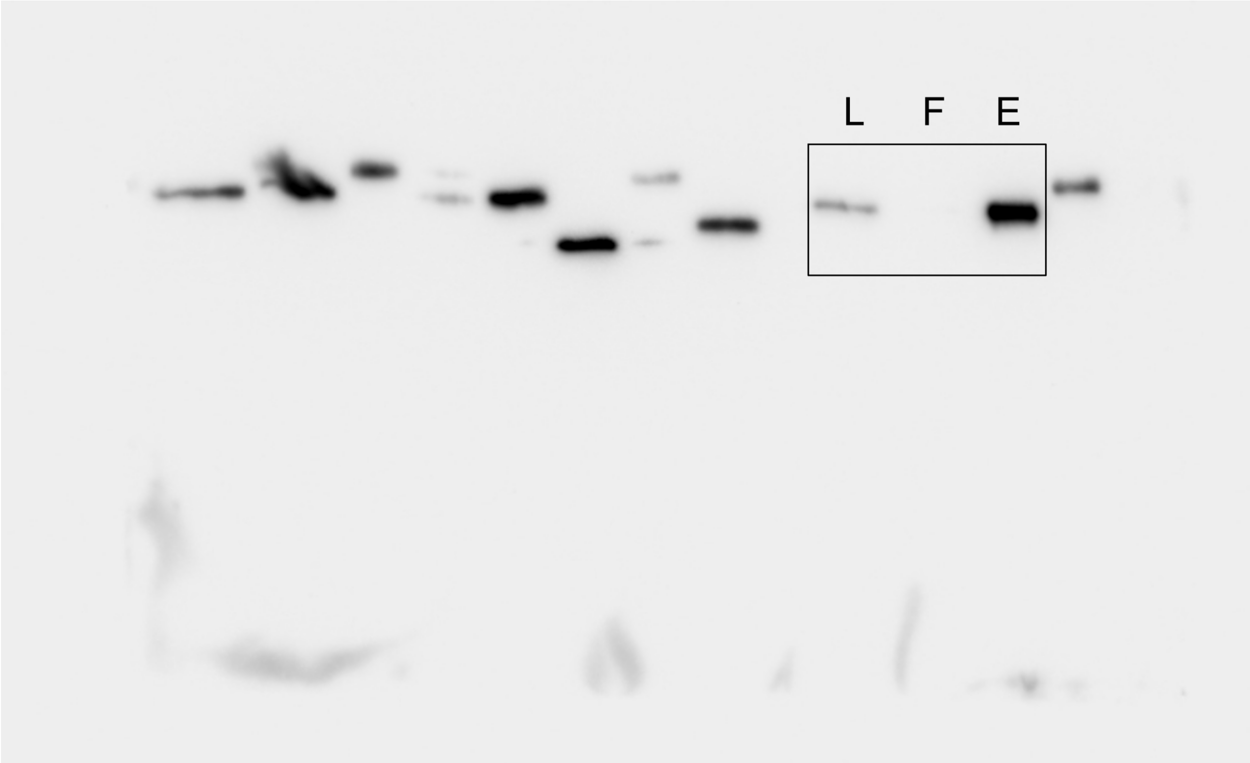

Figure S 25. The area cropped and shown in Fig 4C is indicated by a rectangle. Lanes are labeled as in Fig 4C.

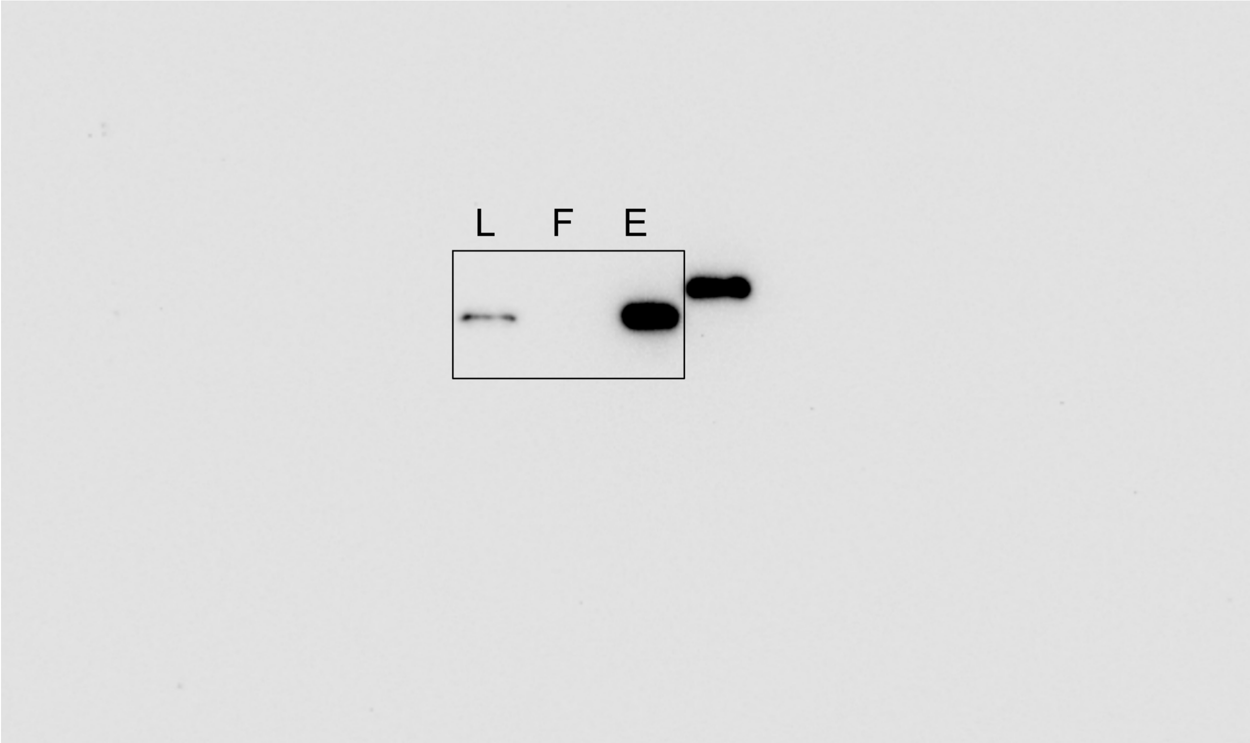

Figure S 26. The area cropped and shown in Fig 4C is indicated by a rectangle. Lanes are labeled as in Fig 4C.

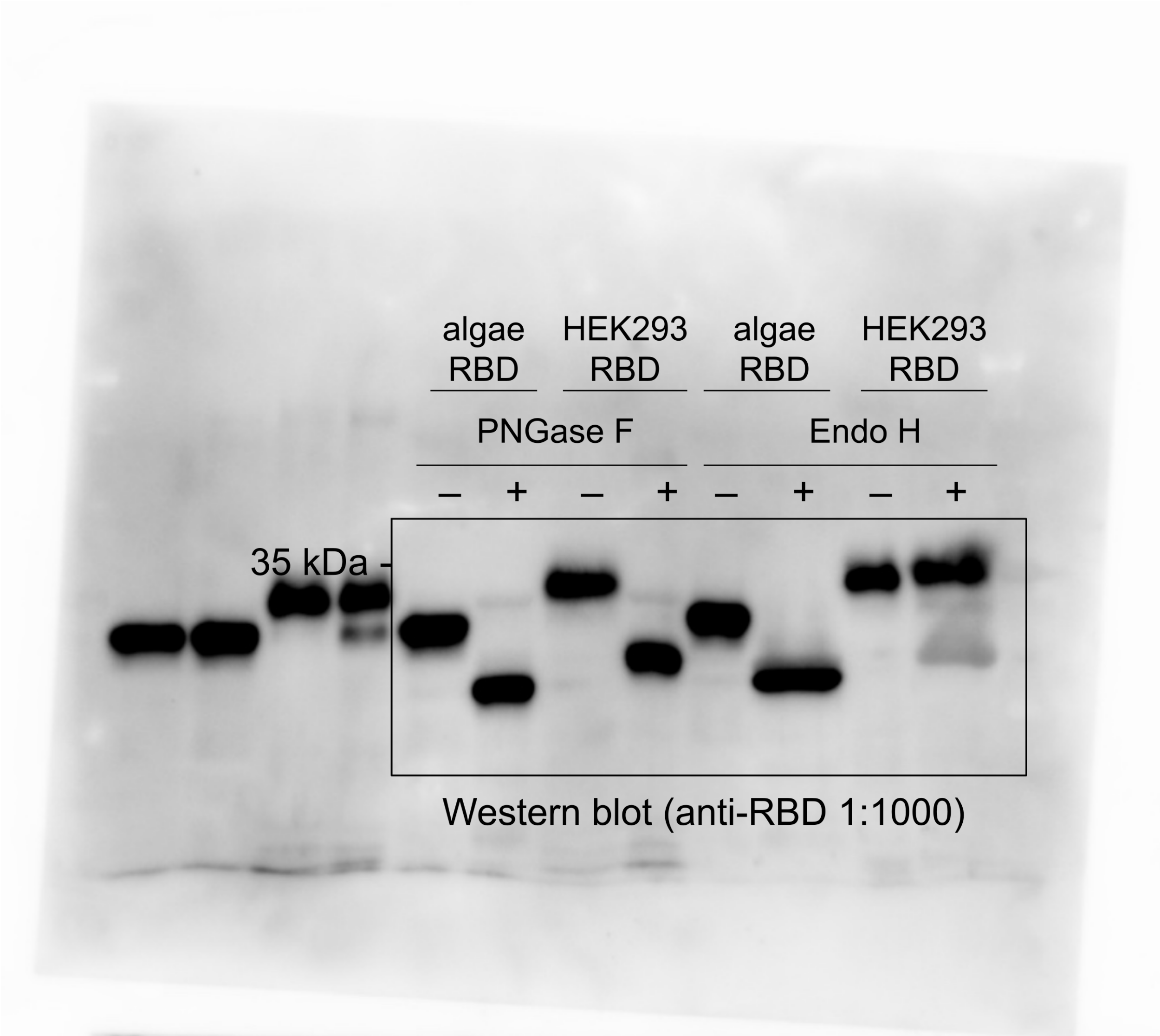

Figure S 27. The area cropped and shown in Fig 4E is indicated by a rectangle. Lanes are labeled as in Fig 4E.

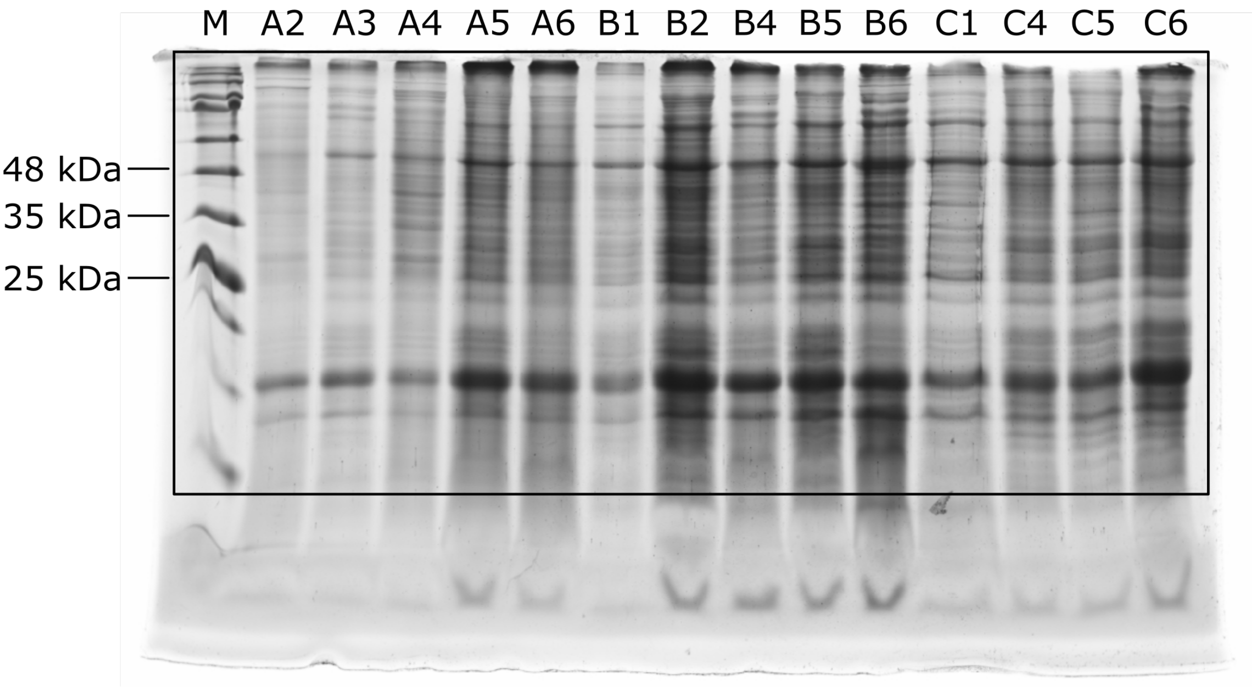

Figure S 28. The area cropped and shown in Supplemental Fig 2A is indicated by a rectangle. Lanes are labeled as in Supplemental Fig 2A.

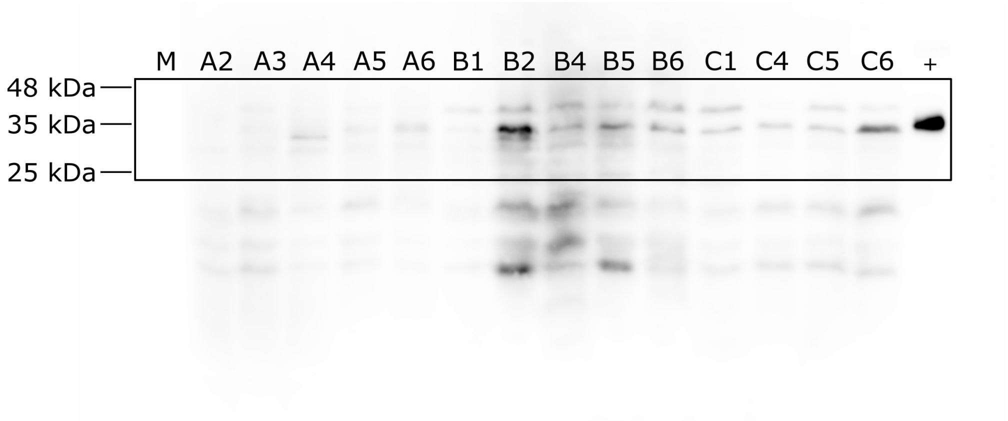

Figure S 29. The area cropped and shown in Supplemental Fig 2A is indicated by a rectangle. Lanes are labeled as in Supplemental Fig 2A.

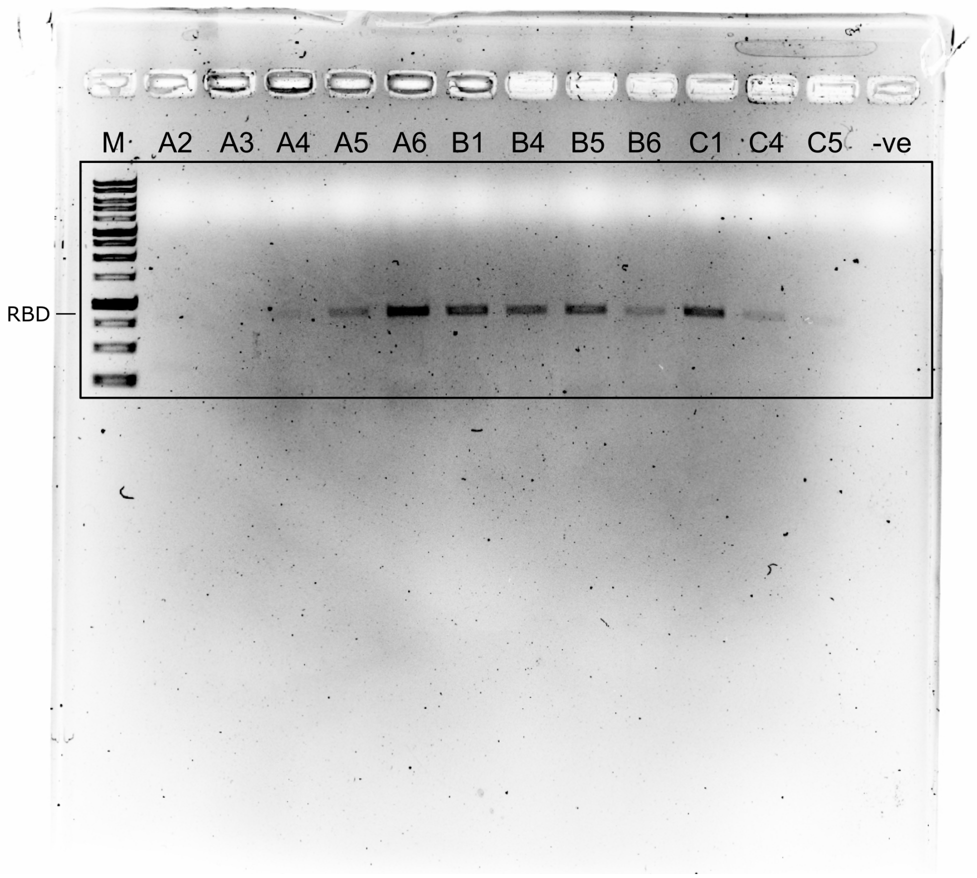

Figure S 30. The area cropped and shown in Supplemental Fig 2B is indicated by a rectangle. Lanes are labeled as in Supplemental Fig 2B.

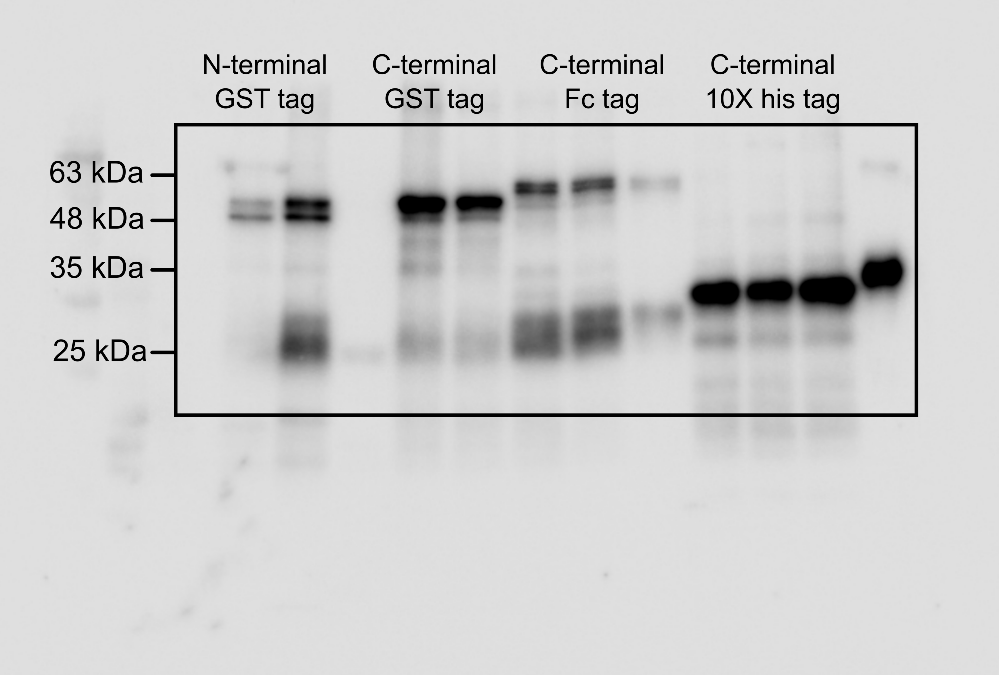

Figure S 31. The area cropped and shown in Supplemental Fig 3 is indicated by a rectangle. Lanes are labeled as in Supplemental Fig 3.

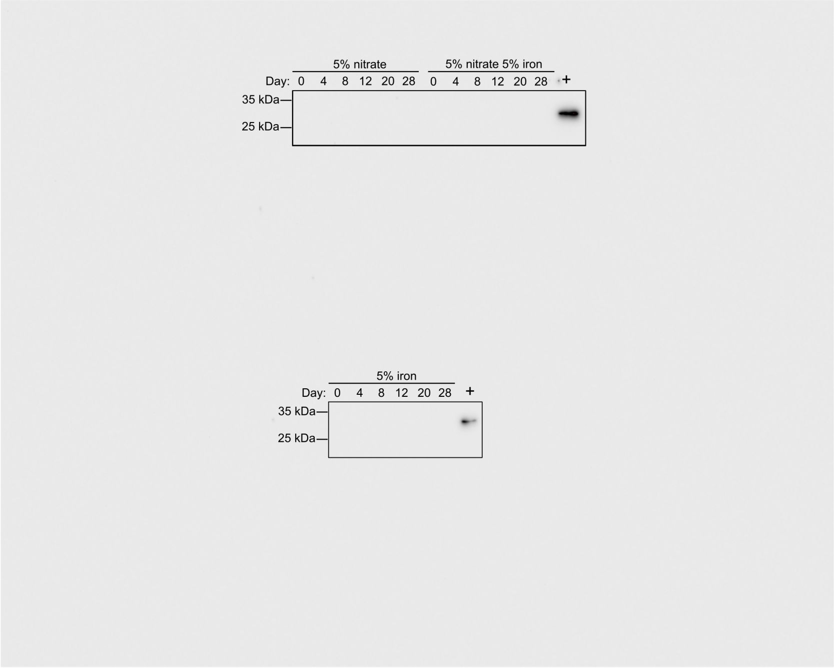

Figure S 32. The area cropped and shown in Supplemental Fig 5B is indicated by a rectangle. Lanes are labeled as in Supplemental Fig 5B.

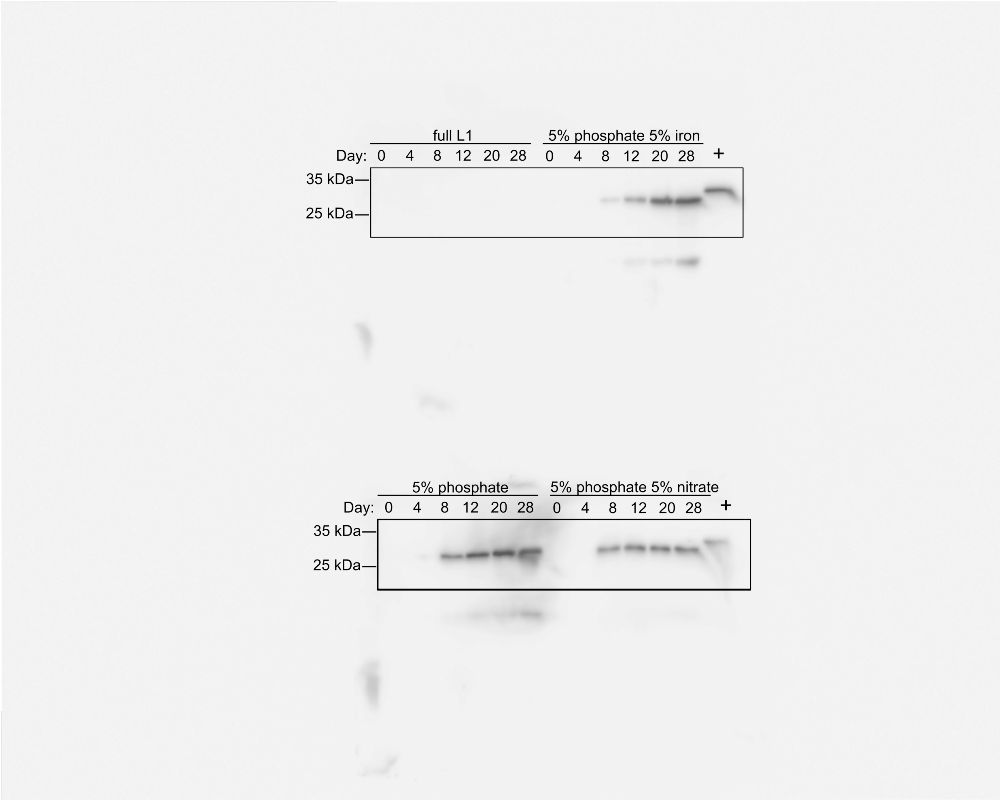

Figure S 33. The area cropped and shown in Supplemental Fig 5B is indicated by a rectangle. Lanes are labeled as in Supplemental Fig 5B.

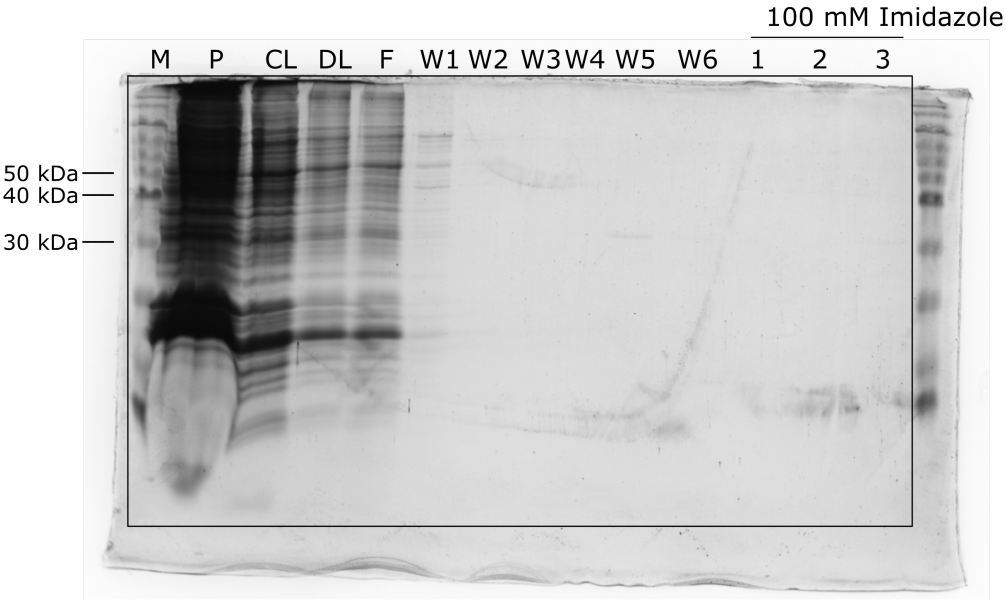

Figure S 34. The area cropped and shown in Supplemental Fig 7 is indicated by a rectangle. Lanes are labeled as in Supplemental Fig 7.

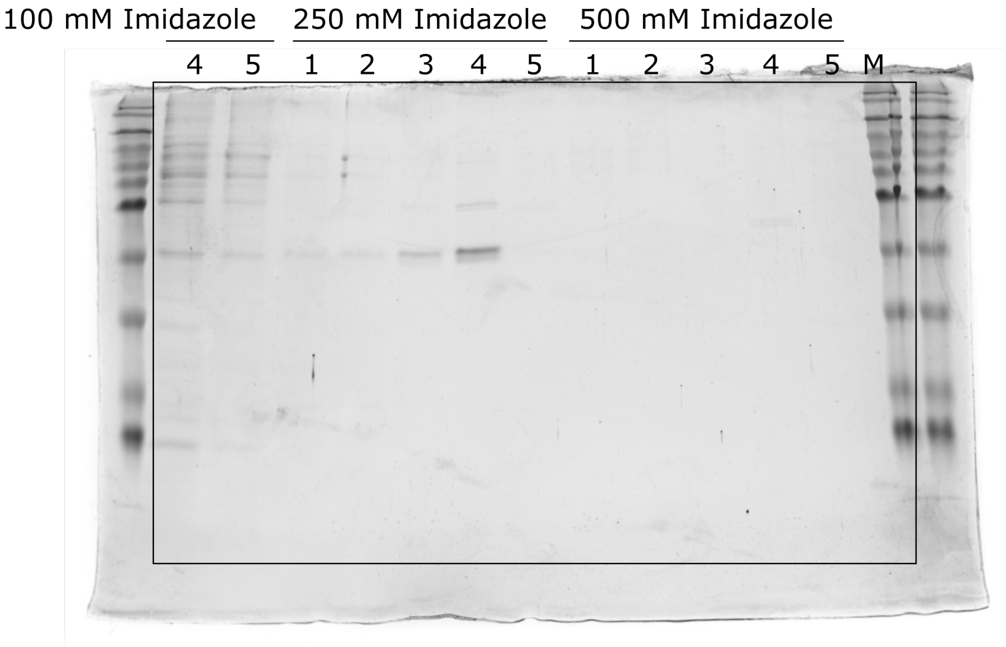

Figure S 35. The area cropped and shown in Supplemental Fig 7 is indicated by a rectangle. Lanes are labeled as in Supplemental Fig 7.

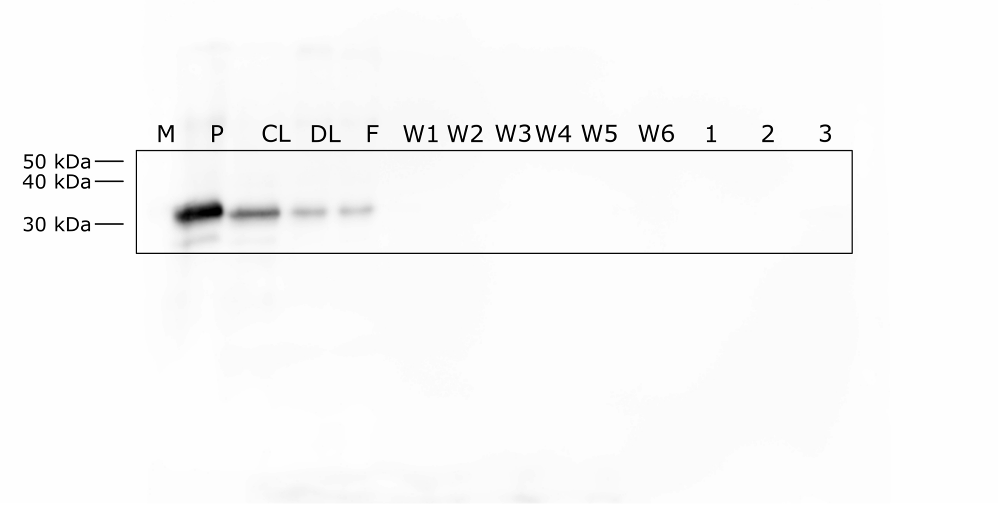

Figure S 36. The area cropped and shown in Supplemental Fig 7 is indicated by a rectangle. Lanes are labeled as in Supplemental Fig 7.

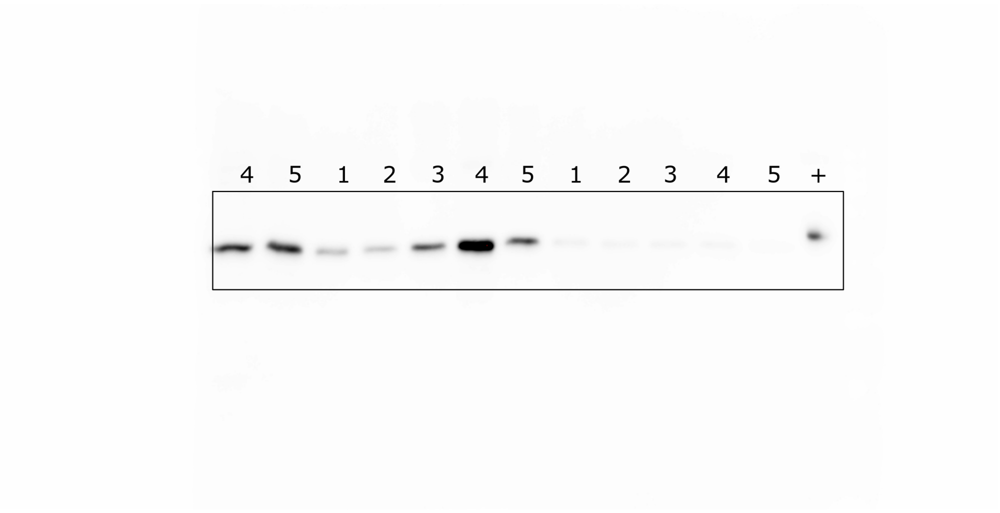

Figure S 37. The area cropped and shown in Supplemental Fig 7 is indicated by a rectangle. Lanes are labeled as in Supplemental Fig 7.

Table S 1. List of plasmids used in this study

Name	Description	Reference or source
pPtGE31	*P. tricornutum* expression vector	Slattery, et al., 2018
pSS1	pPtGE31 with a PtPRA-PH/CH marker, encoding *P. tricornutum* codon optimized RBD protein with a C-terminal 6His tag using the HASP1 promoter (v1) and secretion signal peptide	This study
pSS2	pPtGE31 with a PtPRA-PH/CH marker, encoding *P. tricornutum* codon optimized RBD protein with a C-terminal 6His tag using the HASP1 promoter (v2) and secretion signal peptide (HASP1 v2 is HASP1 v1 with the follow mutations numbered from the first base of the promoter sequence: 43_47del, 402_403insA, 442A>T, 477T>C, 530C>T)	This study
pSS3	pPtGE31 with a PtPRA-PH/CH marker, encoding *P. tricornutum* codon optimized full length Spike protein with a C-terminal 6His tag using the HASP1 promoter (v1) and secretion signal peptide	This study
pSS4	pPtGE31 with a PtPRA-PH/CH marker, encoding *P. tricornutum* codon optimized full length Spike protein with a C-terminal 6His tag using the HASP1 promoter (v2) and secretion signal peptide	This study
pSS5	pPtGE31 with a PtPRA-PH/CH marker, encoding *P. tricornutum* codon optimized full length Spike protein with 2 proline substitutions with a C-terminal 6His tag using the HASP1 promoter (v1) and secretion signal peptide	This study
pSS6	pPtGE31 with a PtPRA-PH/CH marker, encoding *P. tricornutum* codon optimized full length Spike protein with 2 proline substitutions with a C-terminal 6His tag using the HASP1 promoter (v2) and secretion signal peptide	This study
pSS7	pPtGE31 with a PtPRA-PH/CH marker, encoding human codon optimized RBD protein with a C-terminal 6His tag using the 40SRPS8 promoter and native Spike secretion signal peptide	This study
pSS8	pPtGE31 with a PtPRA-PH/CH marker, encoding human codon optimized full length Spike protein with a C-terminal 6His tag using the 40SRPS8 promoter and native Spike secretion signal peptide	This study
pSS10	pPtGE31 with a PtPRA-PH/CH marker, encoding eGFP using the HASP1 promoter (v1) and secretion signal peptide	This study
pSS24	pPtGE31 with a PtPRA-PH/CH marker, encoding *P. tricornutum* codon optimized RBD protein with a C-terminal 10His tag using the HASP1 promoter (v1) and secretion signal peptide	This study
pSS83	pPtGE31 with a PtPRA-PH/CH marker, encoding *P. tricornutum* codon optimized RBD protein with an N-terminal GST tag using the HASP1 promoter (v1) and secretion signal peptide	This study
pSS84	pPtGE31 with a PtPRA-PH/CH marker, encoding *P. tricornutum* codon optimized RBD protein with a C-terminal GST tag using the HASP1 promoter (v1) and secretion signal peptide	This study
pSS86	pPtGE31 with a PtPRA-PH/CH marker, encoding *P. tricornutum* codon optimized RBD protein with a C-terminal Fc tag using the HASP1 promoter (v1) and secretion signal peptide	This study
pTA-Mob	Mobilization helper plasmid required for conjugation	Strand, et al., 2014

Table S 2. Genetic parts used in this study

Name	Sequence (5' to 3')	Description
HASP1 v1	GGATTGATAGTGAAAACCTTATTCATTGTCAGAGCTTAAGCCGGTCT GGTCTATCTTTCCACTGTCAAACAGCTCTTGATTGTCGCCCGCGCGA AAATAGTAGCACTAACTGTAACTTCAAAATACAAAATGTTCTCTGTTA CCATACAGTGAATGTAACTTTCGAATTGACAGTATTAGTAGTCGTATT GACAGTGAGGCACGCCCCTCAATGTGCGAGGTGGAAAATATACCAG CATGACAATGAATCTTGGAGATTCTTTTGCTGTCATCAAGATTCACCG CCAAATCTTCAGGAACCTATCACGTCCACAGGCGATGTTAATTCTTG AGTCGTCAAAACAAAGTCCTGTCCTACCTGTAGAAGTTGACAGCGAG CAATTGTATGCAAACTTCTGACTTTGTTATAATAACATTAAAGGTAATT AAGTATCTTCAATTAGGCATTTTGTCACTGTCAGTCCGTTCCGACAAT ATAGGTAGATTTGGAATGAATCTTTTCTATGCTGCTGCGAATCTTGTA CACCTTTGAGGCCGTAGATTCTGTCCGACGAAGCGATAATTATTGCA AAATACATGGACTCATTATTTTGATTCGATTTCTTTTTGGTATCCGACT CGAAAAGATCCATCACGGCGAGC	*P. tricornutum HASP1* promoter version 1

Table S 2. Genetic parts used in this study

Name	Sequence (5' to 3')	Description
HASP1 v2	GGATTGATAGTGAAAACCTTATTCATTGTCAGAGCTTAAGCCGGTCTA<br>TCTTTCCACTGTCAAACAGCTCTTGATTGTCGCCCGCGCGAAAATAG<br>TAGCACTAACTGTAACTTCAAAATACAAAATGTTCTCTGTTACCATAC<br>AGTGAATGTAACTTTCGAATTGACAGTATTAGTAGTCGTATTGACAGT<br>GAGGCACGCCCCTCAATGTGCGAGGTGGAAAATATACCAGCATGAC<br>AATGAATCTTGGAGATTCTTTTGCTGTCATCAAGATTCACCGCCAAAT<br>CTTCAGGAACCTATCACGTCCACAGGCGATGTTAATTCTTGAGTCGT<br>CAAAACAAAGTCCTGTCCTACCTGTAGAAGTTGACAGCGAGCAATTG<br>TATGCAAACTTCTGACTTATGTTATAATAACATTAAAGGTAATTAAGTA<br>TCTTCAATTTGGCATTTTGTCACTGTCAGTCCGTTCCGACAATACAGG<br>TAGATTTGGAATGAATCTTTTCTATGCTGCTGCGAATCTTGTACACCT<br>TCGAGGCCGTAGATTCTGTCCGACGAAGCGATAATTATTGCAAAATA<br>CATGGACTCATTATTTTGATTCGATTTCTTTTTGGTATCCGACTCGAA<br>AAGATCCATCACGGCGAGC	*P. tricornutum HASP1* promoter version 2
HASP1 secretion signal peptide	ATGAATCTTCGTTGTATCCTTCCGTTTCTCCTCGCAAGCTTCTCGGCT<br>GGGGCT	coding sequence for the *P. tricornutum* HASP1 secretion signal peptide
SARS-CoV-2 native spike secretion signal peptide	ATGTTCGTGTTTCTGGTGCTGCTGCCTCTGGTGTCCAGCCAG	coding sequence for the SARS-CoV-2 Spike glycoprotein secretion signal peptide
P. tricornutum codon optimized RBD	CGTGTGCAGCCCACTGAGTCCATCGTGCGCTTCCCGAATATTACGAA<br>TCTTTGCCCCTTTGGAGAAGTCTTCAATGCCACTCGTTTCGCCTCCG<br>TTTACGCCTGGAACCGTAAGCGCATCTCCAATTGCGTCGCCGATTAC<br>TCCGTTCTTTACAACTCGGCTTCGTTTTCGACTTTCAAATGCTACGGT<br>GTGTCCCCGACTAAGCTCAACGACCTCTGTTTTACGAATGTGTACGC<br>TGATTCCTTTGTCATTCGTGGTGACGAAGTCCGCCAGATCGCTCCGG<br>GTCAGACCGGAAAGATTGCGGACTACAACTACAAACTGCCGGACGA<br>CTTCACTGGATGCGTGATTGCCTGGAATTCCAATAACTTGGATTCGA<br>AAGTTGGAGGTAATTACAATTACTTGTACCGTCTTTTCCGCAAGTCGA<br>ACCTTAAACCCTTTGAACGTGACATTTCCACCGAAATTTACCAAGCCG<br>GCTCGACCCCCTGTAACGGAGTGGAAGGCTTTAATTGCTACTTCCCC<br>CTTCAATCGTACGGATTCCAGCCGACTAACGGTGTTGGATACCAACC<br>CTACCGTGTGGTGGTCCTGTCCTTCGAACTGCTCCACGCTCCGGCG<br>ACTGTCTGTGGACCCAAGAAATCGACCAATCTGGTCAAGAATAAATG<br>TGTCAATTTT	coding sequence for the SARS-CoV-2 Spike glycoprotein receptor binding domain codon optimized for expression in *P. tricornutum*

Table S 2. Genetic parts used in this study

Name	Sequence (5' to 3')	Description
P. tricornutum codon optimized Spike	TGCGTCAATCTGACTACTCGTACCCAGCTTCCGCCCGCTTACACCAA<br>CTCTTTCACCCGAGGCGTTTACTACCCGGACAAAGTTTTCCGTTCCT<br>CGGTCCTTCACTCGACGCAGGACCTTTTTTTGCCGTTCTTTTCGAAC<br>GTCACTTGGTTCCACGCGATTCACGTTTCCGGCACGAATGGTACGAA<br>ACGTTTTGATAATCCCGTTCTGCCCTTTAATGACGGTGTCTACTTCGC<br>GTCCACTGAAAAATCGAACATCATCCGTGGCTGGATCTTTGGCACTA<br>CGCTCGATTCCAAAACCCAATCGCTGCTCATCGTTAATAATGCCACC<br>AATGTCGTTATTAAAGTCTGCGAGTTCCAATTTTGTAATGACCCGTTT<br>CTTGGTGTGTACTACCACAAGAACAACAAGTCGTGGATGGAGTCCG<br>AGTTCCGTGTGTACTCCTCCGCCAACAACTGTACTTTTGAATACGTTT<br>CGCAACCCTTTCTTATGGATTTGGAAGGCAAACAGGGCAACTTTAAA<br>AATCTGCGTGAATTTGTCTTCAAGAATATTGACGGTTACTTCAAAATT<br>TACTCCAAACACACTCCGATTAACTTGGTGCGCGATCTTCCCCAAGG<br>TTTCTCCGCGCTCGAACCGCTGGTCGACCTTCCGATCGGTATCAACA<br>TTACCCGCTTCCAGACTCTCCTGGCGCTTCACCGTTCGTACCTGACT<br>CCCGGTGATTCGTCCTCGGGTTGGACGGCGGGTGCGGCGGCTTAC<br>TACGTTGGCTACCTTCAACCGCGCACGTTTTTGTTGAAGTACAACGA<br>GAACGGTACCATCACTGATGCGGTGGACTGCGCTTTGGACCCGCTT<br>TCCGAGACCAAATGTACTCTTAAGTCCTTTACGGTCGAGAAAGGTAT<br>CTACCAAACTTCTAATTTCCGTGTGCAGCCCACTGAGTCCATCGTGC<br>GCTTCCCGAATATTACGAATCTTTGCCCCTTTGGAGAAGTCTTCAATG<br>CCACTCGTTTCGCCTCCGTTTACGCCTGGAACCGTAAGCGCATCTCC<br>AATTGCGTCGCCGATTACTCCGTTCTTTACAACTCGGCTTCGTTTTCG<br>ACTTTCAAATGCTACGGTGTGTCCCCGACTAAGCTCAACGACCTCTG<br>TTTTACGAATGTGTACGCTGATTCCTTTGTCATTCGTGGTGACGAAGT<br>CCGCCAGATCGCTCCGGGTCAGACCGGAAAGATTGCGGACTACAAC<br>TACAAACTGCCGGACGACTTCACTGGATGCGTGATTGCCTGGAATTC<br>CAATAACTTGGATTCGAAAGTTGGAGGTAATTACAATTACTTGTACCG<br>TCTTTTCCGCAAGTCGAACCTTAAACCCTTTGAACGTGACATTTCCAC<br>CGAAATTTACCAAGCCGGCTCGACCCCCTGTAACGGAGTGGAAGGC<br>TTTAATTGCTACTTCCCCCTTCAATCGTACGGATTCCAGCCGACTAAC<br>GGTGTTGGATACCAACCCTACCGTGTGGTGGTCCTGTCCTTCGAACT<br>GCTCCACGCTCCGGCGACTGTCTGTGGACCCAAGAAATCGACCAAT	coding sequence for the SARS-CoV-2 Spike glycoprotein codon optimized for expression in *P. tricornutum*

Table S 2. Genetic parts used in this study

Name	Sequence (5’ to 3’)	Description
	CTGGTCAAGAATAAATGTGTCAATTTTAATTTTAACGGTTTGACGGGA ACGGGTGTGCTCACCGAGTCCAACAAAAAGTTCCTTCCCTTCCAGCA GTTTGGTCGCGATATTGCGGATACCACGGACGCGGTGCGTGATCCG CAAACCCTGGAAATTTTGGATATTACTCCCTGCTCCTTTGGTGGTGTT TCCGTCATTACCCCGGGCACCAATACTTCGAATCAGGTTGCGGTCCT CTACCAGGACGTGAATTGCACTGAAGTCCCCGTCGCCATTCACGCC GACCAGCTTACTCCGACGTGGCGCGTGTACTCCACCGGCTCCAATG TTTTTCAAACCCGTGCGGGCTGCCTGATCGGCGCCGAGCACGTTAA CAATTCCTACGAGTGCGACATCCCCATCGGAGCCGGCATCTGCGCT TCGTACCAGACTCAAACGAATTCGCCGCGTCGCGCCCGTTCCGTGG CGTCCCAGTCGATTATTGCGTACACCATGTCCCTGGGAGCGGAAAA CTCCGTTGCCTACTCGAATAATTCGATTGCGATCCCCACCAACTTCA CTATCTCGGTCACTACTGAGATTTTGCCCGTTTCCATGACTAAGACC TCCGTGGATTGCACCATGTACATTTGCGGTGACTCCACGGAATGCTC CAACCTGCTTCTGCAGTACGGTTCGTTTTGCACCCAGTTGAATCGCG CGCTGACCGGAATTGCCGTGGAACAAGACAAAAACACTCAGGAGGT TTTCGCCCAAGTTAAGCAGATCTACAAGACGCCCCCCATCAAAGACT TCGGCGGATTCAACTTCTCCCAGATCTTGCCCGATCCCTCGAAACCG TCGAAGCGTTCGTTTATCGAAGATCTCCTTTTCAACAAAGTGACGCTT GCGGATGCTGGTTTCATCAAACAATACGGTGACTGCTTGGGAGACAT TGCCGCTCGTGACCTCATTTGCGCGCAAAAGTTTAATGGCCTCACTG TGCTGCCCCCGCTGTTGACGGATGAAATGATTGCTCAGTACACGTCC GCGCTTCTGGCGGGAACTATTACGTCGGGCTGGACGTTTGGTGCTG GCGCGGCGCTCCAAATCCCCTTCGCGATGCAAATGGCCTACCGTTT CAACGGTATCGGAGTGACTCAAAACGTCTTGTACGAGAATCAGAAGC TGATCGCTAACCAGTTTAATTCCGCGATTGGTAAGATTCAAGATTCGC TTTCCTCGACGGCTTCCGCTCTTGGCAAGCTGCAAGACGTCGTTAAC CAAAACGCTCAAGCTCTTAACACCCTGGTTAAGCAACTGTCGTCCAA CTTTGGCGCCATTTCGTCGGTGCTTAACGACATTCTCTCCCGTCTTG ATAAAGTCGAGGCTGAAGTGCAGATCGATCGTCTCATCACTGGTCGT CTTCAGTCGCTTCAGACGTACGTCACCCAACAGCTTATCCGTGCTGC TGAAATCCGTGCGTCGGCTAATCTTGCGGCTACGAAGATGTCCGAGT GCGTCCTGGGCCAGTCCAAGCGTGTCGACTTCTGTGGTAAAGGTTA CCACCTCATGTCGTTTCCCCAATCGGCTCCGCACGGTGTGGTGTTCC TCCACGTGACCTACGTGCCGGCCCAAGAGAAAAACTTTACGACGGC GCCCGCTATTTGCCACGACGGTAAAGCGCACTTCCCGCGTGAGGGA GTTTTCGTTTCCAACGGCACCCACTGGTTCGTGACCCAACGTAATTT TTACGAGCCGCAAATCATCACTACGGACAATACTTTCGTGTCCGGAA ACTGCGACGTTGTTATTGGCATTGTCAACAATACCGTGTACGATCCG CTGCAGCCGGAGCTTGACTCCTTCAAGGAAGAATTGGACAAATACTT TAAGAATCACACCTCGCCCGATGTGGACTTGGGCGACATCTCCGGA ATTAATGCGTCCGTTGTTAACATTCAGAAGGAGATCGACCGTCTGAA CGAAGTGGCGAAGAACCTCAACGAATCCCTGATTGACCTGCAAGAG TTGGGAAAATACGAGCAATACATTAAATGGCCGTGGTACATCTGGCT GGGATTTATTGCTGGCCTTATCGCTATCGTCATGGTGACCATTATGC TCTGTTGCATGACTTCGTGTTGCTCGTGTCTTAAGGGCTGCTGCTCC TGTGGATCCTGCTGCAAATTTGACGAAGACGACTCGGAACCGGTGC TGAAAGGTGTCAAACTGCACTACACT	

Table S 2. Genetic parts used in this study

Name	Sequence (5' to 3')	Description
P. tricornutum codon optimized Spike 2P	TGCGTCAATCTGACTACTCGTACCCAGCTTCCGCCCGCTTACACCAA CTCTTTCACCCGAGGCGTTTACTACCCGGACAAAGTTTTCCGTTCCT CGGTCCTTCACTCGACGCAGGACCTTTTTTTGCCGTTCTTTTCGAAC GTCACTTGGTTCCACGCGATTCACGTTTCCGGCACGAATGGTACGAA ACGTTTTGATAATCCCGTTCTGCCCTTTAATGACGGTGTCTACTTCGC GTCCACTGAAAAATCGAACATCATCCGTGGCTGGATCTTTGGCACTA CGCTCGATTCCAAAACCCAATCGCTGCTCATCGTTAATAATGCCACC AATGTCGTTATTAAAGTCTGCGAGTTCCAATTTTGTAATGACCCGTTT CTTGGTGTGTACTACCACAAGAACAACAAGTCGTGGATGGAGTCCG AGTTCCGTGTGTACTCCTCCGCCAACAACTGTACTTTTGAATACGTTT CGCAACCCTTTCTTATGGATTTGGAAGGCAAACAGGGCAACTTTAAA AATCTGCGTGAATTTGTCTTCAAGAATATTGACGGTTACTTCAAAATT TACTCCAAACACACTCCGATTAACTTGGTGCGCGATCTTCCCCAAGG TTTCTCCGCGCTCGAACCGCTGGTCGACCTTCCGATCGGTATCAACA TTACCCGCTTCCAGACTCTCCTGGCGCTTCACCGTTCGTACCTGACT CCCGGTGATTCGTCCTCGGGTTGGACGGCGGGTGCGGCGGCTTAC TACGTTGGCTACCTTCAACCGCGCACGTTTTTGTTGAAGTACAACGA GAACGGTACCATCACTGATGCGGTGGACTGCGCTTTGGACCCGCTT TCCGAGACCAAATGTACTCTTAAGTCCTTTACGGTCGAGAAAGGTAT CTACCAAACTTCTAATTTCCGTGTGCAGCCCACTGAGTCCATCGTGC GCTTCCCGAATATTACGAATCTTTGCCCCTTTGGAGAAGTCTTCAATG CCACTCGTTTCGCCTCCGTTTACGCCTGGAACCGTAAGCGCATCTCC AATTGCGTCGCCGATTACTCCGTTCTTTACAACTCGGCTTCGTTTTCG ACTTTCAAATGCTACGGTGTGTCCCCGACTAAGCTCAACGACCTCTG TTTTACGAATGTGTACGCTGATTCCTTTGTCATTCGTGGTGACGAAGT CCGCCAGATCGCTCCGGGTCAGACCGGAAAGATTGCGGACTACAAC TACAAACTGCCGGACGACTTCACTGGATGCGTGATTGCCTGGAATTC CAATAACTTGGATTCGAAAGTTGGAGGTAATTACAATTACTTGTACCG TCTTTTCCGCAAGTCGAACCTTAAACCCTTTGAACGTGACATTTCCAC CGAAATTTACCAAGCCGGCTCGACCCCCTGTAACGGAGTGGAAGGC TTTAATTGCTACTTCCCCCTTCAATCGTACGGATTCCAGCCGACTAAC GGTGTTGGATACCAACCCTACCGTGTGGTGGTCCTGTCCTTCGAACT GCTCCACGCTCCGGCGACTGTCTGTGGACCCAAGAAATCGACCAAT CTGGTCAAGAATAAATGTGTCAATTTTAATTTTAACGGTTTGACGGGA ACGGGTGTGCTCACCGAGTCCAACAAAAAGTTCCTTCCCTTCCAGCA GTTTGGTCGCGATATTGCGGATACCACGGACGCGGTGCGTGATCCG CAAACCCTGGAAATTTTGGATATTACTCCCTGCTCCTTTGGTGGTGTT TCCGTCATTACCCCGGGCACCAATACTTCGAATCAGGTTGCGGTCCT CTACCAGGACGTGAATTGCACTGAAGTCCCCGTCGCCATTCACGCC GACCAGCTTACTCCGACGTGGCGCGTGTACTCCACCGGCTCCAATG TTTTTCAAACCCGTGCGGGCTGCCTGATCGGCGCCGAGCACGTTAA CAATTCCTACGAGTGCGACATCCCCATCGGAGCCGGCATCTGCGCT TCGTACCAGACTCAAACGAATTCGCCGCGTCGCGCCCGTTCCGTGG CGTCCCAGTCGATTATTGCGTACACCATGTCCCTGGGAGCGGAAAA CTCCGTTGCCTACTCGAATAATTCGATTGCGATCCCCACCAACTTCA CTATCTCGGTCACTACTGAGATTTTGCCCGTTTCCATGACTAAGACCT CCGTGGATTGCACCATGTACATTTGCGGTGACTCCACGGAATGCTCC AACCTGCTTCTGCAGTACGGTTCGTTTTGCACCCAGTTGAATCGCGC GCTGACCGGAATTGCCGTGGAACAAGACAAAAACACT	coding sequence for the SARS-CoV-2 Spike glycoprotein with 2 proline substitutions codon optimized for expression in P. tricornutum

Table S 2. Genetic parts used in this study

Name	Sequence (5’ to 3’)	Description
	CAGGAGGTTTTCGCCCAAGTTAAGCAGATCTACAAGACGCCCCCCAT<br>CAAAGACTTCGGCGGATTCAACTTCTCCCAGATCTTGCCCGATCCCT<br>CGAAACCGTCGAAGCGTTCGTTTATCGAAGATCTCCTTTTCAACAAA<br>GTGACGCTTGCGGATGCTGGTTTCATCAAACAATACGGTGACTGCTT<br>GGGAGACATTGCCGCTCGTGACCTCATTTGCGCGCAAAAGTTTAATG<br>GCCTCACTGTGCTGCCCCCGCTGTTGACGGATGAAATGATTGCTCA<br>GTACACGTCCGCGCTTCTGGCGGGAACTATTACGTCGGGCTGGACG<br>TTTGGTGCTGGCGCGGCGCTCCAAATCCCCTTCGCGATGCAAATGG<br>CCTACCGTTTCAACGGTATCGGAGTGACTCAAAACGTCTTGTACGAG<br>AATCAGAAGCTGATCGCTAACCAGTTTAATTCCGCGATTGGTAAGATT<br>CAAGATTCGCTTTCCTCGACGGCTTCCGCTCTTGGCAAGCTGCAAGA<br>CGTCGTTAACCAAAACGCTCAAGCTCTTAACACCCTGGTTAAGCAAC<br>TGTCGTCCAACTTTGGCGCCATTTCGTCGGTGCTTAACGACATTCTC<br>TCCCGTCTTGATCCGCCGGAGGCTGAAGTGCAGATCGATCGTCTCA<br>TCACTGGTCGTCTTCAGTCGCTTCAGACGTACGTCACCCAACAGCTT<br>ATCCGTGCTGCTGAAATCCGTGCGTCGGCTAATCTTGCGGCTACGAA<br>GATGTCCGAGTGCGTCCTGGGCCAGTCCAAGCGTGTCGACTTCTGT<br>GGTAAAGGTTACCACCTCATGTCGTTTCCCCAATCGGCTCCGCACGG<br>TGTGGTGTTCCTCCACGTGACCTACGTGCCGGCCCAAGAGAAAAAC<br>TTTACGACGGCGCCCGCTATTTGCCACGACGGTAAAGCGCACTTCC<br>CGCGTGAGGGAGTTTTCGTTTCCAACGGCACCCACTGGTTCGTGAC<br>CCAACGTAATTTTTACGAGCCGCAAATCATCACTACGGACAATACTTT<br>CGTGTCCGGAAACTGCGACGTTGTTATTGGCATTGTCAACAATACCG<br>TGTACGATCCGCTGCAGCCGGAGCTTGACTCCTTCAAGGAAGAATTG<br>GACAAATACTTTAAGAATCACACCTCGCCCGATGTGGACTTGGGCGA<br>CATCTCCGGAATTAATGCGTCCGTTGTTAACATTCAGAAGGAGATCG<br>ACCGTCTGAACGAAGTGGCGAAGAACCTCAACGAATCCCTGATTGAC<br>CTGCAAGAGTTGGGAAAATACGAGCAATACATTAAATGGCCGTGGTA<br>CATCTGGCTGGGATTTATTGCTGGCCTTATCGCTATCGTCATGGTGA<br>CCATTGTGCTCTGTTGCATGACTTCGTGTTGCTCGTGTCTTAAGGGC<br>TGCTGCTCCTGTGGATCCTGCTGCAAATTTGACGAAGACGACTCGGA<br>ACCGGTGCTGAAAGGTGTCAAACTGCACTACACT	
Human codon optimized RBD	CGGGTGCAGCCCACCGAATCCATCGTGCGGTTCCCCAATATCACCA<br>ATCTGTGCCCCTTCGGCGAGGTGTTCAATGCCACCAGATTCGCCTCT<br>GTGTACGCCTGGAACCGGAAGCGGATCAGCAATTGCGTGGCCGACT<br>ACTCCGTGCTGTACAACTCCGCCAGCTTCAGCACCTTCAAGTGCTAC<br>GGCGTGTCCCCTACCAAGCTGAACGACCTGTGCTTCACAAACGTGT<br>ACGCCGACAGCTTCGTGATCCGGGGAGATGAAGTGCGGCAGATTGC<br>CCCTGGACAGACAGGCAAGATCGCCGACTACAACTACAAGCTGCCC<br>GACGACTTCACCGGCTGTGTGATTGCCTGGAACAGCAACAACCTGG<br>ACTCCAAAGTCGGCGGCAACTACAATTACCTGTACCGGCTGTTCCGG<br>AAGTCCAATCTGAAGCCCTTCGAGCGGGACATCTCCACCGAGATCTA<br>TCAGGCCGGCAGCACCCCTTGTAACGGCGTGGAAGGCTTCAACTGC<br>TACTTCCCACTGCAGTCCTACGGCTTTCAGCCCACAAATGGCGTGGG<br>CTATCAGCCCTACAGAGTGGTGGTGCTGAGCTTCGAACTGCTGCAT<br>GCCCCTGCCACAGTGTGCGGCCCTAAGAAAAGCACCAATCTCGTGA<br>AGAACAAATGCGTGAACTTC	coding sequence for the SARS-CoV-2 Spike glycoprotein receptor binding domain

Table S 2. Genetic parts used in this study

Name	Sequence (5' to 3')	Description
Human codon optimized Spike	TGTGTGAACCTGACCACAAGAACCCAGCTGCCTCCAGCCTACACCAA<br>CAGCTTTACCAGAGGCGTGTACTACCCCGACAAGGTGTTCAGATCCA<br>GCGTGCTGCACTCTACCCAGGACCTGTTCCTGCCTTTCTTCAGCAAC<br>GTGACCTGGTTCCACGCCATCCACGTGTCCGGCACCAATGGCACCA<br>AGAGATTCGACAACCCCGTGCTGCCCTTCAACGACGGGGTGTACTTT<br>GCCAGCACCGAGAAGTCCAACATCATCAGAGGCTGGATCTTCGGCA<br>CCACACTGGACAGCAAGACCCAGAGCCTGCTGATCGTGAACAACGC<br>CACCAACGTGGTCATCAAAGTGTGCGAGTTCCAGTTCTGCAACGACC<br>CCTTCCTGGGCGTCTACTATCACAAGAACAACAAGAGCTGGATGGAA<br>AGCGAGTTCCGGGTGTACAGCAGCGCCAACAACTGCACCTTCGAGT<br>ACGTGTCCCAGCCTTTCCTGATGGACCTGGAAGGCAAGCAGGGCAA<br>CTTCAAGAACCTGCGCGAGTTCGTGTTCAAGAACATCGACGGCTACT<br>TCAAGATCTACAGCAAGCACACCCCTATCAACCTCGTGCGGGATCTG<br>CCTCAGGGCTTCTCTGCTCTGGAACCCCTGGTGGATCTGCCCATCG<br>GCATCAACATCACCCGGTTTCAGACACTGCTGGCCCTGCACAGAAG<br>CTACCTGACACCTGGCGATAGCAGCAGCGGATGGACAGCTGGTGCC<br>GCCGCTTACTATGTGGGCTACCTGCAGCCTAGAACCTTTCTGCTGAA<br>GTACAACGAGAACGGCACCATCACCGACGCCGTGGATTGTGCTCTG<br>GATCCTCTGAGCGAGACAAAGTGCACCCTGAAGTCCTTCACCGTGG<br>AAAAGGGCATCTACCAGACCAGCAACTTCCGGGTGCAGCCCACCGA<br>ATCCATCGTGCGGTTCCCCAATATCACCAATCTGTGCCCCTTCGGCG<br>AGGTGTTCAATGCCACCAGATTCGCCTCTGTGTACGCCTGGAACCG<br>GAAGCGGATCAGCAATTGCGTGGCCGACTACTCCGTGCTGTACAAC<br>TCCGCCAGCTTCAGCACCTTCAAGTGCTACGGCGTGTCCCCTACCAA<br>GCTGAACGACCTGTGCTTCACAAACGTGTACGCCGACAGCTTCGTG<br>ATCCGGGGAGATGAAGTGCGGCAGATTGCCCCTGGACAGACAGGCA<br>AGATCGCCGACTACAACTACAAGCTGCCCGACGACTTCACCGGCTG<br>TGTGATTGCCTGGAACAGCAACAACCTGGACTCCAAAGTCGGCGGC<br>AACTACAATTACCTGTACCGGCTGTTCCGGAAGTCCAATCTGAAGCC<br>CTTCGAGCGGGACATCTCCACCGAGATCTATCAGGCCGGCAGCACC<br>CCTTGTAACGGCGTGGAAGGCTTCAACTGCTACTTCCCACTGCAGTC<br>CTACGGCTTTCAGCCCACAAATGGCGTGGGCTATCAGCCCTACAGA<br>GTGGTGGTGCTGAGCTTCGAACTGCTGCATGCCCCTGCCACAGTGT<br>GCGGCCCTAAGAAAAGCACCAATCTCGTGAAGAACAAATGCGTGAA<br>CTTCAACTTCAACGGCCTGACCGGCACCGGCGTGCTGACAGAGAGC<br>AACAAGAAGTTCCTGCCATTCCAGCAGTTTGGCCGGGATATCGCCGA<br>TACCACAGACGCCGTTAGAGATCCCCAGACACTGGAAATCCTGGAC<br>ATCACCCCTTGCAGCTTCGGCGGAGTGTCTGTGATCACCCCTGGCA<br>CCAACACCAGCAATCAGGTGGCAGTGCTGTACCAGGACGTGAACTG<br>TACCGAAGTGCCCGTGGCCATTCACGCCGATCAGCTGACACCTACA<br>TGGCGGGTGTACTCCACCGGCAGCAATGTGTTTCAGACCAGAGCCG<br>GCTGTCTGATCGGAGCCGAGCACGTGAACAATAGCTACGAGTGCGA<br>CATCCCCATCGGCGCTGGCATCTGTGCCAGCTACCAGACACAGACA<br>AACAGCCCCGCCTCTGTGGCCAGCCAGAGCATCATTGCCTACACAA<br>TGTCTCTGGGCGCCGAGAACAGCGTGGCCTACTCCAACAACTCTAT<br>CGCTATCCCCACCAACTTCACCATCAGCGTGACCACAGAGATCCTGC<br>CTGTGTCCATGACCAAGACCAGCGTGGACTGCACCATGTACATCTGC<br>GGCGATTCCACCGAGTGCTCCAACCTGCTGCTGCAGTACGGCAGCT<br>TCTGCACCCAGCTGAATAGAGCCCTGACAGGGATCGCCGTGGAACA<br>GGACAAGAACACCCAAGAGGTGTTCGCCCAAGTGAAGCAGATCTA	coding sequence for the SARS-CoV-2 Spike glycoprotein

Table S 2. Genetic parts used in this study

Name	Sequence (5' to 3')	Description
	CAAGACCCCTCCTATCAAGGACTTCGGCGGCTTCAATTTCAGCCAGA TTCTGCCCGATCCTAGCAAGCCCAGCAAGCGGAGCTTCATCGAGGA CCTGCTGTTCAACAAAGTGACACTGGCCGACGCCGGCTTCATCAAG CAGTATGGCGATTGTCTGGGCGACATTGCCGCCAGGGATCTGATTT GCGCCCAGAAGTTTAACGGACTGACAGTGCTGCCTCCTCTGCTGAC CGATGAGATGATCGCCCAGTACACATCTGCCCTGCTGGCCGGCACA ATCACAAGCGGCTGGACATTTGGAGCTGGCGCCGCTCTGCAGATCC CCTTTGCTATGCAGATGGCCTACCGGTTCAACGGCATCGGAGTGAC CCAGAATGTGCTGTACGAGAACCAGAAGCTGATCGCCAACCAGTTCA ACAGCGCCATCGGCAAGATCCAGGACAGCCTGAGCAGCACAGCAAG CGCCCTGGGAAAGCTGCAGGACGTGGTCAACCAGAATGCCCAGGC ACTGAACACCCTGGTCAAGCAGCTGTCCTCCAACTTCGGCGCCATC AGCTCTGTGCTGAACGATATCCTGAGCAGACTGGACAAGGTGGAAG CCGAGGTGCAGATCGACAGACTGATCACCGGAAGGCTGCAGTCCCT GCAGACCTACGTTACCCAGCAGCTGATCAGAGCCGCCGAGATTAGA GCCTCTGCCAATCTGGCCGCCACCAAGATGTCTGAGTGTGTGCTGG GCCAGAGCAAGAGAGTGGACTTTTGCGGCAAGGGCTACCACCTGAT GAGCTTCCCTCAGTCTGCCCCTCACGGCGTGGTGTTTCTGCACGTG ACATACGTGCCCGCTCAAGAGAAGAATTTCACCACCGCTCCAGCCAT CTGCCACGACGGCAAAGCCCACTTTCCTAGAGAAGGCGTGTTCGTG TCCAACGGCACCCATTGGTTCGTGACCCAGCGGAACTTCTACGAGC CCCAGATCATCACCACCGACAACACCTTCGTGTCTGGCAACTGCGAC GTCGTGATCGGCATTGTGAACAATACCGTGTACGACCCTCTGCAGCC CGAGCTGGACAGCTTCAAAGAGGAACTGGATAAGTACTTTAAGAACC ACACAAGCCCCGACGTGGACCTGGGCGATATCAGCGGAATCAATGC CAGCGTCGTGAACATCCAGAAAGAGATCGACCGGCTGAACGAGGTG GCCAAGAATCTGAACGAGAGCCTGATCGACCTGCAAGAACTGGGGA AGTACGAGCAGTACATCAAGTGGCCCAGCGGCCGCTTGGTCCCACG TGGCTCACCCGGATCTGGATACATCCCGGAGGCCCCTAGGGACGGT CAAGCTTACGTGAGAAAGGACGGCGAATGGGTTCTGCTGTCGACCT TCTTGGGA	

Table S 3. Comparison of media compositions

Component	Modified L1 media	Standard L1 media	f/2 media
$NaNO_3$	75 mg L^{-1}	7.5 mg L^{-1}	7.5 mg L^{-1}
$NaH_2PO_4 \cdot H_20$	50 mg L^{-1}	5 mg L^{-1}	5 mg L^{-1}
$FeCl_3 \cdot 6H_20$	3.15 mg L^{-1}	3.15 mg L^{-1}	3.15 mg L^{-1}
$Na_2EDTA \cdot 2H_20$	4.36 mg L^{-1}	4.36 mg L^{-1}	4.36 mg L^{-1}
$CuSO_4 \cdot 5H_20$	2.45 μg L^{-1}	2.5 μg L^{-1}	9.8 μg L^{-1}
$Na_2MoO_4 \cdot 2H_20$	18.9 μg L^{-1}	19.9 μg L^{-1}	6.3 μg L^{-1}
$ZnSO_4 \cdot 7H_20$	22 μg L^{-1}	23 μg L^{-1}	22 μg L^{-1}
$CoCl_2 \cdot 6H_20$	10 μg L^{-1}	11.9 μg L^{-1}	10 μg L^{-1}
$MnCl_2 \cdot 4H_20$	180 μg L^{-1}	178.1 μg L^{-1}	180 μg L^{-1}
H_2SeO_3	1.3 μg L^{-1}	1.29 μg L^{-1}	n/a
$NiSO_4 \cdot 6H_20$	2.7 μg L^{-1}	2.63 μg L^{-1}	n/a
Na_3VO_4	1.84 μg L^{-1}	1.84 μg L^{-1}	n/a
K_2CrO_4	1.94 μg L^{-1}	1.94 μg L^{-1}	n/a
$Na_2SiO_3 \cdot 9H_20$	n/a	30 mg L^{-1}	n/a
Na_2CO_3	n/a	n/a	30 mg L^{-1}

Standard L1 and f/2 media compositions from Guillard, et al., 1962. Guillard, et al., 1975.

Table S 4. Identification of mutations in pSS10 clones by Oxford Nanopore whole plasmid sequencing.

pSS10 Pt clone	Identified mutations relative to predicted sequence
1 (2 clones sequenced)	Both clones have a 5 bp deletion in non-coding region between OriT and CAH
2 (2 clones sequenced)	One clone has an 8 kb duplicated region<br>One clone has a 6 kb duplicated region.
3 (3 clones sequenced)	All have a 9 kb duplicated region generating two intact eGFP ORFs
4 (2 clones sequenced)	No mutations
5 (2 clones sequenced)	All clones have a 1.4 kb duplicated region.<br>One clone has a truncated HASP1 promoter
6 (1 clone sequenced)	Contains a 9 kb duplicated region resulting in one intact eGFP ORF and one truncated eGFP ORF

Table S 5. Results of LFA test strips with algae-RBD or commercially available RBD made in mammalian cells (DAGC174). Control and test alues represent XXXX. Negative serum, confirmed negative by PCR; COVID-19 positive, confirmed positive by PCR; Double vaccination, serum from patients with two vaccine does of Pfizer and confirmed COVID-19 negative before vaccination.

Sample type	Sample	LFA Antigen	Control line	Test line	Visual
Negative serum	VF PRV008	DAGC174	1.870	0.016	Negative
	VF PRV008	algae-RBD	1.908	0.028	Negative
	LR PRV009	DAGC174	1.963	0.016	Negative
	LR PRV009	algae-RBD	1.873	0.018	Negative
COVID-19 positive	HMN37 3682-K21	DAGC174	1.9874	1.1749	Positive
	HMN37 3682-K21	algae-RBD	1.8906	1.2316	Positive
	HMN37 3430-K12	DAGC174	1.9137	0.8916	Positive
	HMN37 3430-K12	algae-RBD	1.8543	0.8246	Positive
Double vaccination (COVID-19 negative)	PSV001-14	DAGC1744	2.0289	1.1948	Positive
	PSV001-14	algae-RBD	1.934	1.0768	Positive
	PSV007-48	DAGC174	1.9979	1.2046	Positive
	PSV007-48	algae-RBD	1.905	1.0778	Positive

Table S 6. List of oligonucleotides used in this study.

Name	Sequence (5‘ to 3‘)	Description
DE3197	ATCTTCCGCTGCATAACCC	Fwd pPtGE31 vector
DE3061	GGTATTCTATTCTCTGATTC	Rev 40SRPS8 promoter
DE3627	ATGGATATACCGAAAAAATCGCTATAATGACCCCGAAGCAGGGTTATGCAGCGGAAG ATGGGGTGGATAAAGAAGAAAGG	Rev 40SRPS8 terminator with pPtGE31 vector homology
DE4130	CCCTGCGATAGACCTTTTCC	Fwd 40SRPS8 promoter
DE5220	TAAATCATAGATGTTCACAATGAGATATTCTTATCTTTACTTGTACAGCTCGTCCATGCC	Rev eGFP with 40SRPS8 terminator homology
DE5239	ATGAATCTTCGTTGTATCCTTCCG	Fwd PtSpike(2P)/PtRBD (universal)
DE5240	TGGTAAATCATAGATGTTCACAATGAGATATTCTTATCTTCAATGGTGATGGTGATGGTG	Rev PtSpike(2P)/PtRBD (universal) with 40SRPS8 terminator homology
DE5241	TTTCCCAGCTACTCGACGCATCAGGCGGGCGGATTGATAGTGAAAACCTTATTCATTGTC	Fwd HASP1 promoter and signal peptide with PtPRA-PH/CH cassette homology
DE5242	AGCCCCAGCCGAGAAG	Rev HASP1 promoter and signal peptide
DE5243	AAAAATTTAATTTTCATTAGTTGCAGTCACTCCGCTTTGGTTTCTCGCGTTTTACGCGCC	Fwd PtPRA-PH/CH cassette with pPtGE31 vector homology
DE5244	ACCGGCTTAAGCTCTGACAATGAATAAGGTTTTCACTATCAATCCGCCCGCCTGATGCGT	Rev PtPRA-PH/CH cassette with HASP1 promoter homology
DE5245	AGATAAGAATATCTCATTGTGAACA	Fwd 40SRPS8 terminator
DE5246	AGTTCGTGGGCCAAGAAACTGACGGCGCGTAAAACGCGAGAAACCAAAGCGGAGTGACTG	Rev pPtGE31 vector with PtPRA-PH/CH cassette homology
DE5247	GCGTTGATCTTGCACCGAAGGAATCAGAGAATAGAATACCATGTTCGTGTTTCTGGTGCT	Fwd HsSpike/HsRBD (universal) with 40SRPS8 promoter homology
DE5248	TAAATCATAGATGTTCACAATGAGATATTCTTATCTTTATCAATGGTGATGGTGATGGTG	Rev HsRBD with 40SRPS8 terminator homology
DE5249	AATCATAGATGTTCACAATGAGATATTCTTATCTTCATTAGTGATGATGATGATGATGTC	Rev HsSpike with pPtGE31 vector homology
DE5250	TTGTTTTCTTGGACTGCGTGAGTTTGGAAAAGGTCTATCGCAGGGGCCCGCCTGATGCGT	Rev PtPRA-PH/CH cassette with 40SRPS8 promoter homology
DE5251	GCGGGATCTGCCTCAGG	Fwd HsSpike sequencing primer 1
DE5252	CCTGTACCGGCTGTTCCG	Fwd HsSpike sequencing primer 2
DE5253	GCCAGCCAGAGCATCATTG	Fwd HsSpike sequencing primer 3
DE5254	ATGTGCTGTACGAGAACCAG	Fwd HsSpike sequencing primer 4
DE5255	AGATTCTGTCCGACGAAGCG	Fwd PtSpike(2P)/PtRBD sequencing primer 1
DE5256	CTCCAAACACACTCCGATTAAC	Fwd PtSpike(2P) sequencing primer 2
DE5257	CTTGGATTCGAAAGTTGGAGG	Fwd PtSpike(2P) sequencing primer 3
DE5258	CCAGACTCAAACGAATTCGC	Fwd PtSpike(2P) sequencing primer 4
DE5259	GGCCTACCGTTTCAACGG	Fwd PtSpike(2P) sequencing primer 5
DE5260	ACCGTGTACGATCCGCTG	Fwd PtSpike(2P) sequencing primer 6
DE5277	TGTATCCTTCCGTTTCTCCTCGCAAGCTTCTCGGCTGGGGCTATGGTGAGCAAGGGCGAG	Fwd eGFP with HASP1 signal peptide homology
DE5323	ACGGAGTAATCGGCGACGC	Rev PtRBD screening primer
DE5326	GAGTTGTACAGCACGGAGTAGTCG	Rev HsRBD screening primer
DE5336	TTCACAATGAGATATTCTTATCTTCAATGGTGGTGATGATGGTGATGGTGATGGTGTCCC	Rev primer to add 10xHis to PtSpike(2P)/PtRBD
DE5337	CACCATCACCATCACCATCATCACCACCATTGAAGATAAGAATATCTCATTGTGAACATC	Fwd primer to add 10xHis to PtSpike(2P)/PtRBD
DE5509	GGTAAATCATAGATGTTCACAATGAGATATTCTTATCTTTATTTTCCGGGCGAAAGGGAC	Rev Fc tag with 40SRPS8 terminator homology
DE5583	CCAATCTGGTCAAGAATAAATGTGTCAATTTTTGAAGATAAGAATATCTCATTGTGAACA	Fwd 40SRPS8 terminator with PtRBD homology to remove 6His and TEV site
DE5584	TGTTCACAATGAGATATTCTTATCTTCAAAAATTGACACATTTATTCTTGACCAGATTGG	Rev PtRBD with 40SRPS8 terminator homology to remove 6His and TEV site
DE5631	ATCCTTCCGTTTCTCCTCGCAAGCTTCTCGGCTGGGGCTTCCCCCATTCTCGGATACTGG	Fwd GST tag with HASP1 signal peptide homology
DE5632	GACTCAGTGGGCTGCACACGTCCCTGGAAGTAGAGGTTTTCGTCGGACTTGGGGGGGTGG	Rev GST tag with TEV site and PtRBD homology
DE5633	GACCACCCCCCCAAGTCCGACGAAAACCTCTACTTCCAGGGACGTGTGCAGCCCACTGAG	Fwd PtRBD with TEV site and GST tag homology
DE5634	CCCTTAATCTTCCAGTATCCGAGAATGGGGGATCCCTGGAAGTAGAGGTTTTCAAAATTG	Rev PtRBD with TEV site and GST tag homology
DE5635	AATAAATGTGTCAATTTTGAAAACCTCTACTTCCAGGGATCCCCCATTCTCGGATACTGG	Fwd GST tag with TEV site and PtRBD homology
DE5636	GGTAAATCATAGATGTTCACAATGAGATATTCTTATCTTCAGTCGGACTTGGGGGGGTGG	Rev GST tag with 40SRPS8 terminator homology
DE5688	GGCAGGTTCCCTGGAAGTAGAGGTTTTCAAAATTGACACATTTATTCTTGACCAGATTGG	Rev PtRBD with TEV site and Fc tag homology
DE5689	CAAGAATAAATGTGTCAATTTTGAAAACCTCTACTTCCAGGGAACCTGCCCGCCCTGCCC	Fwd Fc tag with TEV site and PtRBD homology

References

[1] Daniel J. Giguere, Alexander T. Bahcheli, Samuel S. Slattery, Rushali R. Patel, Martin Flatley, Bogumil J. Karas, David R. Edgell, and Gregory B. Gloor. Telomere-to-telomere genome assembly of *Phaeodactylum tricornutum*. *bioRxiv*, 2021.

[2] Maria Helena Cruz de Carvalho, Hai-Xi Sun, Chris Bowler, and Nam-Hai Chua. Noncoding and coding transcriptome responses of a marine diatom to phosphate fluctuations. *New Phytologist*, 210(2):497–510, 2016.
